# A novel FCTF evaluation and prediction model for food efficacy based on association rule mining

**DOI:** 10.3389/fnut.2023.1170084

**Published:** 2023-08-28

**Authors:** Yaqun Liu, Zhenxia Zhang, Wanling Lin, Hongxuan Liang, Min Lin, Junli Wang, Lianghui Chen, Peikui Yang, Mouquan Liu, Yuzhong Zheng

**Affiliations:** ^1^School of Food Engineering and Biotechnology, Hanshan Normal University, Chaozhou, Guangdong, China; ^2^School of Laboratory Medicine, Youjiang Medical University for Nationalities, Baise, Guangxi, China

**Keywords:** association rule mining, medicinal food, components, target, function

## Abstract

**Introduction:**

Food-components-target-function (FCTF) is an evaluation and prediction model based on association rule mining (ARM) and network interaction analysis, which is an innovative exploration of interdisciplinary integration in the food field.

**Methods:**

Using the components as the basis, the targets and functions are comprehensively explored in various databases and platforms under the guidance of the ARM concept. The focused active components, key targets and preferred efficacy are then analyzed by different interaction calculations. The FCTF model is particularly suitable for preliminary studies of medicinal plants in remote and poor areas.

**Results:**

The FCTF model of the local medicinal food Laoxianghuang focuses on the efficacy of digestive system cancers and neurological diseases, with key targets ACE, PTGS2, CYP2C19 and corresponding active components citronellal, trans-nerolidol, linalool, geraniol, α-terpineol, cadinene and α-pinene.

**Discussion:**

Centuries of traditional experience point to the efficacy of Laoxianghuang in alleviating digestive disorders, and our established FCTF model of Laoxianghuang not only demonstrates this but also extends to its possible adjunctive efficacy in neurological diseases, which deserves later exploration. The FCTF model is based on the main line of components to target and efficacy and optimizes the research level from different dimensions and aspects of interaction analysis, hoping to make some contribution to the future development of the food discipline.

## 1. Introduction

During the battle against coronavirus disease (COVID-19), the role of the medicinal food concept in the prevention and control of pandemics has attracted widespread attention, involving mostly local foods with medicinal value (1). Slogans such as “Food as Medicine, Medicine as Food” have driven the development of functional foods into a trendy form (2). Unfortunately, medicinal foods around the world possess national characteristics, and efficacy studies mostly rely on the inheritance of traditional experiences, which are mainly prevalent in the local area (3). Influenced by factors such as national character, traditional habits and environmental isolation, the medicinal effects of local specialties remain relatively independent and lag behind in development, thus presenting a blind or random process.

Most modern systematic and comprehensive food efficacy studies are based on genomics (4), proteomics (5), metabolomics (6), lipidomics (7), glycomics (8), and other methods and techniques, which are time-consuming, cost substantially, require expensive equipment, and less friendly to traditional specialty foods from poverty-stricken areas. Association rule mining (ARM) is a rule-based machine learning algorithm that can discover hidden patterns and interesting relationships in large databases. Recently, ARM has become a promising technique in multiple fields including biomedical, educational, and social sciences, such as predicting COVID-19 cases and symptom patterns (9, 10), the application of multiresource for MOOC teaching (11), the study of improving English achievement analysis (12) and the investigation of the relationships between shifts in digital skills and cybersecurity awareness (13). Common analysis algorithms and evaluation approaches for ARM include the Apriori algorithm (14), entropy weight method (EWM) (15), technique for order preference by similarity to ideal solution (TOPSIS) (16), support vector machine (SVM) and random forest (RF) (17). This study innovatively developed the ARM and its algorithms to the poorly understood field of medicinal foods and can be applied to other foods as well. By mining the identified food ingredients for their targets and related functions on a big data platform, the active components, key targets and preferred functions are inferred through multidimensional interactions and cross commonalities. To the best of our knowledge, this may be the first application of this model in the field of food efficacy research, which we define as food-components-target-function (FCTF) association rule mining. Data association analysis provides exciting research opportunities and contemporary themes for known food components, which can not only validate traditional empirical medicinal efficacy but also build initial theoretical platforms for future in-depth research.

The FCTF model was carried out on the example of Laoxianghuang, a characteristic medicinal food from Chaozhou, Guangdong Province, China. Laoxianghuang is obtained by fermenting *Citrus medica* L. var. *Sarcodactylis Swingle* for more than several years through a complex process of salting, desalting, sugaring, cooking and drying. Compared to the bitter and spicy raw material *Citrus medica* L. var. *Sarcodactylis Swingle*, the fermented Laoxianghuang not only enhances its edibility but also expands its efficacy as revealed in empirical pharmacology (18–20). It is a local cultural symbol because of its aromatic taste and excellent efficacy. However, due to the remoteness of the region and the limitations of scientific conditions, research on Laoxianghuang is still in the initial stage. Previously, although we explored the components of Laoxianghuang through different methods (18–20), there were obstacles to a more in-depth study under poor and weak scientific research conditions. Therefore, we established an FCTF model to perform a deeper exploration of Laoxianghuang by searching the correlations between components, targets and efficacy. Meanwhile, proposing the FCTF model is expected to help enhance the research connotation and denotation of featured products in the future.

## 2. Materials and methods

### 2.1. Food components library construction

We have previously detected the components of Laoxianghuang and will not repeat it here (18–20). The components of Laoxianghuang were also searched in various literature databases as much as possible, and a component library was obtained by removing the overlap (Supplementary Table S1). The relative contents of the components detected by different methods varied, and we subjectively selected the components with relative contents greater than 1% under each detection method. If a component is represented under different methods, as long as its relative content is higher than 1%, it will be taken into account.

### 2.2. Food-components-target framework construction

The components were used as entry points to query their simplified molecular-input line-entry system (SMILES) numbers on PubChem^1^ (21). The SMILES numbers were imported into SwissTargetPrediction^2^ to retrieve the targets of each component (22), and targets with a probability greater than 0 were selected as the study objects (23) (Supplementary Table S2).

### 2.3. Food-components-target-function model building and analysis

Rough functional enrichment: Gene Ontology (GO) and Kyoto Encyclopedia of Genes and Genomes (KEGG) pathway enrichment analyses were performed by Metascape^3^ after de-duplication of all potential targets (24). The top 20 items with *p* < 0.01 were selected for advanced bubble mapping. GO analysis included molecular function (MF), cellular component (CC) and biological process (BP).

Function Refined Searching: Potential target-related functions and preferred efficacy-related targets were obtained by searching the Comparative Toxicogenomics Database (CTD, http://ctdbase.org/) (25), Online Mendelian Inheritance in Man Database (OMIM, http://www.omim.org) (26), Therapeutic Target Database (TTD, http://db.idrblab.net/ttd/) (27) and GeneCards Database^4^ (28). Accessed on all of the above servers on December 30th, 2022 (Supplementary Tables S3, S4).

### 2.4. Food-components-target-function model verification

The protein crystal structures of the key targets and the corresponding 3D structures of the active components were retrieved from the Protein Data Bank (PDB, http://www.rcsb.org/pdb/) (29) and PubChem (See footnote 1) (21), respectively (PDB IDs are shown in Supplementary Table S5). Application of Cavity-Detection Guided Blind Docking (CB-Dock; http://clab.labshare.cn/cb-dock/php/; accessed on January 9th, 2023) (30) and Mcule 1-Click Docking (https://mcule.com/apps/1-click-docking/; accessed on January 9th, 2023) (31) for evaluation and comparison of molecular docking. The affinity of the docked key target and active components is expressed as binding energy (kcal/mol). Among the four different binding fractions given, the results with more negative values were considered (30).

### 2.5. Data analysis

Descriptive analysis: Microsoft Office Excel 2019 (Microsoft Corporation) was used to perform statistics on the frequency of components, targets and functions, including duplicate and position distribution.

Association rule mining analysis: To obtain high frequency items, analysis was performed using Cytoscape 3.9.0^6^ (32) and a node-weighting scheme using Degree Centrality (33). The association rules are expressed in the form of components → target, target → efficacy, efficacy → target, etc. The front is the basis, and the back is the mining object. The support for association is measured by the Degree value, i.e., the frequency of nodes crossing each other, which can reflect their importance and dependency. This makes the association rule valuable only when the Degree value is greater than 1. Interactions between targets were analyzed using the Search Tool for the Retrieval of Interacting Genes (STRING, https://cn.string-db.org/) with confidence score >0.9 (34). Overlapping targets in different projects were visualized by a Venn diagram (35). The Apriori algorithm further mines the set of frequent items of association rules, with components and functions as index items and targets as analysis items, and assesses its reliability by support, confidence, lift, leverage, and conviction (36):

Support: the probability that the target set Tx, Ty occurs in all items, or the probability that the two target sets Tx → Ty occur in all items. The formula is as follows:


Support(Tx)=P(Tx)/N

Support(Ty)=P(Ty)/N

Support(Tx→Ty)=P(Tx∪Ty)/N

P: number of target occurrences; N: total number of items.

Confidence: the frequency of Ty in the set of items containing Tx. The higher the confidence level, the more relationship between Tx and Ty is considered. The formula is as follows:


Confidence(Tx→Ty)=P(Tx∪Ty)/P(Tx)

Lift: measures how much more often the Tx and Ty occur together rather than them occurring independently. Lift >1: Tx and Ty associated positively; Lift <1: Tx and Ty associated negatively; Lift = 1: Tx and Ty are independent of each other. The formula is as follows:


Lift(Tx→Ty)=Confidence(Tx→Ty)/Support(Ty)

Leverage: the proportion of additional examples covered by both the Tx and Ty above those expected if the premise and consequence were independent of each other. Tx and Ty are independent when the leverage is 0; the greater the leverage, the closer A and B are. The formula is as follows:


$$\begin{array}{l} Leverage\;\left(\mathrm{Tx}\to \mathrm{Ty}\right)=Support\;\left(\mathrm{Tx}\to \mathrm{Ty}\right)-Support\;\left(\mathrm{Tx}\right)\times \\ Support\;\left(\mathrm{Ty}\right) \end{array}$$

Conviction: another measure of departure from independence. The greater the conviction, the closer A and B are. The formula is as follows:


$$Conviction\;\left(\mathrm{Tx}\to \mathrm{Ty}\right)=\left[1-\mathrm{Support}\;\left(\mathrm{Ty}\right)\right]/\left[\begin{array}{l} 1-Confidence\\ \;\left(\mathrm{Tx}\to \mathrm{Ty}\right) \end{array}\right]$$

The screening conditions for the association rules were set as follows: support >20%, confidence >35%, lift >1, leverage >0, conviction >0. Information entropy and weight (%) analysis of the above evaluation parameters based on the EWM was performed, and the performance of each target was ranked according to the distance from positive ideal solution, distance from negative ideal solution and the composite score index by the TOPSIS algorithm (37). SVM and RF machine learning algorithms were constructed using the DALEX R package for bidirectional targets, and the diagnostic performance of both models was evaluated by receiver operating characteristic (ROC) curves and associated area under the curve (AUC) (38).

## 3. Results

### 3.1. Component library construction and analysis

**Figure 1:**
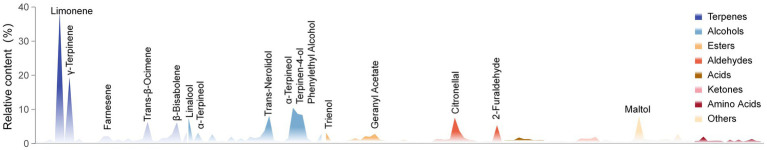
Profile chart of the relative content of Laoxianghuang. Different categories of components are shown in different colors. The actual relative content values of the components in the different methods are plotted, and the values of the same components detected in the different methods are averaged. The specific names of some components with significantly higher content are indicated in the figure.

We systematically studied and summarized the results of different methods to determine the components of Laoxianghuang (Supplementary Table S1). The results showed that there are 156 components in Laoxianghuang, including 32 terpenes, with relatively high contents of limonene, γ-terpinene, trans-β-ocimene, and p-cymene. Twenty-nine alcohols, including linalool, trans-nerolidol, α-terpineo, and terpinen-4-ol were relatively high. Twenty aldehydes reflecting high levels are citronellal and 2-furaldehyde. In addition there are 18 esters, 9 acids, 12 ketones, 16 amino acids, and 20 others in the Laoxianghuang component categories (Figure 1).

### 3.2. Prediction and analysis of potential targets

**Figure 2:**
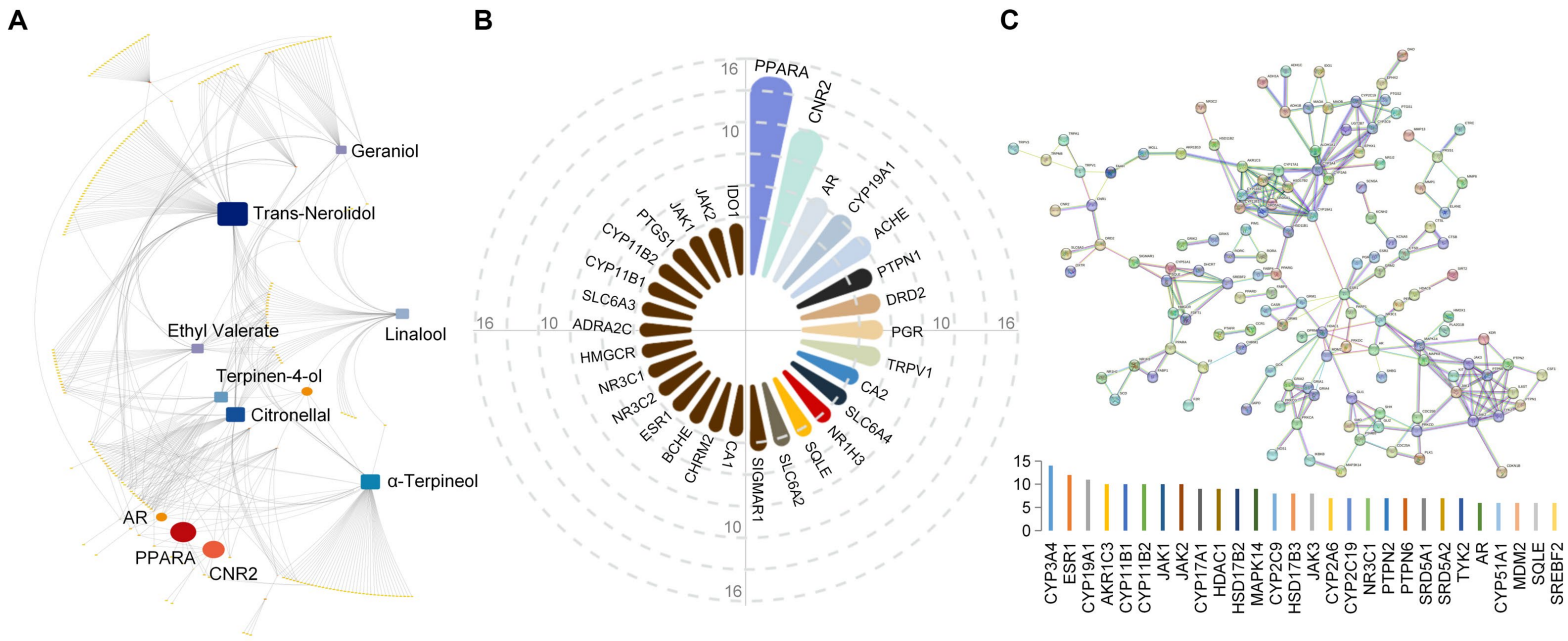
Network construction for Laoxianghuang components and targets. **(A)** Component-target linkage network diagram of Laoxianghuang. Focused elements are highlighted. **(B)** The number of potential target intersections. **(C)** Network diagram of target interactions derived from the STRING input library. The nodes and edges in the network indicate the targets and target–target associations, respectively. The following histogram shows the corresponding Degree values.

First, 35 components with higher relative content were screened out from numerous components of Laoxianghuang according to the screening rules. Then, their SMILES numbers were determined by PubChem, and the target genes corresponding to individual components were retrieved in the SwissTargetPrediction platform according to the SMILES numbers (Supplementary Table S2). A total of 454 predicted targets closely related to components were retrieved, among which trans-nerolidol showed the highest number of related targets, including SQLE, BACE1, PER2 and 82 others. This was followed by citronellal with 52 relevant targets, such as FAAH, CYP19A1, and TRPV1, etc. α-Terpineol, terpinen-4-ol, linalool, geraniol and ethyl valerate were next with 51, 37, 36, 33, and 29 relevant targets, respectively (Figure 2A). The results of focusing on related targets showed that the intersection target with the most components was PPARA, with 14 components associated with it, such as trans-β-ocimene, terpinolene, and cadinene. Subsequently, CNR2 and AR followed in order, with 11 and 7 intersecting components, respectively (Figure 2B). After removing duplicates from the above 454 targets, 236 targets were finally obtained. A high confidence correlation analysis was performed on these duplicate-free targets to identify the target–target interactions. A total of 230 nodes and 244 edges were found in the network excluding unconnected nodes. It indicated 230 interacting targets, with the more interacting target being CYP3A4 (Degree = 14). ESR1 and CYP19A1 followed closely, showing higher target–target interactions with Degree values of 12 and 11, respectively (Figure 2C).

### 3.3. Functional enrichment of potential targets

**Figure 3:**
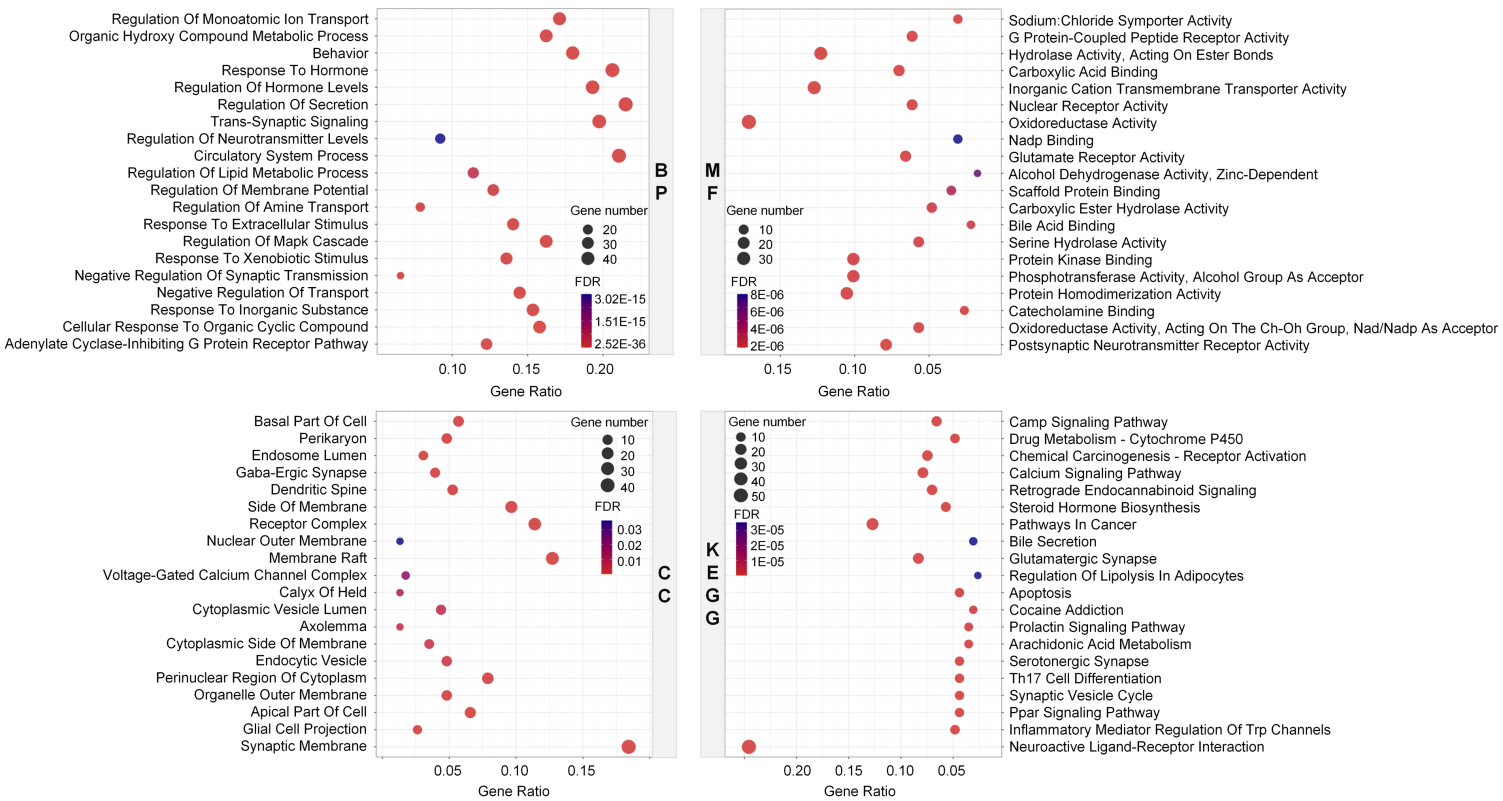
Enrichment of potential targets of Laoxianghuang using GO (BP, MF, and CC) and KEGG analyses. The larger the circles in the figure, the more genes are included. Higher FDR values are indicated with a stronger blue color.

GO and KEGG enrichment analyses were performed on the above predicted potential targets of Laoxianghuang to roughly describe their possible biological functions and signaling pathways. Biological processes (BP) in GO enrichment analysis are mainly involved in regulation of secretion, circulatory system process, response to hormone, etc., and molecular functions (MF) are associated with oxidoreductase activity, inorganic cation transmembrane transporter activity and hydrolase activity, acting on ester bond, etc. Cellular components (CC) are related to the synaptic membrane, membrane raft, receptor complex, etc. The KEGG enrichment results showed that Laoxianghuang was mainly associated with cancer, inflammation, immunity and nervous system, including neuroactive ligand–receptor interaction, glutamatergic synapse, pathways in cancer and steroid hormone biosynthesis (Figure 3).

### 3.4. Refined mining and analysis of functions

**Figure 4:**
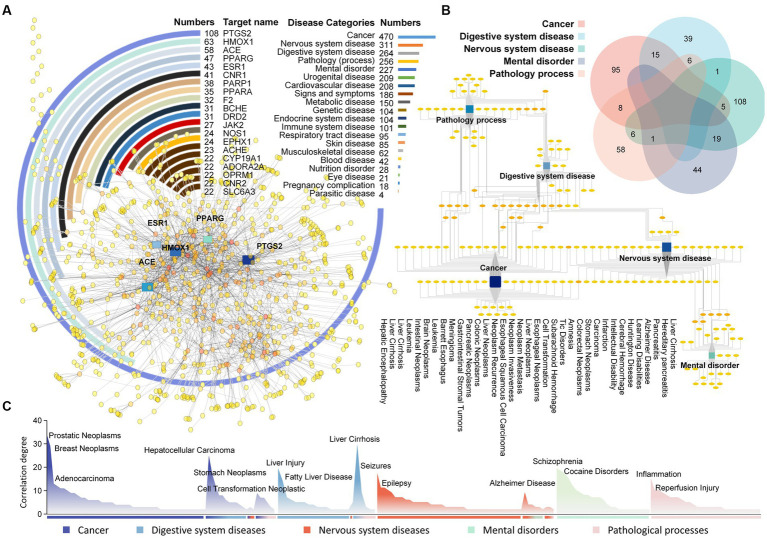
Analysis diagram of Laoxianghuang-related function mining. **(A)** The target-function diagram of Laoxianghuang. The circle diagram shows the Degree of target action. The bar chart shows the functional category Degree of relevance. The network diagram shows the target-function interaction connections, and targets with strong interactions are highlighted. **(B)** Relationships among the five main functions of Laoxianghuang. The multidimensional Venn diagram shows the Degree of overlap among the five functions of Laoxianghuang. The network diagram specifically shows the association between diseases, some of which are listed in the diagram. **(C)** Importance Degree value of specific functions. Some highly correlated disease names are marked in the figure, with different colors representing different disease categories and mixed colors representing cross-linked disease categories.

A deep mining of the efficacy corresponding to each target was conducted, identifying 1,976 relevant diseases with potential associations (Supplementary Table S3). One target can carry different disease profiles; for example, AR has been associated with both cancer and digestive system disorders. PTGS2 is associated with the largest number of diseases, including adenocarcinoma, diabetes mellitus, fever and 108 others (Figure 4A). Among disease categories, cancer presented the strongest association with all targets, appearing 470 times, followed by nervous system diseases and digestive system diseases with 311 and 264, respectively. One disease also matches different targets; for example, nervous system disease can involve CYP19A1, TRPM8, CHRM2, etc.

In this work, the first five associated diseases were selected for further analysis, and each disease was deduplicated to reveal 123, 61, 140, 64, and 79 diseases under the categories of cancer, digestive system diseases, nervous system diseases, mental disorders and pathological processes, respectively. Of these, cancer overlaps with digestive system diseases to a high level, with 15 items belonging to both categories, including stomach neoplasms, colonic neoplasms and intestinal neoplasms. Nervous system diseases and mental disorders also share a high Degree of disease, with 19 diseases such as Alzheimer’s disease, learning disabilities and Tic disorders (Figure 4B). Among all specific diseases, prostatic neoplasms correlated the most with the target (Degree = 34), followed by breast neoplasms, liver cirrhosis, hepatocellular carcinoma, liver injury, schizophrenia, etc. (Figure 4C).

### 3.5. Analysis of association rules for the FCTF model

**Figure 5:**
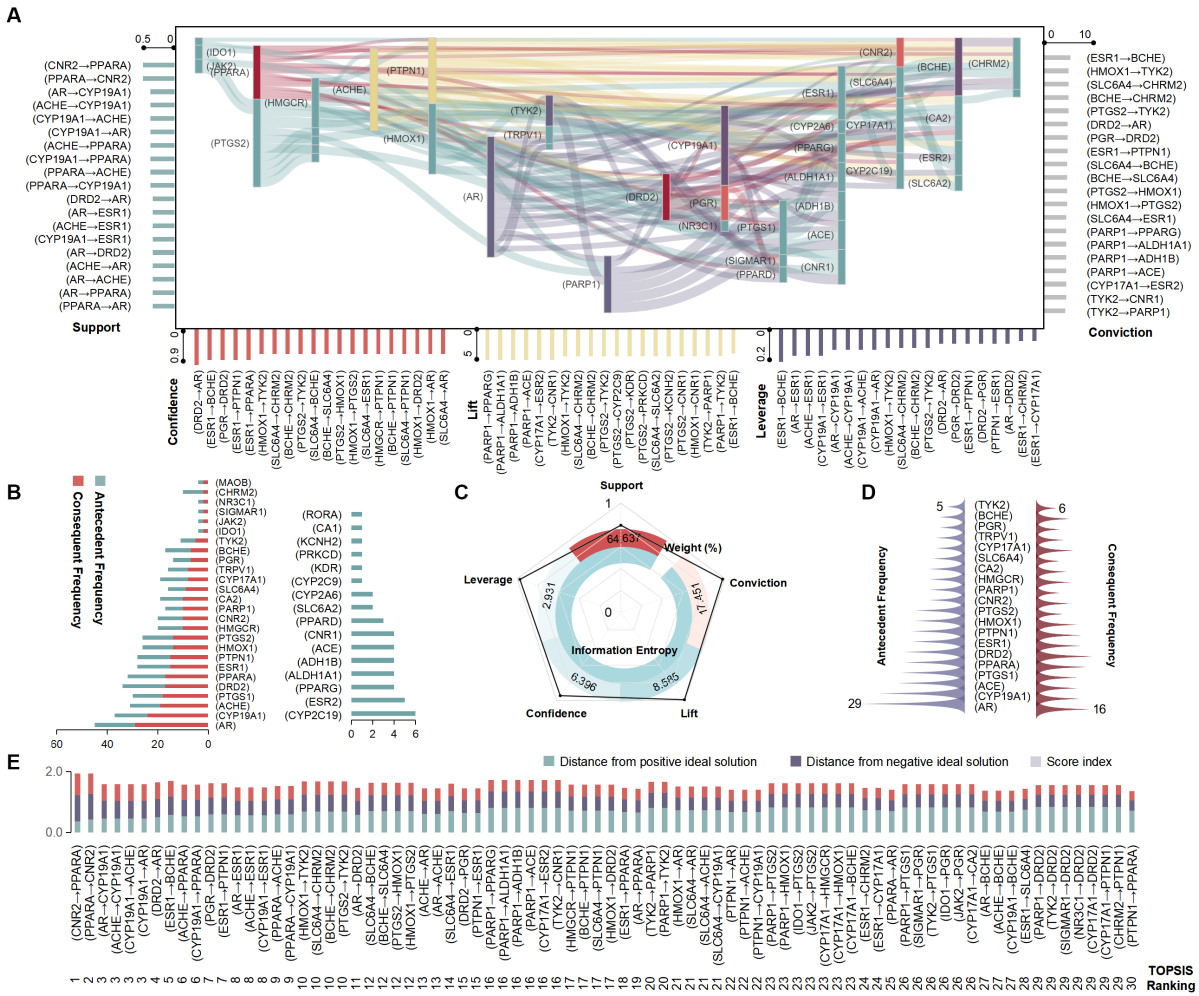
Analysis of association rules for the Laoxianghuang FCTF model. **(A)** Association parameters: left: support; right: conviction, bottom (from left to right): confidence, lift and leverage, all presented in the top 20. Middle: Sankey diagram of association rules. **(B)** Target frequency of antecedent and consequent items by Apriori algorithm. **(C)** Analysis of association rule parameters by EWM. **(D)** Target frequency of antecedent and consequent items by TOPSIS. **(E)** Ranking of target sets by TOPSIS, presented in the top 30.

The Apriori correlation algorithm was used to analyze the crucial link targets using components and functions as index items. Thresholds were set according to support, confidence, lift, leverage and conviction, and a total of 279 sets of target sets were filtered, with a support interval of 20–45%, confidence interval of 35–90%, lift interval of 1.0–4.3, leverage interval of 0.01–0.19, and conviction interval of 1.0–6.6. (CNR2 → PPARA), (AR → (CYP19A1), and (ACHE → CYP19A1) performed better in items of support, all at 43%. (DRD2 → AR), (ESR1 → BCHE), and (PGR → DRD2) showed better confidence levels, all at 90%. (PARP1 → PPARG), (PARP1 → ACE), and (CYP17A1 → ESR2) showed higher lift, all at 4.23. (ESR1 → BCHE), (AR → ESR1) and (ACHE→ESR1) possessed better leverage, all at 0.18. (ESR1 → BCHE), (HMOX1 → TYK2), and (SLC6A4 → CHRM2) displayed better conviction, with (ESR1 → BCHE) at 6.6 and the other two at 6.13 (Figure 5A). All the targets appearing in the antecedent item were duplicated in the consequent item, and the consequent item showed more targets such as (CYP2C19), (ESR2), and (PPARG) compared to the antecedent item (Figure 5B). The results of the EWM showed that the maximum value of indicator weight was support (64.637%), followed by conviction (17.4515%), and the lowest was leverage (2.931%). The best performing information entropy value was leverage (0.991), and the lowest was support (0.797; Figure 5C). The prioritization rankings of the target set obtained using the TOPSIS models demonstrate that (CNR2 → PPARA), (PPARA → CNR2), and (AR → CYP19A1) had the highest priority ranks with scores of 0.708, 0.664, and 0.556, respectively (Figure 5D). The targets that excelled in both antecedent and consequent items were AR, CYP19A1, ACE, etc. (Figure 5E).

### 3.6. Evaluation of the FCTF model

**Figure 6:**
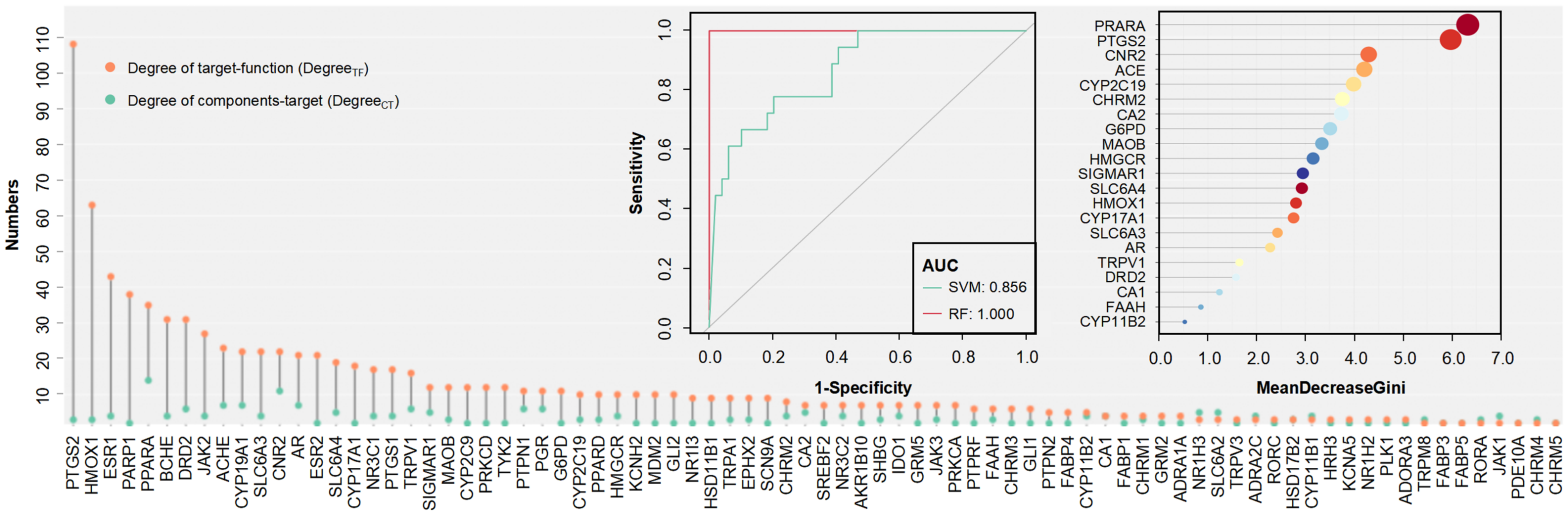
Numbers, RF and SVM classifier of bidirectional targets. Top left: AUC of the two models on the bidirectional targets. Top right: The order of importance of bidirectional targets.

The target is a key link in the design of the FCTF model, so it is necessary to first screen for bidirectional targets (which are associated with more than two components as well as more than two functions) in a large dataset. Finally, 80 bidirectional target sets such as PTGS2 (Degree _CT_ = 3, Degree _TF_ = 108), HMOX1 (Degree _CT_ = 3, Degree _TF_ = 63), ESR1 (Degree _CT_ = 4, Degree _TF_ = 43) were screened (Degree _CT_: Degree of component and target; Degree _TF_: Degree of target and function). Classification models were constructed for this target set, and the results showed that the RF model (AUC = 1.000) achieved higher separation accuracy compared to the SVM model (AUC = 0.856). The importance ranking of the targets in the RF model was filtered according to the Gini coefficient, and PRARA was particularly important, followed by PTGS2, CNR2, ACE, CYP2C19, etc. (Figure 6).

### 3.7. Verification and analysis of the FCTF model

**Figure 7:**
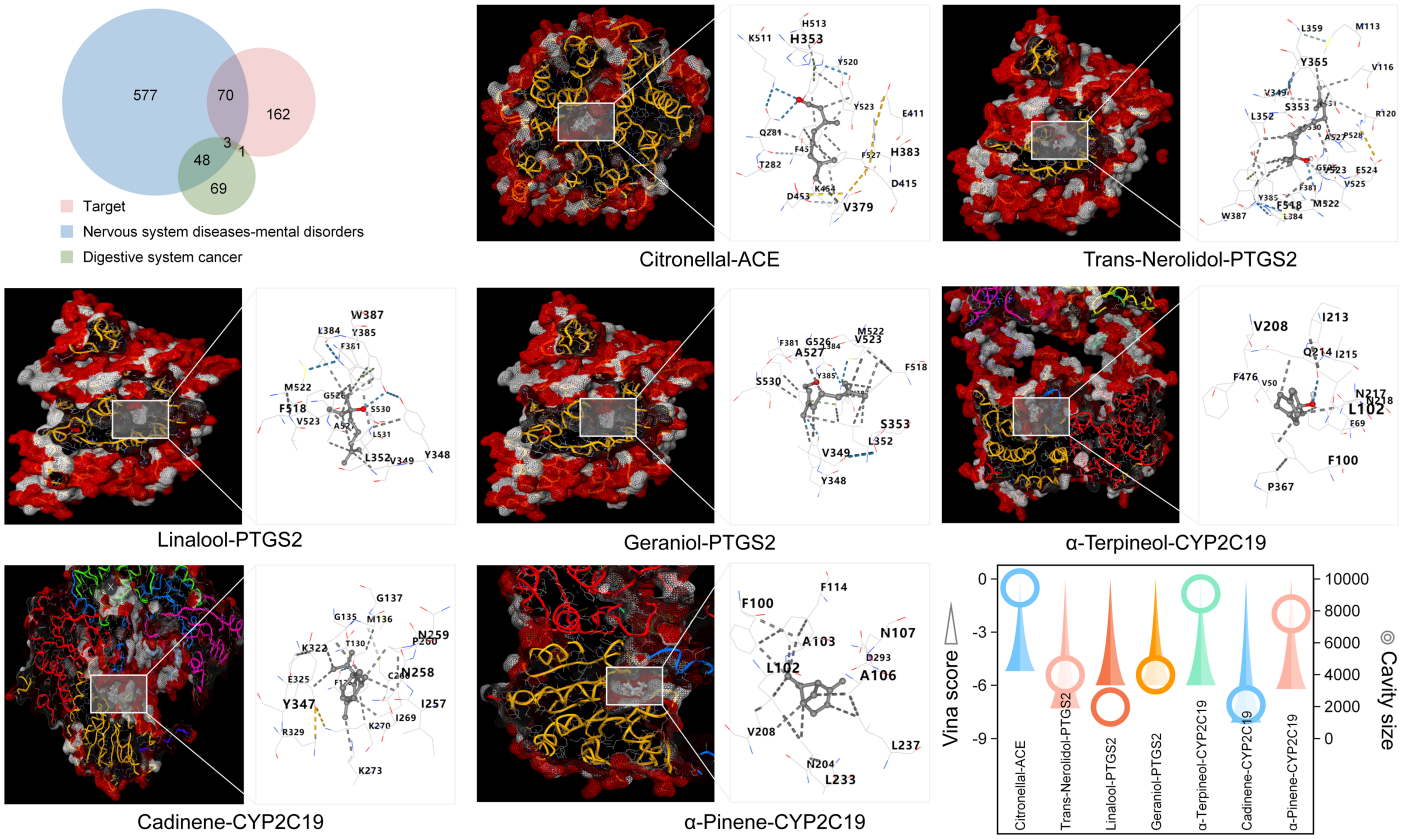
Key target screening and target-component binding model construction. Top left: Venn diagram of targets and associated disease genes. Bottom right: Vina scores and cavity information of the docking simulation pose.

We previously focused on relevant functions by targets to digestive system cancers and neurological diseases (Supplementary Table S4). To further verify this speculation, digestive system cancer and neurological disease genes were retrieved and intersected with the Laoxianghuang target. The results showed that the focus genes for target and digestive system cancers were ACE, PTGS2, CYP2C19, and CYP2A6, while the number of intersections with neurological diseases was 73, and the three shared a focus on ACE, PTGS2 and CYP2C19 (Figure 7, top left). The Laoxianghuang inverse association component search of these three genes revealed that the main relationships were citronellal-ACE, trans-nerolidol-PTGS2, linalool-PTGS2, geraniol-PTGS2, α-terpineol-CYP2C19, cadinene-CYP2C19 and α-pinene-CYP2C19. Notably, these targets were also ranked high in the evaluation of the FCTF model. Molecular docking was performed for the above target component relationships, a 3D diagram between each target and component (detailed information and coordinate locations are shown in Supplementary Table S5) and the specific binding sites are shown in the simulation model (Figure 7). It is now generally accepted that the Vina score is considered to represent the binding activity between the protein and the ligand, with lower compound-target binding free energy indicating more stable binding between the two, and binding energy <−5.0 kcal/mol indicating better binding of the compound to the target site. In addition, the accuracy of docking is improved if the size of the cavity is close to or larger than that of the compound (30). The results of the docking of the synthetically screened compounds and targets in this study showed that their Vina scores were less than−5.0 kcal/mol, and the cavity sizes also displayed strong interactions between the target and components (Figure 7, bottom right).

## 4. Discussion

Association rule mining was introduced as a powerful approach to explore interesting but tangential relationships among components, targets, and effectives of medicinal foods. An FCTF evaluation and prediction model was developed to visualize the association rules of the three and capture the active components, key target and preferred efficacy. Finally, the degree of match between the preferred efficacy-related target and the active components was demonstrated by molecular docking. FCTF analysis identifies anchor targets that link components to efficacy, providing new opportunities for purposeful validation of traditional empirical medicinal efficacy and initiation of future research programs. The model can be applied not only to medicinal foods but also to preliminary studies of other foods.

Laoxianghuang was chosen because this medicinal food has been handed down in the region for hundreds of years and is one of the most ethnically distinctive medicinal foods. Locals believe that it possesses excellent functions, such as soothing the liver, regulating gas, relieving pain in the stomach, eliminating dampness and resolving phlegm (39), and is respected as the first of the “Three Treasures of Chaozhou,” which is a cultural symbol. However, due to geographical factors, economic underdevelopment and human culture, this medicinal food is only prevalent in the local area and has rarely been studied in depth.

We have assayed and validated the components by different detection methods and constructed a components database with as many components as possible to avoid losing the retrieval of targets and efficacy later. In ARM analysis, the primary condition is to obtain “A” information as a way to mine “B,” “C” or more information and explore the interaction relationship between them. The FCTF evaluation and prediction model first obtains the components of food products and then retrieves the corresponding targets of each component and the corresponding efficacy of the targets. The interactions and commonalities between components and targets, targets and targets, and targets and efficacy were also analyzed. In addition to the usual data analysis in Microsoft Excel, Cytoscape network visualization intersection analysis was applied here, and the Degree algorithm was used to filter out key targets and preferred efficacy. It is a common software dedicated to the visualization of interaction network data and is proficient in identifying central objects and subnetworks from complex blind interaction sets, mostly used in bioinformatics (40) and network pharmacology (41). With the help of this analysis software, we can easily focus on the preferred effects of Laoxianghuang on digestive system cancers and neurological diseases, which coincides with the traditional proposal of Laoxianghuang as an adjuvant treatment for digestive diseases (39), while also pointing to its possible adjunctive therapeutic potential for neurological diseases. The raw material for the production of Laoxianghuang is *Citrus medica* L. var. *Sarcodactylis Swingle* of the family *Rutaceae*, whose neolignan derivatives possess hepatoprotective and neuroprotective activities (42). Therefore, future studies on the efficacy of Laoxianghuang could cover neurological diseases in addition to digestive system diseases. Finally, virtual molecular docking, which demonstrates the mode of action of a component to a target is most commonly used in pharmacology (43), drug design (44) and traditional Chinese medicine (45) and is also an important part of computational chemistry and biology, computer science, structural biology, and molecular biology (46). The interaction processes between targets and components are studied from the atomic level by computer simulation techniques to illustrate the availability of the predicted targets and components from the side.

In addition, the association rule for the FCTF models was evaluated by the Apriori algorithm, EWM, TOPSIS, SVM and RF for the FCTF model. Apriori is a classical algorithm for ARM techniques that is widely applied in pharmaceutical and biological analysis (47). The EWM is commonly used in combination with TOPSIS for application assessment in different fields such as management (48), medicine and biology (49). SVM and RF classifiers are also popularly adopted for model evaluation of pharmaceuticals (50). In this study, we combined the multiple methods mentioned above to show that the (CNR2 → PPARA), (PPARA → CNR2), and (AR → CYP19A1) target sets were ranked high, and the targets with more frequent contributions were AR, CYP19A1 and ACE, etc. The association rule and its algorithm is a common technique in the field of data mining to discover correlations and patterns between items in a dataset. Currently, data mining and association rule analysis are used extensively in biomedical research (51–54), but less in the research on medicinal food or other food, thus there exists a potential prospect and wide space in the food field. As the FCTF model constructed by this research, by mining a large amount of food data, and analyzing and exploring the correlations and patterns between components, targets and functions, more valuable information about medicinal foods, health foods or green foods can be revealed to provide a scientific basis for their promotion and development.

The FCTF model proposed in this study is still in the early stage of establishment, and there are several limitations that need to be considered. First, the library of components included in the study needs to be continuously updated, the components determine the later targets and efficacy, and changes in components will lead to potential bias in the model construction. Second, the databases and online platforms for target and efficacy searches are also constantly being updated and need to be researched and updated in time to present more convincing models. In addition, the evaluation index of the FCTF model in this study is mostly based on Degree Centrality, i.e., the higher the value of intersecting nodes is taken into consideration, which is a common weighting scheme for commonality analysis (33). However, it is more subjective and limited, and other rules can be added to refine and improve it according to specific situations in the future. Here we only propose a new theoretical direction for food research, which is the result of data integration, and further experimental validation is needed to explore this model. The composition information of the example Laoxianghuang was not specifically recorded in any of the databases and was mainly obtained by our own detection and the literature, so the FCTF model of Laoxianghuang needed to be re-analyzed and re-established when new compositions appeared.

The FCTF model not only uses ARM theory but also combines analytical tools from systems biology and computational biology, which is a major breakthrough in the interdisciplinary and innovative ideas of food science, and we suggest taking a place for it in modern expensive and time-consuming research. Cross-fertilization of disciplines is an important driver for accelerating science and technology innovation, and strengthening interdisciplinarity and seeking new research paradigms are important ways to promote science and technology innovation (55). The proposed FCTF research model breaks away from the inertia of research in the food field and facilitates its continuous integration with different disciplines to achieve complementary strengths, with a view to promoting the development of the food discipline to a new level.

## Data availability statement

The original contributions presented in the study are included in the article/Supplementary material, further inquiries can be directed to the corresponding author.

## Author contributions

YL and YZ: conceptualization, formal analysis. ZZ and WL: methodology. YL and HL: software, visualization. MiL, JW, PY, and MoL: investigation. YL, HL, and LC: data curation. YL: writing—original draft preparation. YZ: writing—review and editing, supervision, and funding acquisition. All authors contributed to the article and approved the submitted version.

## Funding

This work was supported by the Guangdong Key Laboratory of Functional Substances in Medicinal Edible Resources and Healthcare Products (grant number 2021B1212040015), Scientific projects of key disciplines in Guangdong Province (grant numbers 2021ZDJS042 and 2022ZDJS070), Doctor Initiating Project of the Hanshan Normal University (grant numbers QD202125 and QD20190527), Special Focus Areas for General Universities of Guangdong Province (grant number 2022ZDZX2043), Education Department Project of Guangdong Province (grant numbers 2019-GDXK-0032 and 2020KZDZX1146), and Eastern Guangdong Technological Engineering Research Center (grant number P19004).

## Conflict of interest

The authors declare that they have no known competing financial interests or personal relationships that could have appeared to influence the work reported in this paper.

## Publisher’s note

All claims expressed in this article are solely those of the authors and do not necessarily represent those of their affiliated organizations, or those of the publisher, the editors and the reviewers. Any product that may be evaluated in this article, or claim that may be made by its manufacturer, is not guaranteed or endorsed by the publisher.

## Footnotes

^1^https://pubchem.ncbi.nlm.nih.gov

^2^http://www.swisstargetprediction.ch/

^3^https://metascape.org/gp/index.html

^4^http://www.genecards.org/

^6^https://cytoscape.org/
