# Supplementary material for: A novel FCTF evaluation and prediction model for food efficacy based on association rule mining
Source: Front Nutr. 2023 Aug 28;10:1170084. doi: 10.3389/fnut.2023.1170084 (PMC10493461; doi:10.3389/fnut.2023.1170084)
Supplement: Supplementary file 1 [file Table_1.docx]

**Supplemental Table S1 Summary of the Laoxianghuang components categories and relative content**

| **Component name** | RC (%)^*^ |  | **Component name** | RC (%) |
| --- | --- | --- | --- | --- |
| **Terpenes** |  |  | **Alcohols** |  |
| α-Pinene | 1.025 |  | Linalool | 7.083 |
| α-Thujene | 0.173 |  | 2-(2-Furyl)Ethanol | 0.109 |
| β-Pinene | 0.591 |  | α-Terpineol | 2.613 |
| 7-Methyl-3-Methyleneocta-1,6-Diene | 1.204 |  | Nerol | 0.293 |
| α-Terpinene | 0.37 |  | (1S-Trans)-2-Methyl-5-(1-Methylvinyl)Cyclohex-2-En-1-Ol | 0.043 |
| Limonene | 38.829 |  | Geraniol | 2.201 |
| (Z)-β-Ocimene | 0.273 |  | Benzyl Alcohol | 0.123 |
| γ-Terpinene | 19.435 |  | Nerolidol | 0.373 |
| 3,7-Dimethyl-1,3,6-Octatriene | 0.619 |  | Isophytol | 0.097 |
| Terpinolene | 1.37 |  | Phytol | 1.512 |
| Alloocimene | 0.208 |  | 3-Hexen-1-Ol | 0.09-0.23 |
| 2,4-Dimethylstyrene | 0.205 |  | (-)-Terpinen-4-Ol | 0.15-1.93 |
| 1-Butylcyclohexene | 0.142 |  | 1-Nonanol | 0.25 |
| Bisabolene | 0.616 |  | Gamma Terpineol | 0.03-2.86 |
| L-Caryophyllene | 0.45-3.75 |  | Citronellol | 0.46-1.87 |
| Farnesene | 1.19-2.87 |  | Cineole | 0.64-2.08 |
| Camphene | 0.3-1.18 |  | Ethanol | 1.98-4.68 |
| Valencene | 0.29-2.27 |  | Trans-Nerolidol | 3.54-13.63 |
| Carene | 0.11-1.2 |  | 3-Methyl-1-Butanol | 0.21-0.53 |
| Cadinene | 1.45-1.77 |  | 2-Hexanol | 0.15-0.47 |
| Calamene | 0.15-1.22 |  | Isoamyl Alcohol | 0.01-0.16 |
| 1,5-Dimethyl-1,5-Cyclooctadiene | 0.01-1.98 |  | 2-Furfuryl Alcohol | 0.57-1.65 |
| (Z)-3,7-Dimethylocta-1,3,6,-Triene | 1.31-1.38 |  | α-Terpineol | 8.90-12.46 |
| Trans-β-Ocimene | 5.66-7.55 |  | Terpinen-4-Ol | 6.61-11.64 |
| (+)-2-Carene | 0.19-1.59 |  | Phenylethyl Alcohol | 6.55-8.28 |
| Sabinene | 0.30-0.95 |  | Isopropanol | 0.94-1.27 |
| β-Ocimene | 1.30-2.80 |  | 1-Propanol-D1 | 0.02-0.22 |
| α-Phellandrene | 1.36-1.90 |  | Pentanol | 0.01 |
| Tricyclene | 2.22-3.32 |  | Methanol | 2.09-2.61 |
| β-Bisabolene | 1.65-14.46 |  |  |  |
| Thujene | 0.01 |  | **Aldehydes** |  |
| p-Cymene | 3.206 |  | Hexanal | 0.114 |
|  |  |  | Benzaldehyde | 0.074 |
| **Esters** |  |  | Valeraldehyde | 0.078 |
| Trienol | 2.496 |  | β-Cyclocitral | 0.063 |
| Gamma-Caprolactone | 0.066 |  | 2-Hydroxy-6-Methoxybenzaldehyde | 0.046 |
| Neryl Acetate | 0.063 |  | 3-Furaldehyde | 0.8-0.96 |
| Methyl Salicylate | 0.067 |  | 5-Hydroxymethylfurfural | 0.5-1.64 |
| Methyl Palmitate | 0.108 |  | Cis-Cinnamaldehyde | 0.22-1.21 |
| Methyl Furoate | 0.01-0.85 |  | Trans-Cinnamaldehyde | 0.21-2.1 |
| Methyl Furan-3-Carboxylate | 0.32-1.53 |  | Citronellal | 6.02-8.33 |
| Isopropyl Myristate | 0.13-0.52 |  | 5-Methyl Furfural | 1.32-4.54 |
| Butyl Acetate | 0.87-2.15 |  | Propionaldehyde | 1.11-1.56 |
| Ethyl Valerate | 1.35 |  | Phenylacetaldehyde | 0.62-0.83 |
| Geranyl Acetate | 0.59-3.61 |  | Nonanal | 0.39-0.63 |
| Ethyl Lactate | 0.10-0.92 |  | Heptenal | 0.02-0.17 |
| Methyl 2-Methylbutanoate | 0.01-0.38 |  | Methional | 0.11-0.26 |
| Isobutyl Acetate | 0.01-0.10 |  | Trans-2-Hexenal | 0.05-0.50 |
| Ethyl 2-Methylbutanoate | 0.02-0.09 |  | Isovaleraldehyde | 0.11-0.32 |
| N-Ethyl Propanoate | 0.01-0.17 |  | 2-Furaldehyde | 4.04-5.46 |
| Methyl 5-Methylsalicylate | 0.37-0.61 |  | Butyraldehyde | 0.37-0.45 |
| Methyl 2-Methylbutyrate | 0.03-0.04 |  |  |  |
|  |  |  | **Ketones** |  |
| **Acids** | 10 |  | Damascenone | 0.06 |
| 2-Methyl Butyric Acid | 0.157 |  | Mesityl Oxide | 0.141 |
| 1-Hexanoic Acid | 0.222 |  | Bicyclo[3.2.1]Octan-3-One | 0.115 |
| Geranic Acid | 0.215 |  | Prohydrojasmon | 0.204 |
| Palmitic Acid-13C | 0.994 |  | 2-Pentadecanone | 0.152 |
| Propionic Acid | 0.25-0.83 |  | (1R)-(+)-Nopinone | 0.066 |
| 3-Methylbutanoic Acid | 0.2-0.91 |  | 2,5-Dihydroxyacetophenone | 0.181 |
| Valeric Acid | 0.13-0.22 |  | Acetone | 0.99-2.05 |
| Dimethacetic Acid | 0.20-0.37 |  | Butane-2,3-Dione | 0.78-1.79 |
| Isobutyric Acid | 0.16-0.26 |  | 2-Butanone | 0.11-1.84 |
|  |  |  | Acetoin | 0.40-2.62 |
| **Other Organic Substances** | 20 |  | 2-Heptanone | 0.01-0.02 |
| 2-Methoxy-4-Methylaniline | 0.095 |  |  |  |
| Ethyl Maltol | 0.71 |  | **Amino Acids** |  |
| Cis-Anethol | 0.324 |  | DL-Threonine | 0.522 |
| Eugenol | 0.027 |  | Sericic Acid | 0.595 |
| 5-Isopropyl-2-Methylphenol | 0.186 |  | L-Glutamic Acid | 1.82 |
| (+)-Cedrol | 0.052 |  | Proline | 0.525 |
| Vanillin | 0.085 |  | Glycine-1-13C,15N | 0.594 |
| 2,5-Furandicarbaldehyde | 0.63-1.88 |  | L-Alanine | 0.632 |
| Maltol | 6.55-8.32 |  | Valine | 0.582 |
| 4-Pyridazinamine | 0.2-1.45 |  | L-Methionine | 0.038 |
| Naphthalene | 0.58-0.89 |  | D-Tert-Leucine | 0.917 |
| Chlorcamph | 0.21-0.82 |  | Tyrosine | 0.351 |
| Creosol | 0.17-0.8 |  | L-Lysine | 0.939 |
| 2-Ethylfuran | 0.25-1.39 |  | L-Histidine | 0.315 |
| 2-Acetylthiazole | 0.27-1.42 |  | Argininic Acid | 0.683 |
| Benzo[D]Thiazole | 0.02-0.35 |  | L-Aspartic Acid | 1.114 |
| 1,8-Cineole | 1.33-2.16 |  | Phenylalanine | 0.561 |
| 2-Isobutyl-3-Methylpyrazine | 0.11-0.58 |  | L-Isoleucine | 0.463 |
| 2-Acetylfuran | 0.03-0.07 |  |  |  |
| 2,5-Dimethylfuran | 0.32-0.57 |  | **Other Inorganic Ions** |  |
|  |  |  | Mn, Zn, Ca, Co, Fe, Cr, Cu |  |

^*^ RC: Relative content. The relative content of Laoxianghuang determined by different methods. The relative content of components detected by two or more methods are shown in the interval.
