# Supplementary material for: A novel FCTF evaluation and prediction model for food efficacy based on association rule mining
Source: Front Nutr. 2023 Aug 28;10:1170084. doi: 10.3389/fnut.2023.1170084 (PMC10493461; doi:10.3389/fnut.2023.1170084)
Supplement: Supplementary file 2 [file Table_2.docx]

**Supplemental Table S2 Screening of components and prediction of corresponding targets for Laoxianghuang**

| **Chemical Name** | **Mol ID** | **SMILES** | **Target common name** |
| --- | --- | --- | --- |
| α-Terpineol | MOL000118 | CC1=CCC(CC1)C(C)(C)O | AR, CYP19A1, CA2, CA1, CA4, TRPM8, CHRM2, SLC6A4, NR1H3, PTPN1, NR1I3, SREBF2, NPC1L1, BCHE, ESR1, SQLE, ACHE, CYP51A1, SLC6A2, DRD2, CYP17A1, ESR2, CYP2C19, NR3C2, PTPRF, PTPN2, PLA2G1B, ACP1, AKR1B10, SIGMAR1, NR3C1, TRPV3, ATP12A, PTPN6, SHBG, FABP4, PPARA, FABP3, FABP5, PPARD, FABP1, RORA, HMOX1, HMGCR, CD81, PGR, G6PD, SCD, ADRA2C, HSD11B1, SLC6A3 |
| Terpinen-4-Ol | MOL000608 | CC1=CCC(CC1)(C(C)C)O | AR, CYP19A1, CA2, CA1, CA4, RORC, SREBF2, NPC1L1, NR1H3, HMGCR, TRPM8, ESR2, CHRM2, CYP17A1, PTPN1, RORA, SQLE, DRD2, SLC6A4, CYP51A1, PPARA, PPARD, G6PD, ESR1, ACHE, ADRA2C, HSD17B2, SHBG, SLC6A2, NR3C1, NR3C2, BCHE, PTPRF, PTPN2, PLA2G1B, ACP1, AKR1B10 |
| Maltol | MOL003518 | CC1=C(C(=O)C=CO1)O | TYR, DAO, ERN1 |
| Phenylethyl Alcohol | MOL001300 | C1=CC=C(C=C1)CCO | CA2, MIF, GSR, CYP11B1, CYP11B2 |
| Citronellal | MOL000774 | CC(CCC=C(C)C)CC=O | FAAH, CYP19A1, TRPV1, ADH1A, ADH1B, ADH7, SRD5A1, TRPA1, MAOB, DRD2, HLCS, PGR, MAOA, SIGMAR1, SRD5A2, ALDH1A1, PTGS1, DRD4, CTSK, CTSL, CTSB, SIRT2, PPARG, ABCG2, MTNR1A, MTNR1B, ADH1C, PARP1, PSEN2 PSENEN NCSTN APH1A PSEN1 APH1B, PPARA, CTSD, FABP5, PPARD, FABP1, MMP13, MMP1, HDAC6, HDAC1, CACNA1B, PSMB5, CHRM1, MMP8, AR, FABP4, FABP3, IKBKB, EPHX2, CCND1 CDK4, DBF4 CDC7, JAK1, JAK2, ACE, |
| Trans-β-Ocimene | MOL000201 | CC(=CC/C=C(\C)/C=C)C | PPARA, CNR2, MAOB |
| 2-Furaldehyde | MOL000172 | C1=COC(=C1)C=O |  |
| Trans-Nerolidol | MOL002504 | CC(=CCC/C(=C/CCC(C)(C=C)O)/C)C | SQLE, BACE1, PER2, PGR, HSD17B2, IDO1, GCGR, GRM5, PSEN2 PSENEN NCSTN APH1A PSEN1 APH1B, NR3C1, SLC6A3, HRH3, SLC10A2, CASR, PDE2A, PDE10A, OPRL1, CYP2C9, CYP3A4, NR1H3, JAK3, NPY5R, AVPR1A, MAPK8, PRKCD, TTL, FNTA FNTB, KCNH2, F2R, TRPV1, PABPC1, MAPK14, CCR1, KCNA5, KCNA3, PYGL, GRM2, PGGT1B, CDC25A, CYP11B1, CYP11B2, BRS3, MDM2, LYPLA2, FDFT1, NR1H2, PTPN1, PTGS2, AVPR2, OXTR, CHRM1, CHRM3, KDR, NOS1, AKR1C3, IL6ST, SCN9A, NPY2R, NR1I2, TRPA1, PRCP, ICMT, FKBP1A, ABHD6, EIF2AK1, PGGT1B FNTA, EPHX2, CHRM4, CHRM5, C5AR1, SIGMAR1, GCK, GABRB3 GABRA3 GABRG2, GABRB3 GABRG2 GABRA1, GABRB3 GABRG2 GABRA5, GABRA2 GABRB3 GABRG2, CSF1R, KIT, GABBR2 GABBR1, JAK1, JAK2, PIK3CD, LYPLA1, PRKDC, HSD17B3 |
| Terpinolene | MOL000264 | CC1=CCC(=C(C)C)CC1 | PPARA, CNR2 |
| P-Cymene | MOL000117 | CC1=CC=C(C=C1)C(C)C | CYP2A6, ACHE, TAAR1, PPARA, PTGS1, TRPA1 |
| Methanol | MOL001300 | CO |  |
| γ-Terpinene | MOL000202 | CC1=CCC(=CC1)C(C)C | TRPV1, PLK1, GLI2, GLI1 |
| Ethanol | MOL000776 | CCO |  |
| β-Bisabolene | MOL000968 | CC1=CC[C@H](CC1)C(=C)CCC=C(C)C |  |
| Tricyclene | MOL012110 | CC1(C2CC3C1(C3C2)C)C | SHBG |
| Cadinene | MOL008754 | C[C@H]1CC[C@H]([C@H]2[C@H]1C=C[C@@H](C2)C)C(C)C | PPARA, CNR2, AR, CYP19A1, ESR1, CHRM2, ACHE, SLC6A2, SLC6A4, CYP2C19, BCHE, PTPN1, NR1I3, FAAH, TRPV1, PTGS1, NR1H3 |
| α-Phellandrene | MOL001123 | CC1=CCC(C=C1)C(C)C | PPARA, CNR2, ADORA1, ADORA2A, ADORA3 |
| Eucalyptol | MOL000122 | CC1(C2CCC(O1)(CC2)C)C | CYP19A1, SHH, CYP51A1, ACHE |
| 5-Methyl Furfural | MOL004119 | CC1=CC=C(O1)C=O |  |
| Cis-Ocimene | MOL011618 | CC(=CC/C=C(/C)\C=C)C | PPARA, CNR2, ACHE |
| β-Ocimene | MOL000201 | CC(=CC/C=C(\C)/C=C)C | PPARA, CNR2, MAOB |
| Farnesene | MOL000479 | CC(=CCC/C(=C/C/C=C(\C)/C=C)/C)C |  |
| Propionaldehyde | MOL002541 | CCC=O |  |
| Limonene | MOL000023 | CC1=CCC(CC1)C(=C)C | PPARA, CNR2, NR1H3, CYP19A1 |
| γ-Terpinene | MOL000202 | CC1=CCC(=CC1)C(C)C | TRPV1, PLK1, GLI2, GLI1 |
| Linalool | MOL000920 | CC(=CCCC(C)(C=C)O)C | TRPV3, CA2, CA1, CA4, TRPM8, NR3C2, NR3C1, PGR, SIGMAR1, SLC6A3, SQLE, IDO1, HSD17B2, DRD2, CHRM4, OPRM1, OPRD1, OPRK1, ADRA2C, HMOX1, JAK1, JAK2, PTGS2, KCNA5, PTAFR, SCN5A, SCN9A, PARP1, ADRA1A, HRH3, HRH4, JAK3, TYK2, TNNC1 TNNT2 TNNI3, LRRK2, AR |
| Geraniol | MOL000123 | CC(=CCC/C(=C/CO)/C)C | SQLE, UGT2B7, JAK1, JAK2, PTGS1, PTGS2, PGR, JAK3, TYK2, DRD2, SIGMAR1, IDO1, KCNH2, HMGCR, PRKCG, PRKCD, PRKCA, CYP11B1, CYP11B2, PSEN2 PSENEN NCSTN APH1A PSEN1 APH1B, EPHX2, CNR1, PIM1, PIM3, HSD11B1, NR3C2, MAP3K14, HMOX1, CHRM4, CHRM5, CHRM1, CHRM3, CYP2C9, |
| L-Glutamic Acid | MOL000052 | C(CC(=O)O)[C@@H](C(=O)O)N | GRIK1, GRIA1, ADORA3, GRIK5, SLC1A1, GRM4, GRM3, GRIA4, GRM5, GRM8, GRIK2, GRIK3, GRM1, GRM7, GRIA2, GRM6, GRM2, SLC1A2, SLC6A12, SLC6A1, GABRA1 GABRB2 GABRG2, GABRA3 GABRB2 GABRG2, GABRA2 GABRB2 GABRG2, GABRR1, SLC6A11, SLC22A6, SLC6A13, BBOX1 |
| Phytol | MOL001442 | C[C@@H](CCC[C@@H](C)CCC/C(=C/CO)/C)CCCC(C)C | UGT2B7, CDC25A, CDC25B, AR, GLRA1, RORC, RORA, ICMT, DHCR7, SMO, NPC1L1, PRKCA, MDM2, NR1H2, CNR2, HSD11B2, PGR, CXCR3, MGLL, DRD2 |
| Terpinolene | MOL000264 | CC1=CCC(=C(C)C)CC1 | PPARA, CNR2 |
| Ethyl Valerate | MOL008709 | CCCCC(=O)OCC | CA1, CA2, VDR, EPHX1, CA9, PTPN1, SLC6A3, CTRB1, CDC25A, UGT2B7, IDO1, ADRA2A, ADRA2C, ADRA2B, ADRA1A, F2, PRSS1, CTRC, HMGCR, SLC6A2, ELANE, SLC6A4, NISCH, CYP11B1, GABRA2 GABRB2 GABRG2, CYP11B2, PLA2G6, PDE10A, TAAR1 |
| Myrcene | MOL000197 | CC(=CCCC(=C)C=C)C | PPARA, CNR2 |
| L-Aspartic Acid | MOL000065 | C([C@@H](C(=O)O)N)C(=O)O | EGLN1 |
| α-Pinene | MOL000125 | CC1=CCC2CC1C2(C)C | PPARA, CNR2, ACHE, FAAH, TRPV1, AR, CYP19A1, ESR1, CHRM2, SLC6A2, SLC6A4, CYP2C19, BCHE, PTPN1 |
