# Supplementary material for: A novel FCTF evaluation and prediction model for food efficacy based on association rule mining
Source: Front Nutr. 2023 Aug 28;10:1170084. doi: 10.3389/fnut.2023.1170084 (PMC10493461; doi:10.3389/fnut.2023.1170084)
Supplement: Supplementary file 3 [file Table_3.DOCX]

**Supplemental Table S3 Potential target-related efficacy of Laoxianghuang**

| Target | Disease Name | Disease Categories |
| --- | --- | --- |
| AR | Alopecia | Pathology (anatomical condition), Skin disease |
|  | Androgen-Insensitivity Syndrome | Congenital abnormality, Endocrine system disease, Genetic disease (inborn), Urogenital disease (female), Urogenital disease (male) |
|  | Astrocytoma | Cancer |
|  | Autistic Disorder | Mental disorder |
|  | Breast Neoplasms | Cancer, Skin disease |
|  | Breast Neoplasms, Male | Cancer, Skin disease |
|  | Bulbospinal neuronopathy, X-linked recessive | Genetic disease (inborn), Nervous system disease |
|  | Carcinoma, Hepatocellular | Cancer, Digestive system disease |
|  | Diabetes, Gestational | Endocrine system disease, Metabolic disease, Pregnancy complication |
|  | Genital Diseases, Male | Urogenital disease (male) |
|  | Hypertension | Cardiovascular disease |
|  | Hypospadias 1, X-Linked | Congenital abnormality, Genetic disease (inborn), Urogenital disease (female), Urogenital disease (male) |
|  | Infertility, Female | Urogenital disease (female) |
|  | Infertility, Male | Urogenital disease (male) |
|  | Insulin Resistance | Metabolic disease |
|  | Leukemia | Cancer |
|  | Lung Neoplasms | Cancer, Respiratory tract disease |
|  | Neoplasm Recurrence, Local | Cancer, Pathology (process) |
|  | Prostatic Intraepithelial Neoplasia | Cancer |
|  | Prostatic Neoplasms | Cancer, Urogenital disease (male) |
|  | Prostatic Neoplasms, Castration-Resistant | Cancer, Urogenital disease (male) |
| CYP19A1 | Adenocarcinoma | Cancer |
|  | Amenorrhea | Pathology (process) |
|  | Aromatase deficiency | Congenital abnormality, Endocrine system disease, Genetic disease (inborn), Metabolic disease, Skin disease, Urogenital disease (female), Urogenital disease (male) |
|  | Autistic Disorder | Mental disorder |
|  | Bone Resorption | Musculoskeletal disease |
|  | Breast Neoplasms | Cancer, Skin disease |
|  | Critical Illness | Pathology (process) |
|  | Diabetes, Gestational | Endocrine system disease, Metabolic disease, Pregnancy complication |
|  | Endometriosis | Urogenital disease (female) |
|  | Esophageal Neoplasms | Cancer, Digestive system disease |
|  | Fatty Liver | Digestive system disease |
|  | Fibrosis | Pathology (process) |
|  | Hypogonadism | Endocrine system disease |
|  | Hypopituitarism | Endocrine system disease, Nervous system disease |
|  | Infertility, Female | Urogenital disease (female) |
|  | Neoplasm Metastasis | Cancer, Pathology (process) |
|  | Osteoporosis | Metabolic disease, Musculoskeletal disease |
|  | Ovarian Diseases | Endocrine system disease, Urogenital disease (female) |
|  | Polycystic Ovary Syndrome | Cancer, Endocrine system disease, Urogenital disease (female) |
|  | Prostatic Neoplasms | Cancer, Urogenital disease (male) |
|  | Recurrence | Pathology (process) |
|  | Sexual Infantilism | Congenital abnormality, Endocrine system disease, Urogenital disease (female), Urogenital disease (male) |
| CA2 | Adenocarcinoma | Cancer |
|  | Autism Spectrum Disorder | Mental disorder |
|  | Carcinoma, Hepatocellular | Cancer, Digestive system disease |
|  | Esophageal Squamous Cell Carcinoma | Cancer, Digestive system disease |
|  | Osteopetrosis with renal tubular acidosis | Genetic disease (inborn), Metabolic disease, Musculoskeletal disease, Nervous system disease, Urogenital disease (female), Urogenital disease (male) |
|  | Osteoporosis | Metabolic disease, Musculoskeletal disease |
|  | Stomach Neoplasms | Cancer, Digestive system disease |
| CA1 | Adenocarcinoma | Cancer |
|  | Esophageal Squamous Cell Carcinoma | Cancer, Digestive system disease |
|  | Pneumoconiosis | Occupational disease, Respiratory tract disease |
|  | Stomach Neoplasms | Cancer, Digestive system disease |
| TRPM8 | Migraine Disorders | Nervous system disease |
|  | Migraine without Aura | Nervous system disease |
| CHRM2 | Bradycardia | Cardiovascular disease, Pathology (process) |
|  | Brain Injuries | Nervous system disease, Wounds and injuries |
|  | Diabetes Mellitus, Experimental | Endocrine system disease, Metabolic disease |
|  | Diabetes Mellitus, Type 1 | Endocrine system disease, Immune system disease, Metabolic disease |
|  | Diabetic Neuropathies | Endocrine system disease, Nervous system disease |
|  | Epilepsy | Nervous system disease |
|  | Substance-Related Disorders | Mental disorder, Substance-related disorder |
|  | Urinary Bladder, Neurogenic | Nervous system disease, Signs and symptoms, Urogenital disease (female), Urogenital disease (male) |
| SLC6A4 | Alcoholism | Mental disorder, Substance-related disorder |
|  | Amphetamine-Related Disorders | Mental disorder, Substance-related disorder |
|  | Anxiety Disorders | Mental disorder |
|  | Asperger Syndrome | Mental disorder |
|  | Autistic Disorder | Mental disorder |
|  | Bipolar Disorder | Mental disorder |
|  | Cocaine-Related Disorders | Mental disorder, Substance-related disorder |
|  | Cognition Disorders | Mental disorder |
|  | Depressive Disorder | Mental disorder |
|  | Depressive Disorder, Major | Mental disorder |
|  | Hypertension, Pulmonary | Cardiovascular disease, Respiratory tract disease |
|  | Memory Disorders | Nervous system disease, Signs and symptoms |
|  | Mood Disorders | Mental disorder |
|  | Obsessive-Compulsive Disorder | Mental disorder |
|  | Personality Disorders | Mental disorder |
|  | Schizophrenia | Mental disorder |
|  | Substance-Related Disorders | Mental disorder, Substance-related disorder |
|  | Substance Withdrawal Syndrome | Mental disorder, Substance-related disorder |
|  | Sudden Infant Death | Pathology (process) |
| NR1H3 | Chemical and Drug Induced Liver Injury | Digestive system disease |
|  | Cholestasis | Digestive system disease |
|  | Fatty Liver | Digestive system disease |
| PTPN1 | Diabetes Mellitus, Type 2 | Endocrine system disease, Metabolic disease |
|  | Hodgkin Disease | Cancer, Immune system disease, Lymphatic disease |
|  | Hyperinsulinism | Metabolic disease |
|  | Hypertension | Cardiovascular disease |
|  | Inflammation | Pathology (process) |
|  | Insulin Resistance | Metabolic disease |
|  | Liver Cirrhosis, Experimental | Digestive system disease, Pathology (process) |
|  | Lymphoma, B-Cell | Cancer, Immune system disease, Lymphatic disease |
|  | Mediastinal Neoplasms | Cancer, Respiratory tract disease |
|  | Obesity | Nutrition disorder, Signs and symptoms |
|  | Rett Syndrome | Genetic disease (inborn), Nervous system disease |
| NR1I3 | Adenoma, Liver Cell | Cancer, Digestive system disease |
|  | Chemical and Drug Induced Liver Injury | Digestive system disease |
|  | Diabetes Mellitus | Endocrine system disease, Metabolic disease |
|  | Hepatomegaly | Digestive system disease, Pathology (anatomical condition) |
|  | Liver Neoplasms | Cancer, Digestive system disease |
|  | Liver Neoplasms, Experimental | Cancer, Digestive system disease |
|  | Metabolic Syndrome | Metabolic disease |
|  | Obesity | Nutrition disorder, Signs and symptoms |
|  | PreCancerous Conditions | Cancer |
| SREBF2 | Breast Neoplasms | Cancer, Skin disease |
|  | Carcinoma, Hepatocellular | Cancer, Digestive system disease |
|  | Disease Progression | Pathology (process) |
|  | Insulin Resistance | Metabolic disease |
|  | Kidney Failure, Chronic | Pathology (process), Urogenital disease (female), Urogenital disease (male) |
|  | Neoplasm Metastasis | Cancer, Pathology (process) |
|  | Stomach Neoplasms | Cancer, Digestive system disease |
| BCHE | Alzheimer Disease | Mental disorder, Nervous system disease |
|  | Apnea | Respiratory tract disease, Signs and symptoms |
|  | Bamforth syndrome | Congenital abnormality, Endocrine system disease, Mouth disease, Musculoskeletal disease, Skin disease |
|  | Breast Neoplasms | Cancer, Skin disease |
|  | Butyrylcholinesterase deficiency | Genetic disease (inborn), Metabolic disease, Respiratory tract disease, Signs and symptoms |
|  | Carcinoma, Renal Cell | Cancer, Urogenital disease (female), Urogenital disease (male) |
|  | Cardiotoxicity | Cardiovascular disease, Pathology (process), Wounds and injuries |
|  | Cocaine-Related Disorders | Mental disorder, Substance-related disorder |
|  | Cognition Disorders | Mental disorder |
|  | Death | Pathology (process) |
|  | Drug-Related Side Effects and Adverse Reactions |  |
|  | Epilepsy, Tonic-Clonic | Nervous system disease |
|  | Fasciculation | Nervous system disease, Signs and symptoms |
|  | Glioblastoma | Cancer |
|  | Hyperkinesis | Nervous system disease, Signs and symptoms |
|  | Hypertriglyceridemia | Metabolic disease |
|  | Multiple Sclerosis | Immune system disease, Nervous system disease |
|  | Muscular Dystrophies | Genetic disease (inborn), Musculoskeletal disease, Nervous system disease |
|  | Neuroblastoma | Cancer |
|  | Neurotoxicity Syndromes | Nervous system disease |
|  | Obesity | Nutrition disorder, Signs and symptoms |
|  | Organophosphate Poisoning |  |
|  | Paralysis | Nervous system disease, Signs and symptoms |
|  | Paresis | Nervous system disease, Signs and symptoms |
|  | Parkinson Disease, Secondary | Nervous system disease |
|  | Poisoning |  |
|  | Postoperative Complications | Pathology (process) |
|  | Seizures | Nervous system disease, Signs and symptoms |
|  | Sleep Apnea Syndromes | Nervous system disease, Respiratory tract disease |
|  | Tachycardia | Cardiovascular disease, Pathology (process) |
|  | Trismus | Nervous system disease, Signs and symptoms |
| ESR1 | Adenocarcinoma | Cancer |
|  | Alzheimer Disease | Mental disorder, Nervous system disease |
|  | Astrocytoma | Cancer |
|  | Atherosclerosis | Cardiovascular disease |
|  | Bone Diseases | Musculoskeletal disease |
|  | Bone Diseases, Metabolic | Metabolic disease, Musculoskeletal disease |
|  | Breast Neoplasms | Cancer, Skin disease |
|  | Carcinoma | Cancer |
|  | Carcinoma, Hepatocellular | Cancer, Digestive system disease |
|  | Cell Transformation, Neoplastic | Cancer, Pathology (process) |
|  | Coronary Artery Disease | Cardiovascular disease |
|  | Disease Models, Animal | Animal disease |
|  | Disease Progression | Pathology (process) |
|  | Endometrial Neoplasms | Cancer, Urogenital disease (female) |
|  | Female Urogenital Diseases | Urogenital disease (female) |
|  | Feminization | Signs and symptoms |
|  | Glomerulonephritis | Urogenital disease (female), Urogenital disease (male) |
|  | Idiopathic Pulmonary Fibrosis | Respiratory tract disease |
|  | Infertility, Female | Urogenital disease (female) |
|  | Infertility, Male | Urogenital disease (male) |
|  | Leiomyoma | Cancer |
|  | Leukemia | Cancer |
|  | Liver Neoplasms | Cancer, Digestive system disease |
|  | Liver Neoplasms, Experimental | Cancer, Digestive system disease |
|  | Lung Neoplasms | Cancer, Respiratory tract disease |
|  | Mammary Neoplasms, Animal | Animal disease, Cancer |
|  | Mammary Neoplasms, Experimental | Cancer |
|  | Mesothelioma | Cancer |
|  | Migraine Disorders | Nervous system disease |
|  | MIGRAINE WITH OR WITHOUT AURA, SUSCEPTIBILITY TO, 1 | Nervous system disease |
|  | Mitochondrial Diseases | Metabolic disease |
|  | Musculoskeletal Pain | Musculoskeletal disease, Signs and symptoms |
|  | Myocardial Infarction | Cardiovascular disease, Pathology (process) |
|  | Neoplasm Invasiveness | Cancer, Pathology (process) |
|  | Neoplasm Metastasis | Cancer, Pathology (process) |
|  | Neoplasm Recurrence, Local | Cancer, Pathology (process) |
|  | Neuroblastoma | Cancer |
|  | Obesity | Nutrition disorder, Signs and symptoms |
|  | Oligospermia | Urogenital disease (male) |
|  | Prostatic Intraepithelial Neoplasia | Cancer |
|  | Prostatic Neoplasms | Cancer, Urogenital disease (male) |
|  | Psychoses, Substance-Induced | Mental disorder, Substance-related disorder |
|  | Urinary Bladder Neoplasms | Cancer, Urogenital disease (female), Urogenital disease (male) |
| SQLE | Liver Neoplasms, Experimental | Cancer, Digestive system disease |
| ACHE | Alzheimer Disease | Mental disorder, Nervous system disease |
|  | Amphetamine-Related Disorders | Mental disorder, Substance-related disorder |
|  | Amyloidosis | Metabolic disease |
|  | Anemia | Blood disease |
|  | Breast Neoplasms | Cancer, Skin disease |
|  | Carcinoma, Renal Cell | Cancer, Urogenital disease (female), Urogenital disease (male) |
|  | Colorectal Neoplasms | Cancer, Digestive system disease |
|  | Drug-Related Side Effects and Adverse Reactions |  |
|  | Growth Disorders | Pathology (process) |
|  | Learning Disabilities | Mental disorder, Nervous system disease, Signs and symptoms |
|  | Liver Cirrhosis, Experimental | Digestive system disease, Pathology (process) |
|  | Memory Disorders | Nervous system disease, Signs and symptoms |
|  | Movement Disorders | Nervous system disease |
|  | Muscular Dystrophies | Genetic disease (inborn), Musculoskeletal disease, Nervous system disease |
|  | Nervous system diseases | Nervous system disease |
|  | Obesity | Nutrition disorder, Signs and symptoms |
|  | Organophosphate Poisoning |  |
|  | Poisoning |  |
|  | Prenatal Injuries | Pregnancy complication |
|  | Prostatic Neoplasms | Cancer, Urogenital disease (male) |
|  | Seizures | Nervous system disease, Signs and symptoms |
|  | Tremor | Nervous system disease, Signs and symptoms |
|  | Urinary Bladder Neoplasms | Cancer, Urogenital disease (female), Urogenital disease (male) |
| SLC6A2 | Hypertension | Cardiovascular disease |
|  | Orthostatic Intolerance | Nervous system disease, Signs and symptoms |
|  | Seizures | Nervous system disease, Signs and symptoms |
| DRD2 | Amphetamine-Related Disorders | Mental disorder, Substance-related disorder |
|  | Anxiety Disorders | Mental disorder |
|  | Attention Deficit Disorder with Hyperactivity | Mental disorder |
|  | Basal Ganglia Diseases | Nervous system disease |
|  | Bradycardia | Cardiovascular disease, Pathology (process) |
|  | Catalepsy | Nervous system disease, Signs and symptoms |
|  | Cocaine-Related Disorders | Mental disorder, Substance-related disorder |
|  | Cognition Disorders | Mental disorder |
|  | Developmental Disabilities | Mental disorder |
|  | Disruptive, Impulse Control, and Conduct Disorders | Mental disorder |
|  | Dyskinesia, Drug-Induced | Nervous system disease, Signs and symptoms |
|  | Dyskinesias | Nervous system disease, Signs and symptoms |
|  | Heroin Dependence | Mental disorder, Substance-related disorder |
|  | Hyperkinesis | Nervous system disease, Signs and symptoms |
|  | Hyperprolactinemia | Endocrine system disease, Nervous system disease |
|  | Hypertension | Cardiovascular disease |
|  | Hypotension | Cardiovascular disease |
|  | Language Disorders | Nervous system disease, Signs and symptoms |
|  | Memory Disorders | Nervous system disease, Signs and symptoms |
|  | Movement Disorders | Nervous system disease |
|  | Pain | Signs and symptoms |
|  | Parkinson Disease | Nervous system disease |
|  | Parkinsonian Disorders | Nervous system disease |
|  | Pituitary Neoplasms | Cancer, Endocrine system disease, Nervous system disease |
|  | Schizophrenia | Mental disorder |
|  | Seizures | Nervous system disease, Signs and symptoms |
|  | Substance-Related Disorders | Mental disorder, Substance-related disorder |
|  | Substance Withdrawal Syndrome | Mental disorder, Substance-related disorder |
|  | Tachycardia | Cardiovascular disease, Pathology (process) |
|  | Tobacco Use Disorder | Mental disorder, Substance-related disorder |
|  | Weight Gain | Signs and symptoms |
| CYP17A1 | Adrenal Hyperplasia, Congenital | Congenital abnormality, Endocrine system disease, Genetic disease (inborn), Metabolic disease, Urogenital disease (female), Urogenital disease (male) |
|  | Adrenal hyperplasia, congenital, type 5 | Congenital abnormality, Endocrine system disease, Genetic disease (inborn), Metabolic disease, Urogenital disease (female), Urogenital disease (male) |
|  | Amenorrhea | Pathology (process) |
|  | Breast Neoplasms | Cancer, Skin disease |
|  | Carcinoma, Hepatocellular | Cancer, Digestive system disease |
|  | Hyperkalemia | Metabolic disease |
|  | Hypertension, Malignant | Cardiovascular disease |
|  | Hypogonadism | Endocrine system disease |
|  | Hypopituitarism | Endocrine system disease, Nervous system disease |
|  | Infertility, Female | Urogenital disease (female) |
|  | Infertility, Male | Urogenital disease (male) |
|  | Liver Cirrhosis, Experimental | Digestive system disease, Pathology (process) |
|  | Neuralgia | Nervous system disease, Signs and symptoms |
|  | Non-alcoholic Fatty Liver Disease | Digestive system disease |
|  | Oligomenorrhea | Pathology (process) |
|  | Ovarian Cysts | Cancer, Endocrine system disease, Urogenital disease (female) |
|  | Prostatic Neoplasms | Cancer, Urogenital disease (male) |
|  | Sexual Dysfunctions, Psychological | Mental disorder |
| ESR2 | Adenocarcinoma | Cancer |
|  | Arrhythmias, Cardiac | Cardiovascular disease, Pathology (process) |
|  | Astrocytoma | Cancer |
|  | Atrial Fibrillation | Cardiovascular disease, Pathology (process) |
|  | Breast Neoplasms | Cancer, Skin disease |
|  | Carcinoma | Cancer |
|  | Carcinoma, Transitional Cell | Cancer |
|  | Cocaine-Related Disorders | Mental disorder, Substance-related disorder |
|  | Colorectal Neoplasms | Cancer, Digestive system disease |
|  | Coronary Artery Disease | Cardiovascular disease |
|  | Coronary Disease | Cardiovascular disease |
|  | Endometrial Neoplasms | Cancer, Urogenital disease (female) |
|  | Endometriosis | Urogenital disease (female) |
|  | Infertility, Female | Urogenital disease (female) |
|  | Lithiasis | Pathology (process) |
|  | Mammary Neoplasms, Animal | Animal disease, Cancer |
|  | Mammary Neoplasms, Experimental | Cancer |
|  | Medulloblastoma | Cancer |
|  | Prostatic Intraepithelial Neoplasia | Cancer |
|  | Prostatic Neoplasms | Cancer, Urogenital disease (male) |
|  | Urinary Bladder Neoplasms | Cancer, Urogenital disease (female), Urogenital disease (male) |
| CYP2C19 | Acute Kidney Injury | Urogenital disease (female), Urogenital disease (male) |
|  | Atrial Fibrillation | Cardiovascular disease, Pathology (process) |
|  | Cardiovascular Diseases | Cardiovascular disease |
|  | Chemical and Drug Induced Liver Injury | Digestive system disease |
|  | Drug Metabolism, Poor, -Related | Genetic disease (inborn), Metabolic disease |
|  | Genetic Predisposition to Disease | Pathology (process) |
|  | Kidney Failure, Chronic | Pathology (process), Urogenital disease (female), Urogenital disease (male) |
|  | Primary Ovarian Insufficiency | Endocrine system disease, Urogenital disease (female) |
|  | Prostatic Neoplasms | Cancer, Urogenital disease (male) |
|  | Thrombosis | Cardiovascular disease |
| NR3C2 | Cardiomyopathy, Dilated | Cardiovascular disease, Genetic disease (inborn) |
|  | Endometriosis | Urogenital disease (female) |
|  | Heart Failure | Cardiovascular disease |
|  | Hypertension, Early-Onset, Autosomal Dominant, with Severe Exrbation in Pregnancy | Cardiovascular disease |
|  | Myocardial Infarction | Cardiovascular disease, Pathology (process) |
|  | Pseudohypoaldosteronism | Genetic disease (inborn), Urogenital disease (female), Urogenital disease (male) |
|  | Ventricular Dysfunction, Left | Cardiovascular disease |
| PTPRF | BREASTS AND/OR NIPPLES, APLASIA OR HYPOPLASIA OF, 2 | Skin disease |
|  | Disease Progression | Pathology (process) |
|  | Heart Failure | Cardiovascular disease |
|  | Hypertrophy, Left Ventricular | Cardiovascular disease, Pathology (anatomical condition) |
|  | Mesothelioma, Malignant | Cancer, Respiratory tract disease |
|  | Stomach Neoplasms | Cancer, Digestive system disease |
| PTPN2 | Arthritis, Rheumatoid | Connective tissue disease, Immune system disease, Musculoskeletal disease |
|  | Colitis, Ulcerative | Digestive system disease |
|  | Crohn Disease | Digestive system disease |
|  | Leukemia, T-Cell | Cancer, Immune system disease, Lymphatic disease |
|  | Precursor T-Cell Lymphoblastic Leukemia-Lymphoma | Cancer, Immune system disease, Lymphatic disease |
| AKR1B10 | Carcinoma, Hepatocellular | Cancer, Digestive system disease |
|  | Colorectal Neoplasms | Cancer, Digestive system disease |
|  | Dermatitis, Contact | Skin disease |
|  | Endometrial Neoplasms | Cancer, Urogenital disease (female) |
|  | Melanoma | Cancer |
|  | Neoplasm Invasiveness | Cancer, Pathology (process) |
|  | Neoplasms | Cancer |
| SIGMAR1 | Amnesia | Mental disorder, Nervous system disease, Signs and symptoms |
|  | Amyotrophic Lateral Sclerosis | Metabolic disease, Nervous system disease |
|  | AMYOTROPHIC LATERAL SCLEROSIS 16, JUVENILE | Metabolic disease, Nervous system disease |
|  | Cocaine-Related Disorders | Mental disorder, Substance-related disorder |
|  | Encephalomyelitis, Autoimmune, Experimental | Immune system disease, Nervous system disease |
|  | Frontotemporal Lobar Degeneration | Mental disorder, Metabolic disease, Nervous system disease |
|  | HIV Infections | Immune system disease, Viral disease |
|  | Learning Disabilities | Mental disorder, Nervous system disease, Signs and symptoms |
|  | Memory Disorders | Nervous system disease, Signs and symptoms |
|  | Prenatal Exposure Delayed Effects | Pregnancy complication |
|  | Seizures | Nervous system disease, Signs and symptoms |
|  | Spinal muscular atrophy, Jerash type | Nervous system disease |
| NR3C1 | 46, XX Disorders of Sex Development | Congenital abnormality, Endocrine system disease, Urogenital disease (female), Urogenital disease (male) |
|  | Atrial Fibrillation | Cardiovascular disease, Pathology (process) |
|  | Bipolar Disorder | Mental disorder |
|  | Cocaine-Related Disorders | Mental disorder, Substance-related disorder |
|  | Depressive Disorder | Mental disorder |
|  | Drug-Related Side Effects and Adverse Reactions |  |
|  | Endometriosis | Urogenital disease (female) |
|  | Glucocorticoid Receptor Deficiency | Genetic disease (inborn), Metabolic disease |
|  | Glucose Metabolism Disorders | Metabolic disease |
|  | Hypertension | Cardiovascular disease |
|  | Hypokalemia | Metabolic disease |
|  | Lung Diseases, Obstructive | Respiratory tract disease |
|  | Mood Disorders | Mental disorder |
|  | Ocular Hypertension | Eye disease |
|  | Prostatic Neoplasms | Cancer, Urogenital disease (male) |
|  | Psychotic Disorders | Mental disorder |
|  | Schizophrenia | Mental disorder |
| TRPV3 | Hyperplasia | Pathology (process) |
|  | OLMSTED SYNDROME 1 | Genetic disease (inborn), Skin disease |
|  | PALMOPLANTAR KERATODERMA, NONEPIDERMOLYTIC, FOCAL 2 | Genetic disease (inborn), Skin disease |
| PTPN6 | Calcinosis | Metabolic disease |
|  | Heart Valve Diseases | Cardiovascular disease |
|  | Respiratory Hypersensitivity | Immune system disease, Respiratory tract disease |
| SHBG | Alcoholism | Mental disorder, Substance-related disorder |
|  | Cardiomyopathy, Dilated | Cardiovascular disease, Genetic disease (inborn) |
|  | Diabetes Mellitus, Type 2 | Endocrine system disease, Metabolic disease |
|  | Erectile Dysfunction | Mental disorder, Urogenital disease (male) |
|  | Mesothelioma, Malignant | Cancer, Respiratory tract disease |
|  | Metabolic Syndrome | Metabolic disease |
|  | Prostatic Neoplasms | Cancer, Urogenital disease (male) |
| FABP4 | Carcinoma | Cancer |
|  | Dermatitis, Contact | Skin disease |
|  | Kidney Diseases | Urogenital disease (female), Urogenital disease (male) |
|  | Mammary Neoplasms, Animal | Animal disease, Cancer |
|  | Mammary Neoplasms, Experimental | Cancer |
| PPARA | Abdominal obesity metabolic syndrome | Metabolic disease |
|  | Carcinoma | Cancer |
|  | Cardiomegaly | Cardiovascular disease, Pathology (anatomical condition) |
|  | Chemical and Drug Induced Liver Injury | Digestive system disease |
|  | Crohn Disease | Digestive system disease |
|  | Dermatitis, Atopic | Genetic disease (inborn), Immune system disease, Skin disease |
|  | Diabetes Mellitus, Experimental | Endocrine system disease, Metabolic disease |
|  | Diabetes Mellitus, Type 2 | Endocrine system disease, Metabolic disease |
|  | Drug-Related Side Effects and Adverse Reactions |  |
|  | Dyslipidemias | Metabolic disease |
|  | Encephalomyelitis, Autoimmune, Experimental | Immune system disease, Nervous system disease |
|  | Fatty Liver | Digestive system disease |
|  | Fatty Liver, Alcoholic | Digestive system disease, Substance-related disorder |
|  | Hepatomegaly | Digestive system disease, Pathology (anatomical condition) |
|  | Hyperlipidemias | Metabolic disease |
|  | Hyperlipoproteinemias | Metabolic disease |
|  | Hypertension | Cardiovascular disease |
|  | Hypertriglyceridemia | Metabolic disease |
|  | Hypoglycemia | Metabolic disease |
|  | Inflammation | Pathology (process) |
|  | Insulin Resistance | Metabolic disease |
|  | Kidney Diseases | Urogenital disease (female), Urogenital disease (male) |
|  | Kidney Failure, Chronic | Pathology (process), Urogenital disease (female), Urogenital disease (male) |
|  | Kidney Tubular Necrosis, Acute | Urogenital disease (female), Urogenital disease (male) |
|  | Liver Neoplasms | Cancer, Digestive system disease |
|  | Mammary Neoplasms, Animal | Animal disease, Cancer |
|  | Mammary Neoplasms, Experimental | Cancer |
|  | Muscular Diseases | Musculoskeletal disease, Nervous system disease |
|  | Myocardial Reperfusion Injury | Cardiovascular disease, Pathology (process) |
|  | Neoplasms | Cancer |
|  | Non-alcoholic Fatty Liver Disease | Digestive system disease |
|  | Obesity | Nutrition disorder, Signs and symptoms |
|  | Prostatic Neoplasms | Cancer, Urogenital disease (male) |
|  | Proteinuria | Signs and symptoms, Urogenital disease (female), Urogenital disease (male) |
|  | Reperfusion Injury | Cardiovascular disease, Pathology (process) |
| FABP3 | Muscular Diseases | Musculoskeletal disease, Nervous system disease |
|  | Necrosis | Pathology (process) |
| FABP5 | Carcinoma, Hepatocellular | Cancer, Digestive system disease |
|  | Myocardial Ischemia | Cardiovascular disease |
| PPARD | Cardiomegaly | Cardiovascular disease, Pathology (anatomical condition) |
|  | Cardiomyopathies | Cardiovascular disease |
|  | Fatty Liver | Digestive system disease |
|  | Liver Diseases, Alcoholic | Digestive system disease, Substance-related disorder |
|  | Melanoma | Cancer |
|  | Non-alcoholic Fatty Liver Disease | Digestive system disease |
|  | Obesity | Nutrition disorder, Signs and symptoms |
|  | Peripheral Nervous system diseases | Nervous system disease |
|  | Skin Neoplasms | Cancer, Skin disease |
|  | Weight Gain | Signs and symptoms |
| FABP1 | Kidney Failure, Chronic | Pathology (process), Urogenital disease (female), Urogenital disease (male) |
|  | Kidney Tubular Necrosis, Acute | Urogenital disease (female), Urogenital disease (male) |
|  | Lipidoses | Genetic disease (inborn), Metabolic disease |
|  | Liver Failure, Acute | Digestive system disease |
| RORA | Autistic Disorder | Mental disorder |
|  | Stomach Neoplasms | Cancer, Digestive system disease |
| HMOX1 | Acute Kidney Injury | Urogenital disease (female), Urogenital disease (male) |
|  | Adrenoleukodystrophy | Endocrine system disease, Genetic disease (inborn), Metabolic disease, Nervous system disease |
|  | Alzheimer Disease | Mental disorder, Nervous system disease |
|  | Anemia, Hemolytic | Blood disease |
|  | Asthma | Immune system disease, Respiratory tract disease |
|  | Blood Coagulation Disorders | Blood disease |
|  | Breast Neoplasms | Cancer, Skin disease |
|  | Cardiomegaly | Cardiovascular disease, Pathology (anatomical condition) |
|  | Carotid Artery Diseases | Cardiovascular disease, Nervous system disease |
|  | Cerebral Hemorrhage | Cardiovascular disease, Nervous system disease, Pathology (process) |
|  | Chemical and Drug Induced Liver Injury | Digestive system disease |
|  | Colitis | Digestive system disease |
|  | Coronary Artery Disease | Cardiovascular disease |
|  | Dermatitis, Contact | Skin disease |
|  | Diabetes Mellitus, Experimental | Endocrine system disease, Metabolic disease |
|  | Diabetes Mellitus, Type 2 | Endocrine system disease, Metabolic disease |
|  | Diabetic Angiopathies | Cardiovascular disease, Endocrine system disease |
|  | Drug-Related Side Effects and Adverse Reactions |  |
|  | Fibrosis | Pathology (process) |
|  | Gastroparesis | Digestive system disease, Signs and symptoms |
|  | Growth Disorders | Pathology (process) |
|  | Heart Failure | Cardiovascular disease |
|  | Heme Oxygenase 1 Deficiency | Blood disease, Metabolic disease, Pathology (process) |
|  | Hemolysis | Pathology (process) |
|  | Hepatitis | Digestive system disease |
|  | Hepatorenal Syndrome | Digestive system disease, Urogenital disease (female), Urogenital disease (male) |
|  | Hyperinsulinism | Metabolic disease |
|  | Hyperplasia | Pathology (process) |
|  | Hypertension | Cardiovascular disease |
|  | Inflammation | Pathology (process) |
|  | Insulin Resistance | Metabolic disease |
|  | Iron Metabolism Disorders | Metabolic disease |
|  | Ischemia | Pathology (process) |
|  | Kidney Failure, Chronic | Pathology (process), Urogenital disease (female), Urogenital disease (male) |
|  | Learning Disabilities | Mental disorder, Nervous system disease, Signs and symptoms |
|  | Leishmaniasis, Visceral | Parasitic disease |
|  | Liver Cirrhosis, Experimental | Digestive system disease, Pathology (process) |
|  | Liver Diseases | Digestive system disease |
|  | Liver Neoplasms | Cancer, Digestive system disease |
|  | Lung Injury | Respiratory tract disease, Wounds and injuries |
|  | Lung Neoplasms | Cancer, Respiratory tract disease |
|  | Mammary Neoplasms, Experimental | Cancer |
|  | Mastocytosis, Systemic | Cancer, Immune system disease |
|  | Myocardial Ischemia | Cardiovascular disease |
|  | Neoplasm Invasiveness | Cancer, Pathology (process) |
|  | Neurodegenerative Diseases | Nervous system disease |
|  | Obesity | Nutrition disorder, Signs and symptoms |
|  | Pancreatic Diseases | Digestive system disease |
|  | Parkinson Disease | Nervous system disease |
|  | Pneumonia | Respiratory tract disease |
|  | Pre-Eclampsia | Pregnancy complication |
|  | Prostatic Neoplasms | Cancer, Urogenital disease (male) |
|  | Pulmonary Disease, Chronic Obstructive | Pathology (process), Respiratory tract disease |
|  | Pulmonary Emphysema | Pathology (process), Respiratory tract disease |
|  | Pulmonary Fibrosis | Pathology (process), Respiratory tract disease |
|  | Reperfusion Injury | Cardiovascular disease, Pathology (process) |
|  | Retinal Diseases | Eye disease |
|  | Spinocerebellar Ataxia 17 | Genetic disease (inborn), Nervous system disease |
|  | Status Epilepticus | Nervous system disease, Signs and symptoms |
|  | Stomach Neoplasms | Cancer, Digestive system disease |
|  | Thrombosis | Cardiovascular disease |
|  | Vascular System Injuries | Cardiovascular disease, Wounds and injuries |
|  | Wounds and Injuries | Wounds and injuries |
| HMGCR | Autoimmune Diseases | Immune system disease |
|  | Carcinoma, Hepatocellular | Cancer, Digestive system disease |
|  | Gallstones | Digestive system disease, Pathology (anatomical condition) |
|  | Hypercholesterolemia | Metabolic disease |
|  | Hyperlipoproteinemia Type II | Genetic disease (inborn), Metabolic disease |
|  | Kidney Failure, Chronic | Pathology (process), Urogenital disease (female), Urogenital disease (male) |
|  | Liver Cirrhosis, Experimental | Digestive system disease, Pathology (process) |
|  | Mevalonate Kinase Deficiency | Blood disease, Genetic disease (inborn), Immune system disease, Metabolic disease, Nervous system disease |
|  | Muscular Diseases | Musculoskeletal disease, Nervous system disease |
|  | Q Fever | Bacterial infection or mycosis |
| CD81 | Common Variable Immunodeficiency | Immune system disease |
|  | Hepatitis C | Digestive system disease, Viral disease |
| PGR | Adenocarcinoma | Cancer |
|  | Breast Neoplasms | Cancer, Skin disease |
|  | Carcinoma | Cancer |
|  | Endometrial Neoplasms | Cancer, Urogenital disease (female) |
|  | Endometriosis | Urogenital disease (female) |
|  | Mammary Neoplasms, Animal | Animal disease, Cancer |
|  | Mammary Neoplasms, Experimental | Cancer |
|  | Meningioma | Cancer, Nervous system disease |
|  | Mesothelioma | Cancer |
|  | Progesterone Resistance | Urogenital disease (female) |
|  | Recurrence | Pathology (process) |
| G6PD | Acute Kidney Injury | Urogenital disease (female), Urogenital disease (male) |
|  | Anemia, Hemolytic | Blood disease |
|  | Anemia, Hemolytic, Congenital Nonspherocytic | Blood disease, Genetic disease (inborn) |
|  | ANEMIA, NONSPHEROCYTIC HEMOLYTIC, DUE TO G6PD DEFICIENCY | Blood disease, Genetic disease (inborn), Metabolic disease |
|  | Dermatitis, Contact | Skin disease |
|  | Favism | Blood disease, Genetic disease (inborn) |
|  | Glucosephosphate Dehydrogenase Deficiency | Blood disease, Genetic disease (inborn), Metabolic disease |
|  | Granulomatous Disease, Chronic | Blood disease, Genetic disease (inborn), Immune system disease, Pathology (process) |
|  | Hemolysis | Pathology (process) |
|  | Malaria | Parasitic disease |
|  | Phagocyte Bactericidal Dysfunction | Blood disease, Immune system disease |
| SCD | Carcinoma | Cancer |
|  | Carcinoma, Hepatocellular | Cancer, Digestive system disease |
|  | Graves Ophthalmopathy | Endocrine system disease, Eye disease, Genetic disease (inborn), Immune system disease |
|  | Lipidoses | Genetic disease (inborn), Metabolic disease |
|  | Liver Neoplasms | Cancer, Digestive system disease |
|  | Mammary Neoplasms, Animal | Animal disease, Cancer |
|  | Mammary Neoplasms, Experimental | Cancer |
| ADRA2C | Cardiomegaly | Cardiovascular disease, Pathology (anatomical condition) |
|  | Fibrosis | Pathology (process) |
|  | Heart Failure | Cardiovascular disease |
| HSD11B1 | Arthritis, Rheumatoid | Connective tissue disease, Immune system disease, Musculoskeletal disease |
|  | Cortisone reductase deficiency | Congenital abnormality, Endocrine system disease, Genetic disease (inborn), Metabolic disease, Signs and symptoms, Skin disease, Urogenital disease (female), Urogenital disease (male) |
|  | Dermatitis, Allergic Contact | Immune system disease, Skin disease |
|  | Diabetes Mellitus, Experimental | Endocrine system disease, Metabolic disease |
|  | Hyperglycemia | Metabolic disease |
|  | Hypertension | Cardiovascular disease |
|  | Insulin Resistance | Metabolic disease |
|  | Obesity | Nutrition disorder, Signs and symptoms |
|  | Obesity, Abdominal | Nutrition disorder, Signs and symptoms |
| SLC6A3 | Alcoholism | Mental disorder, Substance-related disorder |
|  | Alcohol Withdrawal Delirium | Mental disorder, Nervous system disease, Substance-related disorder |
|  | Alcohol Withdrawal Seizures | Nervous system disease, Signs and symptoms, Substance-related disorder |
|  | Amphetamine-Related Disorders | Mental disorder, Substance-related disorder |
|  | Anxiety Disorders | Mental disorder |
|  | Attention Deficit Disorder with Hyperactivity | Mental disorder |
|  | Cocaine-Related Disorders | Mental disorder, Substance-related disorder |
|  | Dementia | Mental disorder, Nervous system disease |
|  | Genetic Predisposition to Disease | Pathology (process) |
|  | Hyperkinesis | Nervous system disease, Signs and symptoms |
|  | Memory Disorders | Nervous system disease, Signs and symptoms |
|  | Micronuclei, Chromosome-Defective | Pathology (process) |
|  | Nerve Degeneration | Pathology (process) |
|  | Nervous system diseases | Nervous system disease |
|  | Paranoid Disorders | Mental disorder |
|  | Parkinson Disease | Nervous system disease |
|  | Parkinsonism-Dystonia, Infantile | Nervous system disease |
|  | Psychoses, Substance-Induced | Mental disorder, Substance-related disorder |
|  | Schizophrenia | Mental disorder |
|  | Sleep Wake Disorders | Mental disorder, Nervous system disease, Signs and symptoms |
|  | Tic Disorders | Mental disorder, Nervous system disease |
|  | TOBACCO ADDICTION, SUSCEPTIBILITY TO | Mental disorder, Substance-related disorder |
| RORC | IMMUNODEFICIENCY 42 | Immune system disease |
|  | Inflammation | Pathology (process) |
|  | Sleep Disorders, Circadian Rhythm | Mental disorder, Nervous system disease, Occupational disease |
| HSD17B2 | Endometrial Neoplasms | Cancer, Urogenital disease (female) |
|  | Endometriosis | Urogenital disease (female) |
|  | Liver Cirrhosis, Experimental | Digestive system disease, Pathology (process) |
| TYR | Albinism, Oculocutaneous | Eye disease, Genetic disease (inborn), Metabolic disease, Skin disease |
|  | Carcinoma, Basal Cell | Cancer |
|  | Melanoma | Cancer |
|  | Oculocutaneous albinism type 1 | Eye disease, Genetic disease (inborn), Metabolic disease, Skin disease |
|  | Oculocutaneous albinism type 1B | Eye disease, Genetic disease (inborn), Metabolic disease, Skin disease |
| TYR | Skin Neoplasms | Cancer, Skin disease |
| DAO | Autistic Disorder | Mental disorder |
|  | Liver Cirrhosis, Experimental | Digestive system disease, Pathology (process) |
|  | Schizophrenia | Mental disorder |
| ERN1 | Chemical and Drug Induced Liver Injury | Digestive system disease |
|  | Encephalomyelitis, Autoimmune, Experimental | Immune system disease, Nervous system disease |
|  | Epilepsy | Nervous system disease |
|  | Liver Cirrhosis, Experimental | Digestive system disease, Pathology (process) |
|  | Multiple Sclerosis | Immune system disease, Nervous system disease |
| MIF | Anxiety Disorders | Mental disorder |
|  | Arthritis, Rheumatoid | Connective tissue disease, Immune system disease, Musculoskeletal disease |
|  | Autism Spectrum Disorder | Mental disorder |
|  | Autistic Disorder | Mental disorder |
|  | Breast Neoplasms | Cancer, Skin disease |
|  | Carcinoma | Cancer |
|  | Colitis | Digestive system disease |
|  | Depressive Disorder | Mental disorder |
|  | Disease Progression | Pathology (process) |
|  | Inflammation | Pathology (process) |
|  | Keloid | Connective tissue disease, Pathology (process) |
|  | Mammary Neoplasms, Animal | Animal disease, Cancer |
|  | Mammary Neoplasms, Experimental | Cancer |
|  | Memory Disorders | Nervous system disease, Signs and symptoms |
|  | Mesothelioma, Malignant | Cancer, Respiratory tract disease |
|  | Prostatic Neoplasms | Cancer, Urogenital disease (male) |
|  | Rheumatoid Arthritis, Systemic Juvenile | Connective tissue disease, Immune system disease, Musculoskeletal disease |
|  | Sepsis | Pathology (process) |
|  | Stevens-Johnson Syndrome | Immune system disease, Mouth disease, Skin disease |
| GSR | Amyotrophic Lateral Sclerosis | Metabolic disease, Nervous system disease |
|  | Anemia | Blood disease |
|  | Anemia, Hemolytic | Blood disease |
|  | Chemical and Drug Induced Liver Injury | Digestive system disease |
|  | Dermatitis, Contact | Skin disease |
|  | Diabetes Mellitus, Experimental | Endocrine system disease, Metabolic disease |
|  | Edema | Signs and symptoms |
|  | Hyperthyroidism | Endocrine system disease |
|  | Hypoglycemia | Metabolic disease |
|  | Neurodegenerative Diseases | Nervous system disease |
|  | Parkinsonian Disorders | Nervous system disease |
|  | Protein Deficiency | Nutrition disorder |
|  | Ureteral Calculi | Pathology (anatomical condition), Urogenital disease (female), Urogenital disease (male) |
| CYP11B1 | Congenital adrenal hyperplasia due to 11-Beta-hydroxylase deficiency | Congenital abnormality, Endocrine system disease, Genetic disease (inborn), Metabolic disease, Urogenital disease (female), Urogenital disease (male) |
|  | Glucocorticoid-Remediable Aldosteronism | Endocrine system disease |
|  | Hyperaldosteronism | Endocrine system disease |
| CYP11B2 | 18-Hydroxylase deficiency | Endocrine system disease |
|  | Glucocorticoid-Remediable Aldosteronism | Endocrine system disease |
|  | Hyperaldosteronism | Endocrine system disease |
|  | Hypoaldosteronism | Endocrine system disease |
|  | Prostatic Neoplasms | Cancer, Urogenital disease (male) |
| FAAH | Huntington Disease | Genetic disease (inborn), Mental disorder, Nervous system disease |
|  | Obesity | Nutrition disorder, Signs and symptoms |
|  | Pregnancy Complications | Pregnancy complication |
|  | Seizures | Nervous system disease, Signs and symptoms |
|  | Substance-Related Disorders | Mental disorder, Substance-related disorder |
|  | Substance Withdrawal Syndrome | Mental disorder, Substance-related disorder |
| TRPV1 | Atherosclerosis | Cardiovascular disease |
|  | Cough | Respiratory tract disease, Signs and symptoms |
|  | Endometrial Neoplasms | Cancer, Urogenital disease (female) |
|  | Epilepsy, Temporal Lobe | Nervous system disease |
|  | Esophageal Diseases | Digestive system disease |
|  | Esophagitis | Digestive system disease |
|  | Heartburn | Signs and symptoms |
|  | Hyperalgesia | Nervous system disease, Signs and symptoms |
|  | Inflammation | Pathology (process) |
|  | Liver Cirrhosis, Experimental | Digestive system disease, Pathology (process) |
|  | Neoplasms | Cancer |
|  | Obesity | Nutrition disorder, Signs and symptoms |
|  | Pain | Signs and symptoms |
|  | Pancreatitis | Digestive system disease |
|  | Sneezing | Signs and symptoms |
|  | Urinary Bladder Neoplasms | Cancer, Urogenital disease (female), Urogenital disease (male) |
| ADH1A | Liver Failure, Acute | Digestive system disease |
|  | Non-alcoholic Fatty Liver Disease | Digestive system disease |
| ADH1B | Alcoholism | Mental disorder, Substance-related disorder |
|  | Cerebral Infarction | Cardiovascular disease, Nervous system disease, Pathology (process) |
|  | Colorectal Neoplasms | Cancer, Digestive system disease |
|  | Esophageal Neoplasms | Cancer, Digestive system disease |
|  | Esophageal Squamous Cell Carcinoma | Cancer, Digestive system disease |
|  | Genetic Predisposition to Disease | Pathology (process) |
|  | Laryngeal Neoplasms | Cancer, Ear-nose-throat disease, Respiratory tract disease |
|  | Liver Failure, Acute | Digestive system disease |
|  | Mouth Neoplasms | Cancer, Mouth disease |
|  | Non-alcoholic Fatty Liver Disease | Digestive system disease |
|  | Obesity | Nutrition disorder, Signs and symptoms |
|  | Pharyngeal Neoplasms | Cancer, Ear-nose-throat disease, Mouth disease |
| ADH7 | Esophageal Neoplasms | Cancer, Digestive system disease |
|  | Laryngeal Neoplasms | Cancer, Ear-nose-throat disease, Respiratory tract disease |
|  | Mouth Neoplasms | Cancer, Mouth disease |
|  | Pharyngeal Neoplasms | Cancer, Ear-nose-throat disease, Mouth disease |
| SRD5A1 | Endometriosis | Urogenital disease (female) |
|  | Liver Cirrhosis, Experimental | Digestive system disease, Pathology (process) |
|  | Polycystic Ovary Syndrome | Cancer, Endocrine system disease, Urogenital disease (female) |
|  | Prostatic Neoplasms | Cancer, Urogenital disease (male) |
| TRPA1 | Asthma | Immune system disease, Respiratory tract disease |
|  | Bronchial Hyperreactivity | Respiratory tract disease |
|  | Cough | Respiratory tract disease, Signs and symptoms |
|  | EPISODIC PAIN SYNDROME, FAMILIAL, 1 | Signs and symptoms |
|  | Hyperalgesia | Nervous system disease, Signs and symptoms |
|  | Inflammation | Pathology (process) |
|  | Pain | Signs and symptoms |
|  | Sneezing | Signs and symptoms |
|  | Urinary Bladder, Overactive | Signs and symptoms, Urogenital disease (female), Urogenital disease (male) |
| MAOB | Alzheimer Disease | Mental disorder, Nervous system disease |
|  | Anemia, Megaloblastic | Blood disease |
|  | Autistic Disorder | Mental disorder |
|  | Colonic Neoplasms | Cancer, Digestive system disease |
|  | Endometriosis | Urogenital disease (female) |
|  | Hepatic Encephalopathy | Digestive system disease, Metabolic disease, Nervous system disease |
|  | Huntington Disease | Genetic disease (inborn), Mental disorder, Nervous system disease |
|  | Hypotension | Cardiovascular disease |
|  | Parkinson Disease | Nervous system disease |
|  | Pheochromocytoma | Cancer |
|  | Plaque, Amyloid | Pathology (anatomical condition) |
|  | Schizophrenia | Mental disorder |
| HLCS | Holocarboxylase Synthetase Deficiency | Genetic disease (inborn), Metabolic disease |
| MAOA | Antisocial Personality Disorder | Mental disorder |
|  | Autistic Disorder | Mental disorder |
|  | Brunner Syndrome | Genetic disease (inborn), Mental disorder, Nervous system disease, Signs and symptoms |
|  | Cognition Disorders | Mental disorder |
|  | Endometriosis | Urogenital disease (female) |
|  | End Stage Liver Disease | Digestive system disease |
|  | Fetal Nutrition Disorders | Fetal disease, Nutrition disorder, Pregnancy complication |
|  | Hepatic Encephalopathy | Digestive system disease, Metabolic disease, Nervous system disease |
|  | Huntington Disease | Genetic disease (inborn), Mental disorder, Nervous system disease |
|  | Hypotension | Cardiovascular disease |
|  | Mental disorders | Mental disorder |
|  | Mental Retardation, X-Linked | Genetic disease (inborn), Nervous system disease |
|  | Muscular Diseases | Musculoskeletal disease, Nervous system disease |
|  | Parkinson Disease | Nervous system disease |
|  | Pheochromocytoma | Cancer |
|  | Prenatal Exposure Delayed Effects | Pregnancy complication |
| SRD5A2 | Alopecia | Pathology (anatomical condition), Skin disease |
|  | Endometrial Neoplasms | Cancer, Urogenital disease (female) |
|  | Endometriosis | Urogenital disease (female) |
|  | Prostatic Hyperplasia | Urogenital disease (male) |
|  | Prostatic Neoplasms | Cancer, Urogenital disease (male) |
|  | Pseudovaginal Perineoscrotal Hypospadias | Congenital abnormality, Endocrine system disease, Genetic disease (inborn), Metabolic disease, Urogenital disease (female), Urogenital disease (male) |
| ALDH1A1 | Carcinoma, Renal Cell | Cancer, Urogenital disease (female), Urogenital disease (male) |
|  | Cell Transformation, Neoplastic | Cancer, Pathology (process) |
|  | Creutzfeldt-Jakob Syndrome | Mental disorder, Nervous system disease |
|  | Liver Cirrhosis, Experimental | Digestive system disease, Pathology (process) |
|  | Liver Diseases | Digestive system disease |
|  | Melanoma | Cancer |
|  | Non-alcoholic Fatty Liver Disease | Digestive system disease |
|  | Parkinsonian Disorders | Nervous system disease |
| PTGS1 | Arthritis, Rheumatoid | Connective tissue disease, Immune system disease, Musculoskeletal disease |
|  | Asthma, Occupational | Immune system disease, Occupational disease, Respiratory tract disease |
|  | Breast Neoplasms | Cancer, Skin disease |
|  | Bronchial Hyperreactivity | Respiratory tract disease |
|  | Carcinoma | Cancer |
|  | Drug Hypersensitivity | Immune system disease |
|  | Esophageal Neoplasms | Cancer, Digestive system disease |
|  | Flushing | Signs and symptoms |
|  | Heart Failure | Cardiovascular disease |
|  | Hyperalgesia | Nervous system disease, Signs and symptoms |
|  | Intestinal Polyps | Pathology (anatomical condition) |
|  | Kidney Failure, Chronic | Pathology (process), Urogenital disease (female), Urogenital disease (male) |
|  | Liver Cirrhosis, Experimental | Digestive system disease, Pathology (process) |
|  | Ovarian Cysts | Cancer, Endocrine system disease, Urogenital disease (female) |
|  | Reperfusion Injury | Cardiovascular disease, Pathology (process) |
|  | Sleep Deprivation | Mental disorder, Nervous system disease, Signs and symptoms |
|  | Stomach Ulcer | Digestive system disease |
| DRD4 | Amphetamine-Related Disorders | Mental disorder, Substance-related disorder |
|  | Anxiety, Separation | Mental disorder |
|  | Attention Deficit and Disruptive Behavior Disorders | Mental disorder |
|  | Attention Deficit Disorder with Hyperactivity | Mental disorder |
|  | Child Development Disorders, Pervasive | Mental disorder |
|  | Schizophrenia | Mental disorder |
|  | Tic Disorders | Mental disorder, Nervous system disease |
| CTSK | Glioblastoma | Cancer |
|  | Osteoarthritis | Musculoskeletal disease |
|  | Pycnodysostosis | Genetic disease (inborn), Metabolic disease, Musculoskeletal disease |
| CTSL | Coronavirus Infections | Viral disease |
|  | Meningioma | Cancer, Nervous system disease |
|  | Proteinuria | Signs and symptoms, Urogenital disease (female), Urogenital disease (male) |
|  | Recurrence | Pathology (process) |
|  | Severe Acute Respiratory Syndrome | Respiratory tract disease, Viral disease |
|  | Stomach Neoplasms | Cancer, Digestive system disease |
| CTSB | Carcinoma, Renal Cell | Cancer, Urogenital disease (female), Urogenital disease (male) |
|  | Disease Progression | Pathology (process) |
|  | Glioblastoma | Cancer |
|  | Inflammatory Breast Neoplasms | Cancer, Skin disease |
|  | Keratolytic winter erythema | Genetic disease (inborn), Signs and symptoms, Skin disease |
|  | Muscular Diseases | Musculoskeletal disease, Nervous system disease |
|  | Necrosis | Pathology (process) |
|  | Neoplasm Invasiveness | Cancer, Pathology (process) |
|  | Prostatic Neoplasms | Cancer, Urogenital disease (male) |
|  | Proteinuria | Signs and symptoms, Urogenital disease (female), Urogenital disease (male) |
| PPARG | Acute Kidney Injury | Urogenital disease (female), Urogenital disease (male) |
|  | Acute Lung Injury | Respiratory tract disease |
|  | Adenocarcinoma | Cancer |
|  | Alzheimer Disease | Mental disorder, Nervous system disease |
|  | Antiphospholipid Syndrome | Immune system disease |
|  | Atherosclerosis | Cardiovascular disease |
|  | Barrett Esophagus | Cancer, Digestive system disease |
|  | Carcinoma, Hepatocellular | Cancer, Digestive system disease |
|  | Carotid Intimal Medial Thickness 1 | Cardiovascular disease |
|  | Chronobiology Disorders | Nervous system disease |
|  | Colonic Neoplasms | Cancer, Digestive system disease |
|  | Colorectal Neoplasms | Cancer, Digestive system disease |
|  | Crohn Disease | Digestive system disease |
|  | Diabetes Mellitus | Endocrine system disease, Metabolic disease |
|  | Diabetes Mellitus, Experimental | Endocrine system disease, Metabolic disease |
|  | Diabetes Mellitus, Type 2 | Endocrine system disease, Metabolic disease |
|  | Diabetic Nephropathies | Endocrine system disease, Urogenital disease (female), Urogenital disease (male) |
|  | Dyslipidemias | Metabolic disease |
|  | Glomerulonephritis | Urogenital disease (female), Urogenital disease (male) |
|  | Heart Failure | Cardiovascular disease |
|  | Hypertension | Cardiovascular disease |
|  | Inflammation | Pathology (process) |
|  | Insulin Resistance | Metabolic disease |
|  | Ischemia | Pathology (process) |
|  | Leukemia, Promyelocytic, Acute | Cancer |
|  | Leukostasis | Blood disease |
|  | Lipidoses | Genetic disease (inborn), Metabolic disease |
|  | Lipodystrophy, Familial Partial | Genetic disease (inborn), Metabolic disease, Skin disease |
|  | Liver Neoplasms | Cancer, Digestive system disease |
|  | Lymphoma, T-Cell | Cancer, Immune system disease, Lymphatic disease |
|  | Melanoma | Cancer |
|  | Metabolic Diseases | Metabolic disease |
|  | Nerve Degeneration | Pathology (process) |
|  | Non-alcoholic Fatty Liver Disease | Digestive system disease |
|  | Obesity | Nutrition disorder, Signs and symptoms |
|  | Osteoarthritis | Musculoskeletal disease |
|  | Pancreatic Neoplasms | Cancer, Digestive system disease, Endocrine system disease |
|  | Pituitary ACTH Hypersecretion | Endocrine system disease, Nervous system disease |
|  | Polycystic Kidney, Autosomal Dominant | Congenital abnormality, Genetic disease (inborn), Urogenital disease (female), Urogenital disease (male) |
|  | Pre-Eclampsia | Pregnancy complication |
|  | Psoriasis | Skin disease |
|  | Renal Insufficiency | Urogenital disease (female), Urogenital disease (male) |
|  | Reperfusion Injury | Cardiovascular disease, Pathology (process) |
|  | Stomach Neoplasms | Cancer, Digestive system disease |
|  | Subarachnoid Hemorrhage | Cardiovascular disease, Nervous system disease, Pathology (process) |
|  | Thyroid Cancer, follicular | Cancer |
|  | Thyroid Neoplasms | Cancer, Endocrine system disease |
| ABCG2 | Adenoma | Cancer |
|  | Arthritis, Rheumatoid | Connective tissue disease, Immune system disease, Musculoskeletal disease |
|  | Breast Neoplasms | Cancer, Skin disease |
|  | Cardiomyopathies | Cardiovascular disease |
|  | Colorectal Neoplasms | Cancer, Digestive system disease |
|  | Diarrhea | Signs and symptoms |
|  | Drug-Related Side Effects and Adverse Reactions |  |
|  | Gout | Genetic disease (inborn), Metabolic disease, Musculoskeletal disease |
|  | Hyperuricemia | Pathology (process) |
|  | Liver Cirrhosis, Biliary | Digestive system disease, Pathology (process) |
|  | Lymphoma, Large B-Cell, Diffuse | Cancer, Immune system disease, Lymphatic disease |
|  | Mammary Neoplasms, Experimental | Cancer |
|  | Nervous system diseases | Nervous system disease |
|  | Precursor T-Cell Lymphoblastic Leukemia-Lymphoma | Cancer, Immune system disease, Lymphatic disease |
|  | Squamous Cell Carcinoma of Head and Neck | Cancer |
|  | URIC ACID CONCENTRATION, SERUM, QUANTITATIVE TRAIT LOCUS 1 | Genetic disease (inborn), Metabolic disease, Musculoskeletal disease, Pathology (process) |
| MTNR1A | Autism Spectrum Disorder | Mental disorder |
|  | Retinal Diseases | Eye disease |
| MTNR1B | Autism Spectrum Disorder | Mental disorder |
|  | Diabetes Mellitus, Type 2 | Endocrine system disease, Metabolic disease |
|  | Polycystic Ovary Syndrome | Cancer, Endocrine system disease, Urogenital disease (female) |
| ADH1C | Alcoholism | Mental disorder, Substance-related disorder |
|  | Genetic Predisposition to Disease | Pathology (process) |
|  | Mouth Neoplasms | Cancer, Mouth disease |
|  | PARKINSON DISEASE, LATE-ONSET | Nervous system disease |
| PARP1 | Alopecia | Pathology (anatomical condition), Skin disease |
|  | Anemia | Blood disease |
|  | Aortic Aneurysm, Abdominal | Cardiovascular disease |
|  | Asthma | Immune system disease, Respiratory tract disease |
|  | Atherosclerosis | Cardiovascular disease |
|  | Brain Injuries | Nervous system disease, Wounds and injuries |
|  | Brain Injuries, Traumatic | Nervous system disease, Wounds and injuries |
|  | Brain Ischemia | Cardiovascular disease, Nervous system disease |
|  | Breast Neoplasms | Cancer, Skin disease |
|  | Carcinoma, Hepatocellular | Cancer, Digestive system disease |
|  | Cardiomyopathies | Cardiovascular disease |
|  | Cell Transformation, Neoplastic | Cancer, Pathology (process) |
|  | Ceroid lipofuscinosis, neuronal 1, infantile | Genetic disease (inborn), Metabolic disease, Nervous system disease |
|  | Colorectal Neoplasms | Cancer, Digestive system disease |
|  | Dermatitis | Skin disease |
|  | Hepatitis | Digestive system disease |
|  | Inflammation | Pathology (process) |
|  | Intellectual Disability | Mental disorder, Nervous system disease, Signs and symptoms |
|  | Kidney Diseases | Urogenital disease (female), Urogenital disease (male) |
|  | Kyphosis | Musculoskeletal disease |
|  | Lung Injury | Respiratory tract disease, Wounds and injuries |
|  | Melanoma | Cancer |
|  | Mesothelioma, Malignant | Cancer, Respiratory tract disease |
|  | Multiple Myeloma | Blood disease, Cancer, Cardiovascular disease, Immune system disease |
|  | Obesity | Nutrition disorder, Signs and symptoms |
|  | Pancreatitis | Digestive system disease |
|  | Paralysis | Nervous system disease, Signs and symptoms |
|  | Parkinsonian Disorders | Nervous system disease |
|  | Plaque, Atherosclerotic | Pathology (anatomical condition) |
|  | Pneumonia | Respiratory tract disease |
|  | Prostatic Neoplasms | Cancer, Urogenital disease (male) |
|  | Pulmonary Edema | Respiratory tract disease |
|  | Pulmonary Fibrosis | Pathology (process), Respiratory tract disease |
|  | Reperfusion Injury | Cardiovascular disease, Pathology (process) |
|  | Spinal Cord Injuries | Nervous system disease, Wounds and injuries |
|  | Spinal Cord Ischemia | Cardiovascular disease, Nervous system disease |
|  | Stevens-Johnson Syndrome | Immune system disease, Mouth disease, Skin disease |
|  | Vitamin A Deficiency | Nutrition disorder |
| CTSD | Amyotrophic lateral sclerosis 1 | Metabolic disease, Nervous system disease |
|  | Arthritis, Rheumatoid | Connective tissue disease, Immune system disease, Musculoskeletal disease |
|  | Carcinoma, Hepatocellular | Cancer, Digestive system disease |
|  | Carcinoma, Renal Cell | Cancer, Urogenital disease (female), Urogenital disease (male) |
|  | Ceroid Lipofuscinosis, Neuronal, 10 | Genetic disease (inborn), Metabolic disease, Nervous system disease |
|  | Diabetic Nephropathies | Endocrine system disease, Urogenital disease (female), Urogenital disease (male) |
|  | Kidney Diseases | Urogenital disease (female), Urogenital disease (male) |
|  | Neoplasm Invasiveness | Cancer, Pathology (process) |
|  | Osteoarthritis | Musculoskeletal disease |
|  | Prostatic Neoplasms | Cancer, Urogenital disease (male) |
|  | Weight Gain | Signs and symptoms |
| 3 | Craniofacial Abnormalities | Congenital abnormality, Musculoskeletal disease |
|  | Liver Cirrhosis, Experimental | Digestive system disease, Pathology (process) |
|  | Metaphyseal chondrodysplasia Spahr type | Genetic disease (inborn), Musculoskeletal disease |
|  | Prostatic Neoplasms | Cancer, Urogenital disease (male) |
|  | Spondyloepimetaphyseal Dysplasia, Missouri Type | Genetic disease (inborn), Musculoskeletal disease |
| MMP1 | Aneurysm | Cardiovascular disease |
|  | Asthma | Immune system disease, Respiratory tract disease |
|  | Atherosclerosis | Cardiovascular disease |
|  | Breast Neoplasms | Cancer, Skin disease |
|  | Carcinoma, Non-Small-Cell Lung | Cancer, Respiratory tract disease |
|  | Colorectal Neoplasms | Cancer, Digestive system disease |
|  | Epidermolysis Bullosa Dystrophica | Congenital abnormality, Connective tissue disease, Genetic disease (inborn), Skin disease |
|  | Liver Cirrhosis, Experimental | Digestive system disease, Pathology (process) |
|  | Lung Neoplasms | Cancer, Respiratory tract disease |
|  | Neoplasm Invasiveness | Cancer, Pathology (process) |
|  | Neoplasm Metastasis | Cancer, Pathology (process) |
|  | Non-alcoholic Fatty Liver Disease | Digestive system disease |
|  | Ocular Hypertension | Eye disease |
|  | Oral Submucous Fibrosis | Mouth disease |
|  | Pulmonary Disease, Chronic Obstructive | Pathology (process), Respiratory tract disease |
| HDAC6 | Chondrodysplasia with platyspondyly, distinctive brachydactyly, hydrocephaly, and microphthalmia | Congenital abnormality, Eye disease, Genetic disease (inborn), Musculoskeletal disease, Nervous system disease |
|  | Disease Progression | Pathology (process) |
|  | Melanoma | Cancer |
|  | Neoplasms, Experimental | Cancer |
|  | Osteosarcoma | Cancer |
|  | Ovarian Neoplasms | Cancer, Endocrine system disease, Urogenital disease (female) |
|  | Prostatic Neoplasms | Cancer, Urogenital disease (male) |
| HDAC1 | Arthritis, Rheumatoid | Connective tissue disease, Immune system disease, Musculoskeletal disease |
|  | Carcinoma, Squamous Cell | Cancer |
|  | Endometriosis | Urogenital disease (female) |
|  | Familial Primary Pulmonary Hypertension | Respiratory tract disease |
|  | Heart Diseases | Cardiovascular disease |
|  | Myelodysplastic Syndromes | Blood disease |
| CACNA1B | Peripheral Nervous system diseases | Nervous system disease |
| PSMB5 | Myocardial Ischemia | Cardiovascular disease |
| CHRM1 | Brain Ischemia | Cardiovascular disease, Nervous system disease |
|  | Epilepsy | Nervous system disease |
|  | Memory Disorders | Nervous system disease, Signs and symptoms |
|  | Seizures | Nervous system disease, Signs and symptoms |
| MMP8 | Dermatitis, Contact | Skin disease |
|  | Liver Cirrhosis, Experimental | Digestive system disease, Pathology (process) |
|  | Lung Diseases | Respiratory tract disease |
|  | Melanoma | Cancer |
|  | Tuberculosis, Pulmonary | Bacterial infection or mycosis, Respiratory tract disease |
|  | Wounds and Injuries | Wounds and injuries |
| IKBKB | Glucose Intolerance | Metabolic disease |
|  | Hyperbilirubinemia, Neonatal | Infant-newborn disease, Pathology (process) |
|  | Hypertension | Cardiovascular disease |
|  | IMMUNODEFICIENCY 15B | Immune system disease |
|  | Inflammation | Pathology (process) |
|  | Insulin Resistance | Metabolic disease |
|  | Pneumonia | Respiratory tract disease |
|  | Vascular Diseases | Cardiovascular disease |
| EPHX2 | Acute Kidney Injury | Urogenital disease (female), Urogenital disease (male) |
|  | Autism Spectrum Disorder | Mental disorder |
|  | Drug-Related Side Effects and Adverse Reactions |  |
|  | Heart Failure | Cardiovascular disease |
|  | HYPERCHOLESTEROLEMIA, FAMILIAL, 1 | Genetic disease (inborn), Metabolic disease |
|  | Myocardial Ischemia | Cardiovascular disease |
|  | Myocardial Reperfusion Injury | Cardiovascular disease, Pathology (process) |
|  | Schizophrenia | Mental disorder |
|  | Ventricular Dysfunction, Left | Cardiovascular disease |
| JAK1 | Lupus Nephritis | Connective tissue disease, Immune system disease, Urogenital disease (female), Urogenital disease (male) |
|  | Prostatic Neoplasms | Cancer, Urogenital disease (male) |
| JAK2 | Abortion, Spontaneous | Pregnancy complication |
|  | Budd-Chiari Syndrome | Cardiovascular disease, Digestive system disease |
|  | Calcinosis | Metabolic disease |
|  | Cell Transformation, Neoplastic | Cancer, Pathology (process) |
|  | Colitis, Ulcerative | Digestive system disease |
|  | Genetic Predisposition to Disease | Pathology (process) |
|  | Heart Valve Diseases | Cardiovascular disease |
|  | Inflammation | Pathology (process) |
|  | Leukemia | Cancer |
|  | Leukemia, Myeloid, Acute | Cancer |
|  | Liver Cirrhosis, Experimental | Digestive system disease, Pathology (process) |
|  | Liver Neoplasms, Experimental | Cancer, Digestive system disease |
|  | Mesenteric Vascular Occlusion | Cardiovascular disease, Digestive system disease |
|  | Myeloproliferative Disorders | Blood disease |
|  | Myocardial Ischemia | Cardiovascular disease |
|  | Non-alcoholic Fatty Liver Disease | Digestive system disease |
|  | Polycythemia | Blood disease |
|  | Polycythemia, primary familial and congenital | Blood disease |
|  | Polycythemia Vera | Blood disease, Cancer |
|  | Pregnancy Complications | Pregnancy complication |
|  | Primary Myelofibrosis | Blood disease |
|  | Prostatic Neoplasms | Cancer, Urogenital disease (male) |
|  | Thrombocythemia, Essential | Blood disease |
|  | Thrombocytosis | Blood disease |
|  | Thromboembolism | Cardiovascular disease |
|  | Uterine Cervical Neoplasms | Cancer, Urogenital disease (female) |
|  | Venous Thrombosis | Cardiovascular disease |
| ACE | Adenocarcinoma | Cancer |
|  | Albuminuria | Signs and symptoms, Urogenital disease (female), Urogenital disease (male) |
|  | Allanson Pantzar McLeod syndrome | Congenital abnormality, Urogenital disease (female), Urogenital disease (male) |
|  | Alzheimer Disease | Mental disorder, Nervous system disease |
|  | Arrhythmias, Cardiac | Cardiovascular disease, Pathology (process) |
|  | Atrial Fibrillation | Cardiovascular disease, Pathology (process) |
|  | Autistic Disorder | Mental disorder |
|  | Berylliosis | Occupational disease, Respiratory tract disease |
|  | Brain Ischemia | Cardiovascular disease, Nervous system disease |
|  | Carcinoma, Hepatocellular | Cancer, Digestive system disease |
|  | Cardiovascular Diseases | Cardiovascular disease |
|  | Celiac Disease | Digestive system disease, Metabolic disease |
|  | Cerebral Hemorrhage | Cardiovascular disease, Nervous system disease, Pathology (process) |
|  | Coronary Artery Disease | Cardiovascular disease |
|  | Coronary Disease | Cardiovascular disease |
|  | Coronary Restenosis | Cardiovascular disease |
|  | Cough | Respiratory tract disease, Signs and symptoms |
|  | Death, Sudden | Pathology (process) |
|  | Diabetes Mellitus, Experimental | Endocrine system disease, Metabolic disease |
|  | Diabetic Nephropathies | Endocrine system disease, Urogenital disease (female), Urogenital disease (male) |
|  | Drug Hypersensitivity | Immune system disease |
|  | Gaucher Disease | Genetic disease (inborn), Metabolic disease, Nervous system disease |
|  | Genetic Predisposition to Disease | Pathology (process) |
|  | Glomerulonephritis, IGA | Immune system disease, Urogenital disease (female), Urogenital disease (male) |
|  | Glycogen Storage Disease Type V | Genetic disease (inborn), Metabolic disease |
|  | Heart Failure | Cardiovascular disease |
|  | Hypertension | Cardiovascular disease |
|  | Hypertension, Pulmonary | Cardiovascular disease, Respiratory tract disease |
|  | Hypertrophy, Left Ventricular | Cardiovascular disease, Pathology (anatomical condition) |
|  | Hypotension | Cardiovascular disease |
|  | Infant, Premature, Diseases | Infant-newborn disease |
|  | Infertility, Male | Urogenital disease (male) |
|  | Kidney Diseases | Urogenital disease (female), Urogenital disease (male) |
|  | Kidney Failure, Chronic | Pathology (process), Urogenital disease (female), Urogenital disease (male) |
|  | Lung Diseases | Respiratory tract disease |
|  | Lung Injury | Respiratory tract disease, Wounds and injuries |
|  | Lung Neoplasms | Cancer, Respiratory tract disease |
|  | Meningococcal Infections | Bacterial infection or mycosis |
|  | MICROVASCULAR COMPLICATIONS OF DIABETES, SUSCEPTIBILITY TO, 3 | Cardiovascular disease, Endocrine system disease |
|  | Myocardial Infarction | Cardiovascular disease, Pathology (process) |
|  | Neoplasm Metastasis | Cancer, Pathology (process) |
|  | Non-alcoholic Fatty Liver Disease | Digestive system disease |
|  | Pneumonia, Viral | Respiratory tract disease, Viral disease |
|  | Pre-Eclampsia | Pregnancy complication |
|  | Prostatic Neoplasms | Cancer, Urogenital disease (male) |
|  | Psychoses, Substance-Induced | Mental disorder, Substance-related disorder |
|  | Pulmonary Fibrosis | Pathology (process), Respiratory tract disease |
|  | Renal Insufficiency | Urogenital disease (female), Urogenital disease (male) |
|  | RENAL TUBULAR DYSGENESIS | Congenital abnormality, Urogenital disease (female), Urogenital disease (male) |
|  | Respiratory Distress Syndrome | Respiratory tract disease |
|  | Respiratory Tract Diseases | Respiratory tract disease |
|  | Severe Acute Respiratory Syndrome | Respiratory tract disease, Viral disease |
|  | Staphylococcal Infections | Bacterial infection or mycosis |
|  | Stomach Neoplasms | Cancer, Digestive system disease |
|  | Stroke | Cardiovascular disease, Nervous system disease |
|  | Vascular System Injuries | Cardiovascular disease, Wounds and injuries |
|  | Weight Gain | Signs and symptoms |
|  | Weight Loss | Signs and symptoms |
| CNR2 | Anterior Cruciate Ligament Injuries | Wounds and injuries |
|  | Atherosclerosis | Cardiovascular disease |
|  | Breast Neoplasms | Cancer, Skin disease |
|  | Catalepsy | Nervous system disease, Signs and symptoms |
|  | Cholestasis | Digestive system disease |
|  | Depressive Disorder | Mental disorder |
|  | Diarrhea | Signs and symptoms |
|  | Disease Models, Animal | Animal disease |
|  | Endometrial Neoplasms | Cancer, Urogenital disease (female) |
|  | Hyperalgesia | Nervous system disease, Signs and symptoms |
|  | Hyperemia | Cardiovascular disease |
|  | Hyperglycemia | Metabolic disease |
|  | Immune System Diseases | Immune system disease |
|  | Inflammatory Bowel Diseases | Digestive system disease |
|  | Leukemia, Myeloid, Acute | Cancer |
|  | Liver Cirrhosis | Digestive system disease, Pathology (process) |
|  | Pancreatic Neoplasms | Cancer, Digestive system disease, Endocrine system disease |
|  | Pancreatitis | Digestive system disease |
|  | Parkinson Disease, Secondary | Nervous system disease |
|  | Scleroderma, Systemic | Connective tissue disease, Skin disease |
|  | Stress Disorders, Post-Traumatic | Mental disorder |
|  | Weight Loss | Signs and symptoms |
| B1 | Alzheimer Disease | Mental disorder, Nervous system disease |
| PER2 | Advanced Sleep-Phase Syndrome, Familial | Mental disorder, Nervous system disease, Occupational disease |
|  | Alcoholic Intoxication | Mental disorder, Substance-related disorder |
|  | Leukemia, Myelogenous, Chronic, BCR-ABL Positive | Blood disease, Cancer, Pathology (process) |
|  | Substance Withdrawal Syndrome | Mental disorder, Substance-related disorder |
| IDO1 | Breast Neoplasms | Cancer, Skin disease |
|  | Endometriosis | Urogenital disease (female) |
|  | Inflammation | Pathology (process) |
|  | Neoplasm Recurrence, Local | Cancer, Pathology (process) |
|  | Neurodegenerative Diseases | Nervous system disease |
|  | Obesity | Nutrition disorder, Signs and symptoms |
|  | Weight Gain | Signs and symptoms |
| GCGR | Acth-Independent Macronodular Adrenal Hyperplasia | Endocrine system disease |
| GRM5 | Attention Deficit Disorder with Hyperactivity | Mental disorder |
|  | Cocaine-Related Disorders | Mental disorder, Substance-related disorder |
|  | Diabetic Neuropathies | Endocrine system disease, Nervous system disease |
|  | Epilepsy, Temporal Lobe | Nervous system disease |
|  | Neuralgia | Nervous system disease, Signs and symptoms |
|  | Status Epilepticus | Nervous system disease, Signs and symptoms |
|  | Substance-Related Disorders | Mental disorder, Substance-related disorder |
| HRH3 | Amnesia | Mental disorder, Nervous system disease, Signs and symptoms |
|  | Hypertension | Cardiovascular disease |
|  | Memory Disorders | Nervous system disease, Signs and symptoms |
| SLC10A2 | Bile Acid Malabsorption, Primary | Digestive system disease, Metabolic disease, Signs and symptoms |
|  | Cognition Disorders | Mental disorder |
|  | Diabetes Complications | Endocrine system disease |
|  | Nervous system diseases | Nervous system disease |
|  | Substance-Related Disorders | Mental disorder, Substance-related disorder |
| CASR | Epilepsy, Idiopathic Generalized | Nervous system disease |
|  | Hypercalcemia | Metabolic disease |
|  | Hyperparathyroidism | Endocrine system disease |
|  | Hyperparathyroidism, Neonatal Severe Primary | Endocrine system disease, Infant-newborn disease |
|  | Hypocalcemia | Metabolic disease |
|  | Hypocalcemia, Autosomal Dominant, with Bartter Syndrome | Endocrine system disease, Metabolic disease, Urogenital disease (female), Urogenital disease (male) |
|  | Hypocalciuric hypercalcemia, familial, type 1 | Metabolic disease |
|  | Hypoparathyroidism | Endocrine system disease |
|  | Hypoparathyroidism familial isolated | Endocrine system disease |
| PDE2A | Breast Neoplasms | Cancer, Skin disease |
| PDE10A | Dyskinesia, limb and orofacial, infantile-onset | Nervous system disease, Signs and symptoms |
|  | Striatal degeneration, autosomal dominant 2 | Nervous system disease |
| OPRL1 | Arrhythmias, Cardiac | Cardiovascular disease, Pathology (process) |
|  | Hypokinesia | Nervous system disease, Signs and symptoms |
|  | Morphine Dependence | Mental disorder, Substance-related disorder |
|  | Substance Withdrawal Syndrome | Mental disorder, Substance-related disorder |
| CYP2C9 | Acute Kidney Injury | Urogenital disease (female), Urogenital disease (male) |
|  | Brain Neoplasms | Cancer, Nervous system disease |
|  | Cardiomyopathies | Cardiovascular disease |
|  | Chemical and Drug Induced Liver Injury | Digestive system disease |
|  | Coumarin Resistance | Genetic disease (inborn), Metabolic disease |
|  | Drug Hypersensitivity | Immune system disease |
|  | Drug-Related Side Effects and Adverse Reactions |  |
|  | Hemorrhage | Pathology (process) |
|  | Nephritis, Interstitial | Urogenital disease (female), Urogenital disease (male) |
|  | Nervous system diseases | Nervous system disease |
|  | Peptic Ulcer Hemorrhage | Digestive system disease, Pathology (process) |
|  | Warfarin Sensitivity | Genetic disease (inborn), Metabolic disease |
| CYP3A4 | Breast Neoplasms | Cancer, Skin disease |
|  | Chemical and Drug Induced Liver Injury | Digestive system disease |
|  | Hepatitis C | Digestive system disease, Viral disease |
|  | Osteosarcoma | Cancer |
|  | Prostatic Neoplasms | Cancer, Urogenital disease (male) |
|  | Torsades de Pointes | Cardiovascular disease, Pathology (process) |
| JAK3 | Amyotrophic lateral sclerosis 1 | Metabolic disease, Nervous system disease |
|  | Leukemia, Myelomonocytic, Juvenile | Blood disease, Cancer |
|  | Lymphoma, B-Cell | Cancer, Immune system disease, Lymphatic disease |
|  | Lymphoma, Extranodal NK-T-Cell | Cancer |
|  | Mediastinal Neoplasms | Cancer, Respiratory tract disease |
|  | Severe Combined Immunodeficiency | Genetic disease (inborn), Immune system disease, Infant-newborn disease, Metabolic disease |
|  | Severe Combined Immunodeficiency, Autosomal Recessive, T Cell-Negative, B Cell-Positive, NK Cell-Negative | Genetic disease (inborn), Immune system disease, Infant-newborn disease, Metabolic disease |
| NPY5R | Obesity | Nutrition disorder, Signs and symptoms |
| AVPR1A | Adrenal Hyperplasia, Congenital | Congenital abnormality, Endocrine system disease, Genetic disease (inborn), Metabolic disease, Urogenital disease (female), Urogenital disease (male) |
|  | Autism Spectrum Disorder | Mental disorder |
|  | Autistic Disorder | Mental disorder |
| MAPK8 | Cell Transformation, Neoplastic | Cancer, Pathology (process) |
|  | Cholestasis, Extrahepatic | Digestive system disease |
|  | Dermatitis, Atopic | Genetic disease (inborn), Immune system disease, Skin disease |
|  | Disease Models, Animal | Animal disease |
|  | Hyperalgesia | Nervous system disease, Signs and symptoms |
|  | Liver Failure, Acute | Digestive system disease |
|  | PreCancerous Conditions | Cancer |
|  | Reperfusion Injury | Cardiovascular disease, Pathology (process) |
|  | Stomach Neoplasms | Cancer, Digestive system disease |
|  | Trigeminal Neuralgia | Mouth disease, Nervous system disease |
| PRKCD | AUTOIMMUNE LYMPHOPROLIFERATIVE SYNDROME, TYPE III | Genetic disease (inborn), Immune system disease, Lymphatic disease |
|  | Chemical and Drug Induced Liver Injury | Digestive system disease |
|  | Diabetes Mellitus, Experimental | Endocrine system disease, Metabolic disease |
|  | Fever | Signs and symptoms |
|  | Hypertension | Cardiovascular disease |
|  | Hypothermia | Signs and symptoms |
|  | Intestinal Neoplasms | Cancer, Digestive system disease |
|  | Liver Cirrhosis, Experimental | Digestive system disease, Pathology (process) |
|  | Neurotoxicity Syndromes | Nervous system disease |
|  | Non-alcoholic Fatty Liver Disease | Digestive system disease |
|  | Parkinsonian Disorders | Nervous system disease |
|  | Seizures | Nervous system disease, Signs and symptoms |
| KCNH2 | Arrhythmias, Cardiac | Cardiovascular disease, Pathology (process) |
|  | Atrial Fibrillation | Cardiovascular disease, Pathology (process) |
|  | Cell Transformation, Neoplastic | Cancer, Pathology (process) |
|  | Colonic Neoplasms | Cancer, Digestive system disease |
|  | Long QT Syndrome | Cardiovascular disease, Congenital abnormality, Pathology (process) |
|  | Long Qt Syndrome 2 | Cardiovascular disease, Congenital abnormality, Pathology (process) |
|  | Muscular Atrophy | Nervous system disease, Pathology (anatomical condition), Signs and symptoms |
|  | Neoplasms | Cancer |
|  | Short QT Syndrome 1 | Cardiovascular disease, Congenital abnormality, Pathology (process) |
|  | Torsades de Pointes | Cardiovascular disease, Pathology (process) |
| F2R | Fatty Liver | Digestive system disease |
|  | Hepatitis | Digestive system disease |
|  | Inflammation | Pathology (process) |
|  | Liver Cirrhosis | Digestive system disease, Pathology (process) |
|  | Liver Cirrhosis, Experimental | Digestive system disease, Pathology (process) |
|  | Neoplasms, Experimental | Cancer |
|  | Stomach Neoplasms | Cancer, Digestive system disease |
| PABPC1 | Breast Neoplasms | Cancer, Skin disease |
|  | Carcinoma, Transitional Cell | Cancer |
|  | Urinary Bladder Neoplasms | Cancer, Urogenital disease (female), Urogenital disease (male) |
| MAPK14 | Cardiomyopathies | Cardiovascular disease |
|  | Cholestasis | Digestive system disease |
|  | Cholestasis, Extrahepatic | Digestive system disease |
|  | Hepatic Insufficiency | Digestive system disease |
|  | Hepatomegaly | Digestive system disease, Pathology (anatomical condition) |
|  | Leukemia, Myelogenous, Chronic, BCR-ABL Positive | Blood disease, Cancer, Pathology (process) |
|  | Liver Cirrhosis, Biliary | Digestive system disease, Pathology (process) |
|  | Liver Neoplasms | Cancer, Digestive system disease |
|  | Lung Neoplasms | Cancer, Respiratory tract disease |
|  | Myocardial Ischemia | Cardiovascular disease |
| CCR1 | Behcet Syndrome | Cardiovascular disease, Eye disease, Genetic disease (inborn), Mouth disease, Skin disease |
|  | Carcinoma, Hepatocellular | Cancer, Digestive system disease |
|  | Dermatitis, Contact | Skin disease |
|  | Hypersensitivity | Immune system disease |
|  | Liver Diseases | Digestive system disease |
|  | Pneumonia | Respiratory tract disease |
| KCNA5 | Atrial Fibrillation | Cardiovascular disease, Pathology (process) |
|  | Atrial Fibrillation, Familial, 7 | Cardiovascular disease, Pathology (process) |
|  | Hypertension, Pulmonary | Cardiovascular disease, Respiratory tract disease |
| PYGL | Carcinoma, Hepatocellular | Cancer, Digestive system disease |
|  | Chemical and Drug Induced Liver Injury | Digestive system disease |
|  | Glycogen Storage Disease Type VI | Genetic disease (inborn), Metabolic disease |
| GRM2 | Amphetamine-Related Disorders | Mental disorder, Substance-related disorder |
|  | Epilepsy, Temporal Lobe | Nervous system disease |
|  | Psychoses, Substance-Induced | Mental disorder, Substance-related disorder |
|  | Schizophrenia | Mental disorder |
| PGGT1B | Lung Neoplasms | Cancer, Respiratory tract disease |
| MDM2 | Acquired Hyperostosis Syndrome | Musculoskeletal disease |
|  | Breast Neoplasms | Cancer, Skin disease |
|  | Carcinoma, Non-Small-Cell Lung | Cancer, Respiratory tract disease |
|  | Cell Transformation, Neoplastic | Cancer, Pathology (process) |
|  | HIV Infections | Immune system disease, Viral disease |
|  | Infertility, Male | Urogenital disease (male) |
|  | Melanoma | Cancer |
|  | PreCancerous Conditions | Cancer |
|  | Prostatic Neoplasms | Cancer, Urogenital disease (male) |
|  | Stomach Neoplasms | Cancer, Digestive system disease |
| FDFT1 | Carcinoma, Hepatocellular | Cancer, Digestive system disease |
|  | Cocaine-Related Disorders | Mental disorder, Substance-related disorder |
|  | Marijuana Abuse | Mental disorder, Substance-related disorder |
|  | Phencyclidine Abuse | Mental disorder, Substance-related disorder |
| NR1H2 | Carcinoma, Hepatocellular | Cancer, Digestive system disease |
|  | Cholestasis | Digestive system disease |
|  | Hepatomegaly | Digestive system disease, Pathology (anatomical condition) |
| PTGS2 | Adenocarcinoma | Cancer |
|  | Adenocarcinoma Of Esophagus | Cancer, Digestive system disease |
|  | Adenomatous Polyposis Coli | Cancer, Digestive system disease, Genetic disease (inborn) |
|  | Adrenal Gland Neoplasms | Cancer, Endocrine system disease |
|  | Albuminuria | Signs and symptoms, Urogenital disease (female), Urogenital disease (male) |
|  | Amyotrophic Lateral Sclerosis | Metabolic disease, Nervous system disease |
|  | Aortic Aneurysm, Abdominal | Cardiovascular disease |
|  | Arrhythmias, Cardiac | Cardiovascular disease, Pathology (process) |
|  | Arthritis, Rheumatoid | Connective tissue disease, Immune system disease, Musculoskeletal disease |
|  | Asphyxia Neonatorum | Infant-newborn disease |
|  | Asthma, Aspirin-Induced | Immune system disease, Respiratory tract disease |
|  | Asthma, Occupational | Immune system disease, Occupational disease, Respiratory tract disease |
|  | Atherosclerosis | Cardiovascular disease |
|  | Autistic Disorder | Mental disorder |
|  | Barrett Esophagus | Cancer, Digestive system disease |
|  | Bone Neoplasms | Cancer, Musculoskeletal disease |
|  | Brain Injuries | Nervous system disease, Wounds and injuries |
|  | Brain Injuries, Traumatic | Nervous system disease, Wounds and injuries |
|  | Brain Ischemia | Cardiovascular disease, Nervous system disease |
|  | Breast Neoplasms | Cancer, Skin disease |
|  | Burns | Wounds and injuries |
|  | Cxia | Signs and symptoms |
|  | Carcinoma | Cancer |
|  | Carcinoma, Ductal, Breast | Cancer, Skin disease |
|  | Carcinoma, Hepatocellular | Cancer, Digestive system disease |
|  | Carcinoma in Situ | Cancer |
|  | Carcinoma, Renal Cell | Cancer, Urogenital disease (female), Urogenital disease (male) |
|  | Carcinoma, Squamous Cell | Cancer |
|  | Carcinoma, Transitional Cell | Cancer |
|  | Cardiomyopathies | Cardiovascular disease |
|  | Cardiovascular Diseases | Cardiovascular disease |
|  | Chemical and Drug Induced Liver Injury | Digestive system disease |
|  | Cholangiocarcinoma | Cancer |
|  | Cognition Disorders | Mental disorder |
|  | Colitis | Digestive system disease |
|  | Colonic Neoplasms | Cancer, Digestive system disease |
|  | Colorectal Neoplasms | Cancer, Digestive system disease |
|  | Coronary Artery Disease | Cardiovascular disease |
|  | Depressive Disorder | Mental disorder |
|  | Diabetes Mellitus | Endocrine system disease, Metabolic disease |
|  | Diabetes Mellitus, Experimental | Endocrine system disease, Metabolic disease |
|  | Drug-Related Side Effects and Adverse Reactions |  |
|  | Duodenal Ulcer | Digestive system disease |
|  | Edema | Signs and symptoms |
|  | Endometriosis | Urogenital disease (female) |
|  | Esophageal Neoplasms | Cancer, Digestive system disease |
|  | Esophageal Squamous Cell Carcinoma | Cancer, Digestive system disease |
|  | Fever | Signs and symptoms |
|  | Fibrosis | Pathology (process) |
|  | Gastroesophageal Reflux | Digestive system disease |
|  | Glaucoma, Open-Angle | Eye disease |
|  | Glioma | Cancer |
|  | Graves Ophthalmopathy | Endocrine system disease, Eye disease, Genetic disease (inborn), Immune system disease |
|  | Heart Failure | Cardiovascular disease |
|  | Hydronephrosis | Urogenital disease (female), Urogenital disease (male) |
|  | Hyperalgesia | Nervous system disease, Signs and symptoms |
|  | Hyperemia | Cardiovascular disease |
|  | Hyperglycemia | Metabolic disease |
|  | Hyperplasia | Pathology (process) |
|  | Hypertension | Cardiovascular disease |
|  | Inflammation | Pathology (process) |
|  | Inflammatory Bowel Diseases | Digestive system disease |
|  | Intestinal Polyps | Pathology (anatomical condition) |
|  | Kidney Diseases | Urogenital disease (female), Urogenital disease (male) |
|  | Kidney Failure, Chronic | Pathology (process), Urogenital disease (female), Urogenital disease (male) |
|  | Kidney Neoplasms | Cancer, Urogenital disease (female), Urogenital disease (male) |
|  | Leiomyosarcoma | Cancer |
|  | Leukemia, Lymphocytic, Chronic, B-Cell | Cancer, Immune system disease, Lymphatic disease, Pathology (process) |
|  | Leukoplakia, Oral | Cancer, Mouth disease, Pathology (anatomical condition) |
|  | Liver Cirrhosis, Experimental | Digestive system disease, Pathology (process) |
|  | Lung Diseases | Respiratory tract disease |
|  | Lupus Erythematosus, Systemic | Connective tissue disease, Immune system disease |
|  | Lymphoma, B-Cell | Cancer, Immune system disease, Lymphatic disease |
|  | Melanoma | Cancer |
|  | Mouth Diseases | Mouth disease |
|  | Mouth Neoplasms | Cancer, Mouth disease |
|  | Neoplasm Metastasis | Cancer, Pathology (process) |
|  | Neoplasm Recurrence, Local | Cancer, Pathology (process) |
|  | Neoplasms | Cancer |
|  | Nervous system diseases | Nervous system disease |
|  | Neurogenic Inflammation | Nervous system disease, Pathology (process) |
|  | Obesity | Nutrition disorder, Signs and symptoms |
|  | Oral Submucous Fibrosis | Mouth disease |
|  | Osteoarthritis | Musculoskeletal disease |
|  | Pancreatic Neoplasms | Cancer, Digestive system disease, Endocrine system disease |
|  | Pancreatitis | Digestive system disease |
|  | Papilloma | Cancer |
|  | Pericardial Effusion | Cardiovascular disease |
|  | PreCancerous Conditions | Cancer |
|  | Premature Birth | Pregnancy complication |
|  | Prostatic Neoplasms | Cancer, Urogenital disease (male) |
|  | Proteinuria | Signs and symptoms, Urogenital disease (female), Urogenital disease (male) |
|  | Puberty, Precocious | Endocrine system disease |
|  | Reperfusion Injury | Cardiovascular disease, Pathology (process) |
|  | Rhinitis, Allergic | Ear-nose-throat disease, Immune system disease, Respiratory tract disease |
|  | Seizures | Nervous system disease, Signs and symptoms |
|  | Skin Diseases | Skin disease |
|  | Skin Neoplasms | Cancer, Skin disease |
|  | Squamous Cell Carcinoma of Head and Neck | Cancer |
|  | Status Epilepticus | Nervous system disease, Signs and symptoms |
|  | Stomach Neoplasms | Cancer, Digestive system disease |
|  | Stomach Ulcer | Digestive system disease |
|  | Stroke | Cardiovascular disease, Nervous system disease |
|  | Thrombosis | Cardiovascular disease |
|  | Thyroid Neoplasms | Cancer, Endocrine system disease |
|  | Tongue Neoplasms | Cancer, Mouth disease |
|  | Urinary Bladder Neck Obstruction | Urogenital disease (female), Urogenital disease (male) |
|  | Urinary Bladder Neoplasms | Cancer, Urogenital disease (female), Urogenital disease (male) |
| AVPR2 | Adrenal Hyperplasia, Congenital | Congenital abnormality, Endocrine system disease, Genetic disease (inborn), Metabolic disease, Urogenital disease (female), Urogenital disease (male) |
|  | Cardiomyopathies | Cardiovascular disease |
|  | Diabetes Insipidus, Nephrogenic | Urogenital disease (female), Urogenital disease (male) |
|  | Genetic Diseases, X-Linked | Genetic disease (inborn) |
|  | Growth Disorders | Pathology (process) |
|  | Heart Failure | Cardiovascular disease |
|  | Nephrogenic Syndrome of Inappropriate Antidiuresis | Endocrine system disease, Genetic disease (inborn), Metabolic disease, Nervous system disease |
|  | Oliguria | Signs and symptoms, Urogenital disease (female), Urogenital disease (male) |
| OXTR | Autism Spectrum Disorder | Mental disorder |
|  | Autistic Disorder | Mental disorder |
|  | Carcinoma, Non-Small-Cell Lung | Cancer, Respiratory tract disease |
|  | Chromosome 3, monosomy 3p25 | Pathology (process) |
|  | Cognition Disorders | Mental disorder |
|  | Schizophrenia | Mental disorder |
| CHRM3 | Ascites | Pathology (process) |
|  | Chemical and Drug Induced Liver Injury | Digestive system disease |
|  | Cryptorchidism | Congenital abnormality, Endocrine system disease, Urogenital disease (male) |
|  | Liver Cirrhosis | Digestive system disease, Pathology (process) |
|  | Prune Belly Syndrome | Congenital abnormality |
|  | Urinary Bladder Diseases | Urogenital disease (female), Urogenital disease (male) |
| KDR | Adenocarcinoma | Cancer |
|  | Breast Neoplasms | Cancer, Skin disease |
|  | Cerebral Hemorrhage | Cardiovascular disease, Nervous system disease, Pathology (process) |
|  | Colorectal Neoplasms | Cancer, Digestive system disease |
|  | Diabetic Nephropathies | Endocrine system disease, Urogenital disease (female), Urogenital disease (male) |
|  | Epilepsy, Temporal Lobe | Nervous system disease |
|  | Esophageal Neoplasms | Cancer, Digestive system disease |
|  | Hemangioma, capillary infantile | Cancer, Genetic disease (inborn) |
|  | Hemangiosarcoma | Cancer |
|  | Liver Neoplasms, Experimental | Cancer, Digestive system disease |
|  | Myocardial Ischemia | Cardiovascular disease |
|  | Neovascularization, Pathologic | Pathology (process) |
|  | Osteochondrodysplasias | Genetic disease (inborn), Musculoskeletal disease |
|  | Pancreatic Neoplasms | Cancer, Digestive system disease, Endocrine system disease |
|  | Reperfusion Injury | Cardiovascular disease, Pathology (process) |
|  | Schizophrenia | Mental disorder |
|  | Uremia | Urogenital disease (female), Urogenital disease (male) |
|  | Venous Thrombosis | Cardiovascular disease |
| NOS1 | Amphetamine-Related Disorders | Mental disorder, Substance-related disorder |
|  | Cerebellar Diseases | Nervous system disease |
|  | Depressive Disorder | Mental disorder |
|  | Diabetes Mellitus, Type 1 | Endocrine system disease, Immune system disease, Metabolic disease |
|  | End Stage Liver Disease | Digestive system disease |
|  | Fetal Alcohol Spectrum Disorders | Fetal disease, Pregnancy complication, Substance-related disorder |
|  | Fever | Signs and symptoms |
|  | Hepatic Encephalopathy | Digestive system disease, Metabolic disease, Nervous system disease |
|  | Hyperalgesia | Nervous system disease, Signs and symptoms |
|  | Hypoxia | Signs and symptoms |
|  | Intestinal Perforation | Digestive system disease |
|  | Mental disorders | Mental disorder |
|  | Morphine Dependence | Mental disorder, Substance-related disorder |
|  | Nerve Degeneration | Pathology (process) |
|  | Nervous system diseases | Nervous system disease |
|  | Neurobehavioral Manifestations | Nervous system disease, Signs and symptoms |
|  | Parkinson Disease | Nervous system disease |
|  | Radiation Injuries, Experimental | Wounds and injuries |
|  | Schizophrenia | Mental disorder |
|  | Seizures | Nervous system disease, Signs and symptoms |
|  | Status Epilepticus | Nervous system disease, Signs and symptoms |
|  | Trauma, Nervous System | Nervous system disease, Wounds and injuries |
|  | Urination Disorders | Urogenital disease (female), Urogenital disease (male) |
|  | Ventricular Remodeling | Pathology (anatomical condition) |
| AKR1C3 | Cell Transformation, Neoplastic | Cancer, Pathology (process) |
|  | Disorders of Sex Development | Congenital abnormality, Endocrine system disease, Urogenital disease (female), Urogenital disease (male) |
|  | Endometrial Neoplasms | Cancer, Urogenital disease (female) |
|  | Endometriosis | Urogenital disease (female) |
|  | Liver Cirrhosis, Experimental | Digestive system disease, Pathology (process) |
|  | Neoplasms, Hormone-Dependent | Cancer |
|  | Obesity | Nutrition disorder, Signs and symptoms |
|  | Polycystic Ovary Syndrome | Cancer, Endocrine system disease, Urogenital disease (female) |
|  | Prostatic Neoplasms | Cancer, Urogenital disease (male) |
|  | Prostatic Neoplasms, Castration-Resistant | Cancer, Urogenital disease (male) |
|  | Stomach Neoplasms | Cancer, Digestive system disease |
| IL6ST | Arthritis, Rheumatoid | Connective tissue disease, Immune system disease, Musculoskeletal disease |
|  | Carcinoma | Cancer |
|  | Colorectal Neoplasms | Cancer, Digestive system disease |
|  | Hypersensitivity | Immune system disease |
|  | Infarction, Middle Cerebral Artery | Cardiovascular disease, Nervous system disease, Pathology (process) |
|  | Mammary Neoplasms, Animal | Animal disease, Cancer |
|  | Mammary Neoplasms, Experimental | Cancer |
|  | Myocardial Ischemia | Cardiovascular disease |
|  | Ovarian Neoplasms | Cancer, Endocrine system disease, Urogenital disease (female) |
|  | Pneumonia | Respiratory tract disease |
|  | Prostatic Neoplasms | Cancer, Urogenital disease (male) |
|  | Reperfusion Injury | Cardiovascular disease, Pathology (process) |
| SCN9A | Chronic Pain | Signs and symptoms |
|  | Erythromelalgia | Cardiovascular disease |
|  | Herpesviridae Infections | Viral disease |
|  | Indifference to Pain, Congenital, Autosomal Recessive | Genetic disease (inborn), Nervous system disease, Pathology (process) |
|  | Neuralgia | Nervous system disease, Signs and symptoms |
|  | Neuralgia, Postherpetic | Nervous system disease, Signs and symptoms |
|  | NEUROPATHY, HEREDITARY SENSORY AND AUTONOMIC, TYPE IIA | Congenital abnormality, Genetic disease (inborn), Nervous system disease |
|  | Paroxysmal Extreme Pain Disorder | Signs and symptoms |
|  | Pruritus | Signs and symptoms, Skin disease |
| NPY2R | Alcoholism | Mental disorder, Substance-related disorder |
|  | Cocaine-Related Disorders | Mental disorder, Substance-related disorder |
|  | Epilepsy | Nervous system disease |
|  | Epilepsy, Temporal Lobe | Nervous system disease |
|  | Substance Withdrawal Syndrome | Mental disorder, Substance-related disorder |
| NR1I2 | Adenocarcinoma | Cancer |
|  | Barrett Esophagus | Cancer, Digestive system disease |
|  | Calcinosis | Metabolic disease |
|  | Chemical and Drug Induced Liver Injury | Digestive system disease |
|  | Diabetes Mellitus | Endocrine system disease, Metabolic disease |
|  | Esophageal Neoplasms | Cancer, Digestive system disease |
|  | Fatty Liver | Digestive system disease |
|  | Hepatomegaly | Digestive system disease, Pathology (anatomical condition) |
|  | Hypophosphatemia | Metabolic disease |
|  | Infertility, Female | Urogenital disease (female) |
|  | Jaundice, Neonatal | Infant-newborn disease, Pathology (process) |
|  | Metabolic Syndrome | Metabolic disease |
|  | Obesity | Nutrition disorder, Signs and symptoms |
|  | Osteosarcoma | Cancer |
|  | Plaque, Atherosclerotic | Pathology (anatomical condition) |
| FKBP1A | Esophageal Squamous Cell Carcinoma | Cancer, Digestive system disease |
|  | Liver Cirrhosis, Experimental | Digestive system disease, Pathology (process) |
| EIF2AK1 | Anemia, Hemolytic | Blood disease |
|  | Chemical and Drug Induced Liver Injury | Digestive system disease |
|  | Non-alcoholic Fatty Liver Disease | Digestive system disease |
|  | Respiratory Distress Syndrome | Respiratory tract disease |
|  | Splenomegaly | Pathology (anatomical condition) |
| CHRM4 | Catatonia | Nervous system disease, Signs and symptoms |
|  | Tremor | Nervous system disease, Signs and symptoms |
| CHRM5 | Cocaine-Related Disorders | Mental disorder, Substance-related disorder |
|  | Substance Withdrawal Syndrome | Mental disorder, Substance-related disorder |
| C5AR1 | Liver Cirrhosis, Experimental | Digestive system disease, Pathology (process) |
|  | Shock, Septic | Pathology (process) |
| GCK | Congenital Hyperinsulinism | Digestive system disease, Infant-newborn disease, Metabolic disease |
|  | Diabetes Mellitus, Experimental | Endocrine system disease, Metabolic disease |
|  | Diabetes Mellitus, Permanent Neonatal | Endocrine system disease, Metabolic disease |
|  | Diabetes Mellitus, Type 2 | Endocrine system disease, Metabolic disease |
|  | Hyperglycemia | Metabolic disease |
|  | Hyperinsulinemic hypoglycemia, familial, 3 | Metabolic disease |
|  | Liver Cirrhosis, Experimental | Digestive system disease, Pathology (process) |
|  | Maturity-Onset Diabetes of the Young, Type 2 | Endocrine system disease, Metabolic disease |
| CSF1R | Breast Neoplasms | Cancer, Skin disease |
|  | Hereditary Diffuse Leukoencephalopathy with Spheroids | Nervous system disease |
|  | Leukemia, Myeloid, Acute | Cancer |
|  | Leukoencephalopathies | Nervous system disease |
|  | Liver Cirrhosis, Experimental | Digestive system disease, Pathology (process) |
|  | Neoplasm Metastasis | Cancer, Pathology (process) |
|  | Neoplasms | Cancer |
| KIT | Breast Neoplasms | Cancer, Skin disease |
|  | Carcinoma, Non-Small-Cell Lung | Cancer, Respiratory tract disease |
|  | Gastrointestinal Stromal Tumors | Cancer, Digestive system disease |
|  | Leukemia, Myeloid, Acute | Cancer |
|  | Leukemia, Promyelocytic, Acute | Cancer |
|  | Lung Diseases | Respiratory tract disease |
|  | Mastocytosis | Cancer, Immune system disease |
|  | Mastocytosis, Systemic | Cancer, Immune system disease |
|  | Mesothelioma, Malignant | Cancer, Respiratory tract disease |
|  | Neoplasm Recurrence, Local | Cancer, Pathology (process) |
|  | Neuroectodermal Tumors, Primitive, Peripheral | Cancer |
|  | Piebaldism | Genetic disease (inborn), Metabolic disease, Skin disease |
|  | Small Cell Lung Carcinoma | Cancer, Respiratory tract disease |
|  | Testicular Germ Cell Tumor | Cancer, Endocrine system disease, Urogenital disease (male) |
| PIK3CD | Immunodeficiency 14a, autosomal dominant | Immune system disease |
|  | Lymphoma, Large B-Cell, Diffuse | Cancer, Immune system disease, Lymphatic disease |
|  | Lymphoma, Mantle-Cell | Cancer, Immune system disease, Lymphatic disease |
|  | Prostatic Neoplasms | Cancer, Urogenital disease (male) |
|  | Roifman-Chitayat Syndrome | Eye disease, Genetic disease (inborn), Immune system disease, Mental disorder, Musculoskeletal disease, Nervous system disease, Pathology (process) |
| PRKDC | Carcinoma, Adenoid Cystic | Cancer |
|  | Carcinoma, Squamous Cell | Cancer |
|  | Chemical and Drug Induced Liver Injury | Digestive system disease |
|  | Hepatic Encephalopathy | Digestive system disease, Metabolic disease, Nervous system disease |
|  | IMMUNODEFICIENCY 26 WITH OR WITHOUT NEUROLOGIC ABNORMALITIES | Immune system disease, Nervous system disease, Signs and symptoms |
|  | Lung Diseases | Respiratory tract disease |
|  | Massive Hepatic Necrosis | Digestive system disease |
|  | Poisoning |  |
|  | Prostatic Neoplasms | Cancer, Urogenital disease (male) |
| HSD17B3 | 17-Hydroxysteroid Dehydrogenase Deficiency | Congenital abnormality, Endocrine system disease, Genetic disease (inborn), Metabolic disease, Skin disease, Urogenital disease (female), Urogenital disease (male) |
|  | Disorders of Sex Development | Congenital abnormality, Endocrine system disease, Urogenital disease (female), Urogenital disease (male) |
|  | Prostatic Neoplasms | Cancer, Urogenital disease (male) |
| CYP2A6 | Adenocarcinoma | Cancer |
|  | Brain Diseases | Nervous system disease |
|  | Carcinoma, Large Cell | Cancer |
|  | Carcinoma, Small Cell | Cancer |
|  | Chemical and Drug Induced Liver Injury | Digestive system disease |
|  | Colorectal Neoplasms | Cancer, Digestive system disease |
|  | Coumarin Resistance | Genetic disease (inborn), Metabolic disease |
|  | Disease Progression | Pathology (process) |
|  | Esophageal Squamous Cell Carcinoma | Cancer, Digestive system disease |
|  | Hepatitis B | Digestive system disease, Viral disease |
|  | Hepatitis C | Digestive system disease, Viral disease |
|  | Liver Diseases, Parasitic | Digestive system disease, Parasitic disease |
|  | Lung Neoplasms | Cancer, Respiratory tract disease |
|  | Nasopharyngeal Neoplasms | Cancer, Ear-nose-throat disease, Mouth disease |
|  | Opisthorchiasis | Parasitic disease |
|  | Prostatic Neoplasms | Cancer, Urogenital disease (male) |
|  | Pulmonary Emphysema | Pathology (process), Respiratory tract disease |
|  | Stomach Neoplasms | Cancer, Digestive system disease |
|  | Tobacco addiction, susceptibility | Mental disorder, Substance-related disorder |
|  | Tobacco Use Disorder | Mental disorder, Substance-related disorder |
| PLK1 | Carcinoma, Hepatocellular | Cancer, Digestive system disease |
|  | Glioma | Cancer |
|  | Leukemia | Cancer |
| GLI2 | Basal Cell Nevus Syndrome | Cancer, Congenital abnormality, Genetic disease (inborn), Mouth disease, Musculoskeletal disease |
|  | Carcinoma, Basal Cell | Cancer |
|  | Culler-jones syndrome | Cancer, Congenital abnormality, Musculoskeletal disease, Nervous system disease |
|  | Disease Models, Animal | Animal disease |
|  | Facial Dyrphism with Multiple Malformations | Congenital abnormality, Musculoskeletal disease, Nervous system disease, Pathology (anatomical condition), Signs and symptoms |
|  | Gastrointestinal Stromal Tumors | Cancer, Digestive system disease |
|  | Holoprosencephaly | Congenital abnormality, Genetic disease (inborn), Musculoskeletal disease, Nervous system disease |
|  | Holoprosencephaly 9 | Congenital abnormality, Endocrine system disease, Genetic disease (inborn), Musculoskeletal disease, Nervous system disease |
|  | Leukemia, Promyelocytic, Acute | Cancer |
|  | Skin Neoplasms | Cancer, Skin disease |
| GLI1 | Carcinoma, Hepatocellular | Cancer, Digestive system disease |
|  | Gastrointestinal Stromal Tumors | Cancer, Digestive system disease |
|  | Leukemia, Promyelocytic, Acute | Cancer |
|  | Lymphoma, Large B-Cell, Diffuse | Cancer, Immune system disease, Lymphatic disease |
|  | Peripheral Nervous system diseases | Nervous system disease |
|  | Urinary Bladder Neoplasms | Cancer, Urogenital disease (female), Urogenital disease (male) |
| ADORA1 | Bradycardia | Cardiovascular disease, Pathology (process) |
|  | Catalepsy | Nervous system disease, Signs and symptoms |
|  | Drug Hypersensitivity | Immune system disease |
|  | Drug-Related Side Effects and Adverse Reactions |  |
|  | Genetic Predisposition to Disease | Pathology (process) |
|  | Hypotension | Cardiovascular disease |
|  | Infarction, Middle Cerebral Artery | Cardiovascular disease, Nervous system disease, Pathology (process) |
|  | Ischemia | Pathology (process) |
|  | Ischemic Attack, Transient | Cardiovascular disease, Nervous system disease |
|  | Myocardial Infarction | Cardiovascular disease, Pathology (process) |
|  | Nerve Degeneration | Pathology (process) |
|  | Prenatal Exposure Delayed Effects | Pregnancy complication |
|  | Subarachnoid Hemorrhage | Cardiovascular disease, Nervous system disease, Pathology (process) |
|  | Substance Withdrawal Syndrome | Mental disorder, Substance-related disorder |
|  | Ventricular Dysfunction | Cardiovascular disease |
| ADORA2A | Amphetamine-Related Disorders | Mental disorder, Substance-related disorder |
|  | Anxiety Disorders | Mental disorder |
|  | Arthritis, Rheumatoid | Connective tissue disease, Immune system disease, Musculoskeletal disease |
|  | Cardiomyopathies | Cardiovascular disease |
|  | Depressive Disorder | Mental disorder |
|  | Drug Hypersensitivity | Immune system disease |
|  | Drug-Related Side Effects and Adverse Reactions |  |
|  | Genetic Predisposition to Disease | Pathology (process) |
|  | Hyperkinesis | Nervous system disease, Signs and symptoms |
|  | Hypotension | Cardiovascular disease |
|  | Liver Cirrhosis, Experimental | Digestive system disease, Pathology (process) |
|  | Movement Disorders | Nervous system disease |
|  | Panic Disorder | Mental disorder |
|  | Psychoses, Substance-Induced | Mental disorder, Substance-related disorder |
|  | Reperfusion Injury | Cardiovascular disease, Pathology (process) |
|  | Seizures | Nervous system disease, Signs and symptoms |
|  | Sepsis | Pathology (process) |
|  | Sleep Initiation and Maintenance Disorders | Mental disorder, Nervous system disease |
|  | Substance Withdrawal Syndrome | Mental disorder, Substance-related disorder |
|  | Tremor | Nervous system disease, Signs and symptoms |
|  | Ventricular Dysfunction | Cardiovascular disease |
|  | Weight Loss | Signs and symptoms |
| ADORA3 | Ischemia | Pathology (process) |
|  | Liver Cirrhosis, Experimental | Digestive system disease, Pathology (process) |
|  | Myocardial Infarction | Cardiovascular disease, Pathology (process) |
| SHH | Carcinoma, Hepatocellular | Cancer, Digestive system disease |
|  | Eye Abnormalities | Congenital abnormality, Eye disease |
|  | Facial Dyrphism with Multiple Malformations | Congenital abnormality, Musculoskeletal disease, Nervous system disease, Pathology (anatomical condition), Signs and symptoms |
|  | Familial schizencephaly | Congenital abnormality, Nervous system disease |
|  | Holoprosencephaly | Congenital abnormality, Genetic disease (inborn), Musculoskeletal disease, Nervous system disease |
|  | Holoprosencephaly 3 | Congenital abnormality, Genetic disease (inborn), Musculoskeletal disease, Nervous system disease |
|  | Microphthalmia, Isolated, with Coloboma 5 | Congenital abnormality, Eye disease |
|  | Microphthalmos | Congenital abnormality, Eye disease |
|  | Parkinson Disease, Secondary | Nervous system disease |
|  | Single upper central incisor | Congenital abnormality, Mouth disease |
| OPRM1 | AIDS Dementia Complex | Immune system disease, Mental disorder, Nervous system disease, Viral disease |
|  | Alcoholism | Mental disorder, Substance-related disorder |
|  | Amphetamine-Related Disorders | Mental disorder, Substance-related disorder |
|  | Cocaine-Related Disorders | Mental disorder, Substance-related disorder |
|  | Constipation | Signs and symptoms |
|  | Dyskinesias | Nervous system disease, Signs and symptoms |
|  | Encephalitis, Viral | Nervous system disease, Viral disease |
|  | Epilepsy | Nervous system disease |
|  | Hepatic Encephalopathy | Digestive system disease, Metabolic disease, Nervous system disease |
|  | Hepatitis C | Digestive system disease, Viral disease |
|  | Heroin Dependence | Mental disorder, Substance-related disorder |
|  | Hyperalgesia | Nervous system disease, Signs and symptoms |
|  | Hypotension, Orthostatic | Cardiovascular disease, Nervous system disease |
|  | Morphine Dependence | Mental disorder, Substance-related disorder |
|  | Movement Disorders | Nervous system disease |
|  | Nausea | Signs and symptoms |
|  | Neuralgia | Nervous system disease, Signs and symptoms |
|  | Pruritus | Signs and symptoms, Skin disease |
|  | Psychoses, Substance-Induced | Mental disorder, Substance-related disorder |
|  | Respiratory Insufficiency | Respiratory tract disease |
|  | Seizures | Nervous system disease, Signs and symptoms |
|  | Substance-Related Disorders | Mental disorder, Substance-related disorder |
| OPRD1 | Epilepsy | Nervous system disease |
|  | Hypotension, Orthostatic | Cardiovascular disease, Nervous system disease |
|  | Substance-Related Disorders | Mental disorder, Substance-related disorder |
|  | Substance Withdrawal Syndrome | Mental disorder, Substance-related disorder |
| OPRK1 | Arrhythmias, Cardiac | Cardiovascular disease, Pathology (process) |
|  | Cocaine-Related Disorders | Mental disorder, Substance-related disorder |
|  | Pain | Signs and symptoms |
|  | Seizures | Nervous system disease, Signs and symptoms |
|  | Substance Withdrawal Syndrome | Mental disorder, Substance-related disorder |
| PTAFR | Crohn Disease | Digestive system disease |
|  | Liver Cirrhosis, Experimental | Digestive system disease, Pathology (process) |
|  | Polycystic Ovary Syndrome | Cancer, Endocrine system disease, Urogenital disease (female) |
| SCN5A | Atrial Fibrillation | Cardiovascular disease, Pathology (process) |
|  | ATRIAL FIBRILLATION, FAMILIAL, 10 | Cardiovascular disease, Pathology (process) |
|  | Brugada Syndrome | Cardiovascular disease, Genetic disease (inborn) |
|  | Cardiomyopathy, Dilated, 1E | Cardiovascular disease, Genetic disease (inborn) |
|  | Long QT Syndrome | Cardiovascular disease, Congenital abnormality, Pathology (process) |
|  | Long Qt Syndrome 3 | Cardiovascular disease, Congenital abnormality, Pathology (process) |
|  | Long QT syndrome type 3 | Cardiovascular disease, Congenital abnormality, Pathology (process) |
|  | Paroxysmal ventricular fibrillation | Cardiovascular disease, Pathology (process) |
|  | PROGRESSIVE FAMILIAL HEART BLOCK, TYPE IA | Cardiovascular disease, Pathology (process) |
|  | Sick Sinus Syndrome 1, Autosomal Recessive | Cardiovascular disease, Pathology (process) |
|  | Sudden Infant Death | Pathology (process) |
|  | Tachycardia, Ventricular | Cardiovascular disease, Pathology (process) |
|  | Torsades de Pointes | Cardiovascular disease, Pathology (process) |
| ADRA1A | Carcinoma, Hepatocellular | Cancer, Digestive system disease |
|  | Diabetes Mellitus, Experimental | Endocrine system disease, Metabolic disease |
|  | Liver Cirrhosis | Digestive system disease, Pathology (process) |
|  | Ventricular Remodeling | Pathology (anatomical condition) |
| HRH4 | Adenocarcinoma | Cancer |
|  | Pruritus | Signs and symptoms, Skin disease |
|  | Stomach Neoplasms | Cancer, Digestive system disease |
| TYK2 | Arthritis, Rheumatoid | Connective tissue disease, Immune system disease, Musculoskeletal disease |
|  | Autistic Disorder | Mental disorder |
|  | Cocaine-Related Disorders | Mental disorder, Substance-related disorder |
|  | COVID-19 | Respiratory tract disease, Viral disease |
|  | Crohn Disease | Digestive system disease |
|  | Diabetes Mellitus, Type 1 | Endocrine system disease, Immune system disease, Metabolic disease |
|  | Liver Cirrhosis, Biliary | Digestive system disease, Pathology (process) |
|  | Multiple Sclerosis | Immune system disease, Nervous system disease |
|  | Paranoid Disorders | Mental disorder |
|  | Precursor T-Cell Lymphoblastic Leukemia-Lymphoma | Cancer, Immune system disease, Lymphatic disease |
|  | Psoriasis | Skin disease |
|  | Tyrosine Kinase 2 Deficiency | Blood disease, Genetic disease (inborn), Immune system disease |
| LRRK2 | Blepharoptosis | Eye disease |
|  | Carcinoma, Renal Cell | Cancer, Urogenital disease (female), Urogenital disease (male) |
|  | Endometriosis | Urogenital disease (female) |
|  | Motor Disorders | Mental disorder |
|  | Nerve Degeneration | Pathology (process) |
|  | Parkinson Disease | Nervous system disease |
|  | PARKINSON DISEASE 8, AUTOSOMAL DOMINANT | Nervous system disease |
|  | Parkinsonian Disorders | Nervous system disease |
|  | Psychomotor Disorders | Nervous system disease, Signs and symptoms |
|  | Signs and Symptoms | Signs and symptoms |
|  | Weight Loss | Signs and symptoms |
| UGT2B7 | Urinary Bladder Neoplasms | Cancer, Urogenital disease (female), Urogenital disease (male) |
| PRKCG | Disease Models, Animal | Animal disease |
|  | Hyperalgesia | Nervous system disease, Signs and symptoms |
|  | Intellectual Disability | Mental disorder, Nervous system disease, Signs and symptoms |
|  | Spinocerebellar ataxia 14 | Genetic disease (inborn), Nervous system disease |
|  | Spinocerebellar Ataxias | Genetic disease (inborn), Nervous system disease |
|  | Trigeminal Neuralgia | Mouth disease, Nervous system disease |
| PRKCA | Asthma, Occupational | Immune system disease, Occupational disease, Respiratory tract disease |
|  | Cardiomegaly | Cardiovascular disease, Pathology (anatomical condition) |
|  | Diabetes Insipidus, Nephrogenic | Urogenital disease (female), Urogenital disease (male) |
|  | Diabetes Mellitus, Experimental | Endocrine system disease, Metabolic disease |
|  | Intestinal Neoplasms | Cancer, Digestive system disease |
|  | Non-alcoholic Fatty Liver Disease | Digestive system disease |
|  | Polyuria | Signs and symptoms, Urogenital disease (female), Urogenital disease (male) |
| CNR1 | Anxiety Disorders | Mental disorder |
|  | Attention Deficit Disorder with Hyperactivity | Mental disorder |
|  | Binge-Eating Disorder | Mental disorder |
|  | Catalepsy | Nervous system disease, Signs and symptoms |
|  | Cholestasis | Digestive system disease |
|  | Cocaine-Related Disorders | Mental disorder, Substance-related disorder |
|  | Cognition Disorders | Mental disorder |
|  | Disease Models, Animal | Animal disease |
|  | Endometrial Neoplasms | Cancer, Urogenital disease (female) |
|  | Endometriosis | Urogenital disease (female) |
|  | Epilepsy, Temporal Lobe | Nervous system disease |
|  | Glucose Intolerance | Metabolic disease |
|  | Huntington Disease | Genetic disease (inborn), Mental disorder, Nervous system disease |
|  | Hyperalgesia | Nervous system disease, Signs and symptoms |
|  | Hypotension | Cardiovascular disease |
|  | Hypothermia | Signs and symptoms |
|  | Liver Cirrhosis | Digestive system disease, Pathology (process) |
|  | Liver Cirrhosis, Experimental | Digestive system disease, Pathology (process) |
|  | Marijuana Abuse | Mental disorder, Substance-related disorder |
|  | Marijuana Use | Mental disorder, Substance-related disorder |
|  | Melanoma, Cutaneous Malignant | Cancer, Skin disease |
|  | Memory Disorders | Nervous system disease, Signs and symptoms |
|  | Motor Skills Disorders | Mental disorder |
|  | Movement Disorders | Nervous system disease |
|  | Multiple Sclerosis | Immune system disease, Nervous system disease |
|  | Muscle Spasticity | Musculoskeletal disease, Nervous system disease, Signs and symptoms |
|  | Nerve Degeneration | Pathology (process) |
|  | Obesity | Nutrition disorder, Signs and symptoms |
|  | Pain | Signs and symptoms |
|  | Pancreatic Neoplasms | Cancer, Digestive system disease, Endocrine system disease |
|  | Pancreatitis | Digestive system disease |
|  | Rhabdomyosarcoma | Cancer |
|  | Schizophrenia | Mental disorder |
|  | Scleroderma, Systemic | Connective tissue disease, Skin disease |
|  | Seizures | Nervous system disease, Signs and symptoms |
|  | Status Epilepticus | Nervous system disease, Signs and symptoms |
|  | Stress Disorders, Post-Traumatic | Mental disorder |
|  | Substance-Related Disorders | Mental disorder, Substance-related disorder |
|  | Substance Withdrawal Syndrome | Mental disorder, Substance-related disorder |
|  | Weight Gain | Signs and symptoms |
|  | Weight Loss | Signs and symptoms |
| PIM1 | Breast Neoplasms | Cancer, Skin disease |
|  | Cell Transformation, Neoplastic | Cancer, Pathology (process) |
|  | Dermatitis, Allergic Contact | Immune system disease, Skin disease |
|  | Disease Models, Animal | Animal disease |
|  | Glioblastoma | Cancer |
|  | Hernias, Diaphragmatic, Congenital | Congenital abnormality, Pathology (anatomical condition) |
|  | PreCancerous Conditions | Cancer |
|  | Prostatic Intraepithelial Neoplasia | Cancer |
|  | Pyelonephritis | Urogenital disease (female), Urogenital disease (male) |
| GRIK1 | Seizures | Nervous system disease, Signs and symptoms |
| GRIA1 | Autistic Disorder | Mental disorder |
|  | Cocaine-Related Disorders | Mental disorder, Substance-related disorder |
|  | Hyperkinesis | Nervous system disease, Signs and symptoms |
|  | Hypotension | Cardiovascular disease |
|  | Learning Disabilities | Mental disorder, Nervous system disease, Signs and symptoms |
|  | Morphine Dependence | Mental disorder, Substance-related disorder |
|  | Olfaction Disorders | Nervous system disease, Signs and symptoms |
|  | Rhinitis, Allergic | Ear-nose-throat disease, Immune system disease, Respiratory tract disease |
|  | Sexual Dysfunctions, Psychological | Mental disorder |
|  | Substance Withdrawal Syndrome | Mental disorder, Substance-related disorder |
| GRIK5 | Schizophrenia | Mental disorder |
| SLC1A1 | Atrophy | Pathology (anatomical condition) |
|  | Autism Spectrum Disorder | Mental disorder |
|  | Brain Diseases | Nervous system disease |
|  | Cognition Disorders | Mental disorder |
|  | Dicarboxylicaminoaciduria | Genetic disease (inborn), Mental disorder, Nervous system disease, Signs and symptoms, Urogenital disease (female), Urogenital disease (male) |
|  | Endometriosis | Urogenital disease (female) |
|  | Epilepsy | Nervous system disease |
|  | Epilepsy, Temporal Lobe | Nervous system disease |
|  | Low Tension Glaucoma | Eye disease |
|  | Malformations of Cortical Development | Congenital abnormality, Nervous system disease |
|  | Nerve Degeneration | Pathology (process) |
|  | Retinal Degeneration | Eye disease |
|  | Schizophrenia | Mental disorder |
|  | Seizures | Nervous system disease, Signs and symptoms |
| GRM4 | Epilepsy, Temporal Lobe | Nervous system disease |
|  | Nerve Degeneration | Pathology (process) |
|  | Osteosarcoma | Cancer |
| GRM3 | Epilepsy, Temporal Lobe | Nervous system disease |
|  | Melanoma | Cancer |
|  | Schizophrenia | Mental disorder |
| GRIA4 | Myopia | Eye disease |
|  | Substance-Related Disorders | Mental disorder, Substance-related disorder |
| GRM8 | Anxiety Disorders | Mental disorder |
|  | Attention Deficit Disorder with Hyperactivity | Mental disorder |
|  | Autistic Disorder | Mental disorder |
|  | Brain Injuries | Nervous system disease, Wounds and injuries |
|  | Gliosis | Pathology (process) |
|  | Small Cell Lung Carcinoma | Cancer, Respiratory tract disease |
| GRIK2 | Autistic Disorder | Mental disorder |
|  | Bipolar Disorder | Mental disorder |
|  | Breast Neoplasms | Cancer, Skin disease |
|  | Carcinoma, Non-Small-Cell Lung | Cancer, Respiratory tract disease |
|  | Colonic Neoplasms | Cancer, Digestive system disease |
|  | Esophageal Neoplasms | Cancer, Digestive system disease |
|  | Glioblastoma | Cancer |
|  | Liver Neoplasms | Cancer, Digestive system disease |
|  | Lymphatic Metastasis | Cancer, Pathology (process) |
|  | Mental Retardation, Autosomal Recessive 6 | Mental disorder, Nervous system disease, Signs and symptoms |
|  | Ovarian Neoplasms | Cancer, Endocrine system disease, Urogenital disease (female) |
|  | Schizophrenia | Mental disorder |
|  | Sexual Dysfunctions, Psychological | Mental disorder |
|  | Substance-Related Disorders | Mental disorder, Substance-related disorder |
| GRIK3 | Small Cell Lung Carcinoma | Cancer, Respiratory tract disease |
| GRM1 | Attention Deficit Disorder with Hyperactivity | Mental disorder |
|  | Epilepsy | Nervous system disease |
|  | Epilepsy, Temporal Lobe | Nervous system disease |
|  | Fibroma | Cancer |
|  | Neoplasms, Bone Tissue | Cancer |
|  | Spinocerebellar Ataxias | Genetic disease (inborn), Nervous system disease |
|  | Status Epilepticus | Nervous system disease, Signs and symptoms |
| GRM7 | Attention Deficit Disorder with Hyperactivity | Mental disorder |
|  | Substance-Related Disorders | Mental disorder, Substance-related disorder |
| GRIA2 | Cocaine-Related Disorders | Mental disorder, Substance-related disorder |
|  | Hyperkinesis | Nervous system disease, Signs and symptoms |
|  | Olfaction Disorders | Nervous system disease, Signs and symptoms |
|  | Rhinitis, Allergic | Ear-nose-throat disease, Immune system disease, Respiratory tract disease |
|  | Status Epilepticus | Nervous system disease, Signs and symptoms |
|  | Substance Withdrawal Syndrome | Mental disorder, Substance-related disorder |
| GRM6 | Night blindness, congenital stationary | Eye disease, Genetic disease (inborn) |
| SLC1A2 | Amyotrophic Lateral Sclerosis | Metabolic disease, Nervous system disease |
|  | Cocaine-Related Disorders | Mental disorder, Substance-related disorder |
|  | DEVELOPMENTAL AND EPILEPTIC ENCEPHALOPATHY 41 | Nervous system disease |
|  | Diabetes Mellitus, Type 2 | Endocrine system disease, Metabolic disease |
|  | Epilepsy | Nervous system disease |
|  | Nerve Degeneration | Pathology (process) |
|  | Stomach Neoplasms | Cancer, Digestive system disease |
| SLC6A12 | Neoplasm Invasiveness | Cancer, Pathology (process) |
| SLC6A1 | Amyotrophic Lateral Sclerosis | Metabolic disease, Nervous system disease |
|  | Epilepsy | Nervous system disease |
|  | MYOCLONIC-ATONIC EPILEPSY | Nervous system disease |
|  | NeurodevelopMental disorders | Mental disorder |
|  | Schizophrenia | Mental disorder |
|  | Seizures | Nervous system disease, Signs and symptoms |
|  | Substance Withdrawal Syndrome | Mental disorder, Substance-related disorder |
| SLC6A11 | Melanoma | Cancer |
| SLC22A6 | Calcinosis | Metabolic disease |
| BBOX1 | Liver Cirrhosis, Experimental | Digestive system disease, Pathology (process) |
| CDC25B | Chemical and Drug Induced Liver Injury | Digestive system disease |
|  | Heart Failure, Diastolic | Cardiovascular disease |
| GLRA1 | Dystonic Disorders | Nervous system disease |
|  | Hyperexplexia hereditary | Musculoskeletal disease, Nervous system disease, Signs and symptoms |
| DHCR7 | Autistic Disorder | Mental disorder |
|  | Bone Diseases, Metabolic | Metabolic disease, Musculoskeletal disease |
|  | Disease Models, Animal | Animal disease |
|  | Leukemia, Promyelocytic, Acute | Cancer |
|  | Liver Cirrhosis | Digestive system disease, Pathology (process) |
|  | Smith-Lemli-Opitz Syndrome | Congenital abnormality, Genetic disease (inborn), Metabolic disease |
| SMO | Ameloblastoma | Cancer |
|  | Brain Neoplasms | Cancer, Nervous system disease |
|  | Carcinoma, Basal Cell | Cancer |
|  | Gastrointestinal Stromal Tumors | Cancer, Digestive system disease |
|  | Hypothalamic hamartomas | Cancer, Nervous system disease |
|  | Infertility, Female | Urogenital disease (female) |
|  | Jaw Abnormalities | Congenital abnormality, Mouth disease, Musculoskeletal disease |
|  | Leukemia, Promyelocytic, Acute | Cancer |
|  | Maxillary Neoplasms | Cancer, Mouth disease, Musculoskeletal disease |
|  | Meningioma | Cancer, Nervous system disease |
|  | Neuroectodermal Tumors, Primitive | Cancer |
|  | Osteoarthritis, Hip | Musculoskeletal disease |
|  | Skin Neoplasms | Cancer, Skin disease |
|  | Winter Shortland Temple syndrome | Congenital abnormality, Musculoskeletal disease, Skin disease |
| HSD11B2 | Apparent mineralocorticoid excess | Genetic disease (inborn), Metabolic disease |
|  | Asthma | Immune system disease, Respiratory tract disease |
|  | Diabetes Mellitus, Type 1 | Endocrine system disease, Immune system disease, Metabolic disease |
|  | Hypertension | Cardiovascular disease |
|  | Liver Cirrhosis | Digestive system disease, Pathology (process) |
|  | Mineralocorticoid Excess Syndrome, Apparent | Genetic disease (inborn), Metabolic disease |
|  | Myocardial Infarction | Cardiovascular disease, Pathology (process) |
|  | Nephrotic Syndrome | Urogenital disease (female), Urogenital disease (male) |
|  | Pregnancy Complications | Pregnancy complication |
| CXCR3 | Alopecia Areata | Skin disease |
|  | Autoimmune Diseases | Immune system disease |
|  | Dermatitis, Atopic | Genetic disease (inborn), Immune system disease, Skin disease |
|  | Dermatitis, Contact | Skin disease |
|  | Glomerulonephritis | Urogenital disease (female), Urogenital disease (male) |
|  | Hypersensitivity | Immune system disease |
|  | Inflammation | Pathology (process) |
|  | Ischemia | Pathology (process) |
|  | Pleural Diseases | Respiratory tract disease |
|  | Pneumonia | Respiratory tract disease |
|  | Stroke | Cardiovascular disease, Nervous system disease |
| MGLL | Liver Cirrhosis | Digestive system disease, Pathology (process) |
|  | Osteoporosis | Metabolic disease, Musculoskeletal disease |
| VDR | Alopecia | Pathology (anatomical condition), Skin disease |
|  | Breast Neoplasms | Cancer, Skin disease |
|  | Carcinoma, Transitional Cell | Cancer |
|  | Epilepsy, Temporal Lobe | Nervous system disease |
|  | Genetic Diseases, Inborn | Genetic disease (inborn) |
|  | Hypertension | Cardiovascular disease |
|  | Ischemic Stroke | Cardiovascular disease, Nervous system disease |
|  | Lead Poisoning |  |
|  | Leukemia, Lymphocytic, Chronic, B-Cell | Cancer, Immune system disease, Lymphatic disease, Pathology (process) |
|  | Multiple Sclerosis | Immune system disease, Nervous system disease |
|  | Neoplasms | Cancer |
|  | Prostatic Neoplasms | Cancer, Urogenital disease (male) |
|  | Rickets | Metabolic disease, Musculoskeletal disease, Nutrition disorder |
|  | Uremia | Urogenital disease (female), Urogenital disease (male) |
|  | Vitamin D Deficiency | Nutrition disorder |
|  | Vitamin D-Dependent Rickets, Type 2A | Genetic disease (inborn), Metabolic disease, Musculoskeletal disease, Nutrition disorder, Urogenital disease (female), Urogenital disease (male) |
| EPHX1 | Abnormalities, Drug-Induced | Congenital abnormality |
|  | Acute Lung Injury | Respiratory tract disease |
|  | Amphetamine-Related Disorders | Mental disorder, Substance-related disorder |
|  | Carcinoma | Cancer |
|  | Carcinoma, Hepatocellular | Cancer, Digestive system disease |
|  | Chemical and Drug Induced Liver Injury | Digestive system disease |
|  | Fever | Signs and symptoms |
|  | Hypercholanemia, Familial | Genetic disease (inborn), Metabolic disease |
|  | Leukemia, Myeloid, Acute | Cancer |
|  | Lung Neoplasms | Cancer, Respiratory tract disease |
|  | Lymphoma | Cancer, Immune system disease, Lymphatic disease |
|  | Lymphoma, Non-Hodgkin | Cancer, Immune system disease, Lymphatic disease |
|  | Mammary Neoplasms, Animal | Animal disease, Cancer |
|  | Mammary Neoplasms, Experimental | Cancer |
|  | Mesothelioma, Malignant | Cancer, Respiratory tract disease |
|  | Micronuclei, Chromosome-Defective | Pathology (process) |
|  | Multiple Myeloma | Blood disease, Cancer, Cardiovascular disease, Immune system disease |
|  | Myocardial Ischemia | Cardiovascular disease |
|  | Precursor Cell Lymphoblastic Leukemia-Lymphoma | Cancer, Immune system disease, Lymphatic disease |
|  | Pre-Eclampsia | Pregnancy complication |
|  | Prostatic Neoplasms | Cancer, Urogenital disease (male) |
|  | Pulmonary Disease, Chronic Obstructive | Pathology (process), Respiratory tract disease |
|  | Pulmonary Emphysema | Pathology (process), Respiratory tract disease |
|  | Skin Neoplasms | Cancer, Skin disease |
| CA9 | Anaplasia | Cancer, Pathology (process) |
|  | Carcinoma, Ductal | Cancer |
|  | Carcinoma, Intraductal, Noninfiltrating | Cancer |
|  | Carcinoma, Non-Small-Cell Lung | Cancer, Respiratory tract disease |
|  | Hypertension, Pulmonary | Cardiovascular disease, Respiratory tract disease |
|  | Hypoxia | Signs and symptoms |
|  | Leukemia, Myelogenous, Chronic, BCR-ABL Positive | Blood disease, Cancer, Pathology (process) |
| CTRB1 | Hypoxia | Signs and symptoms |
| ADRA2A | Cardiomegaly | Cardiovascular disease, Pathology (anatomical condition) |
|  | Epilepsy | Nervous system disease |
|  | Fibrosis | Pathology (process) |
|  | Heart Diseases | Cardiovascular disease |
|  | Hypertension | Cardiovascular disease |
|  | Hypotension | Cardiovascular disease |
|  | Panic Disorder | Mental disorder |
| ADRA2B | Carcinoma, Hepatocellular | Cancer, Digestive system disease |
|  | Cyanosis | Signs and symptoms |
|  | Intellectual Disability | Mental disorder, Nervous system disease, Signs and symptoms |
|  | Lung Diseases | Respiratory tract disease |
| F2 | Alzheimer Disease | Mental disorder, Nervous system disease |
|  | Arthritis, Rheumatoid | Connective tissue disease, Immune system disease, Musculoskeletal disease |
|  | Blood Coagulation Disorders | Blood disease |
|  | Brain Ischemia | Cardiovascular disease, Nervous system disease |
|  | Carcinoma, Hepatocellular | Cancer, Digestive system disease |
|  | Disseminated Intravascular Coagulation | Blood disease |
|  | Fatty Liver | Digestive system disease |
|  | Hemorrhage | Pathology (process) |
|  | Hypertrophy | Pathology (anatomical condition) |
|  | Hypoprothrombinemias | Blood disease, Genetic disease (inborn) |
|  | Infarction, Middle Cerebral Artery | Cardiovascular disease, Nervous system disease, Pathology (process) |
|  | Ischemic Stroke | Cardiovascular disease, Nervous system disease |
|  | Liver Cirrhosis | Digestive system disease, Pathology (process) |
|  | Liver Failure | Digestive system disease |
|  | Mesenteric Ischemia | Cardiovascular disease, Digestive system disease |
|  | Myocardial Infarction | Cardiovascular disease, Pathology (process) |
|  | Nephrosis | Urogenital disease (female), Urogenital disease (male) |
|  | Nerve Degeneration | Pathology (process) |
|  | Non-alcoholic Fatty Liver Disease | Digestive system disease |
|  | Obesity | Nutrition disorder, Signs and symptoms |
|  | Paratuberculosis | Animal disease, Bacterial infection or mycosis |
|  | Pregnancy loss, recurrent, susceptibility | Pregnancy complication |
|  | Prothrombin deficiency, congenital | Blood disease, Genetic disease (inborn) |
|  | Schizophrenia | Mental disorder |
|  | Sinus Thrombosis, Intracranial | Cardiovascular disease, Nervous system disease |
|  | Skin Diseases, Vascular | Skin disease |
|  | Stroke | Cardiovascular disease, Nervous system disease |
|  | Thromboembolism | Cardiovascular disease |
|  | Thrombophilia | Blood disease |
|  | Thrombosis | Cardiovascular disease |
|  | Venous Thromboembolism | Cardiovascular disease |
|  | Venous Thrombosis | Cardiovascular disease |
| PRSS1 | Hereditary pancreatitis | Digestive system disease, Pathology (process) |
|  | Pancreatitis | Digestive system disease |
|  | Pancreatitis, Chronic | Digestive system disease, Pathology (process) |
| CTRC | Hereditary pancreatitis | Digestive system disease, Pathology (process) |
|  | Pancreatitis, Chronic | Digestive system disease, Pathology (process) |
| ELANE | Cyclic neutropenia | Blood disease |
|  | Neutropenia | Blood disease |
|  | Neutropenia, Severe Congenital, Autosomal Dominant 1 | Blood disease |
| NISCH | Bradycardia | Cardiovascular disease, Pathology (process) |
|  | Breast Neoplasms | Cancer, Skin disease |
|  | Hypotension | Cardiovascular disease |
| PLA2G6 | NBIA2B | Metabolic disease, Nervous system disease |
|  | Neurodegeneration with brain iron accumulation 2a | Metabolic disease, Nervous system disease |
|  | Nevus | Cancer |
|  | Parkinson disease 14, autosomal recessive | Nervous system disease |
| EGLN1 | Erythrocytosis, Familial, 3 | Blood disease |
|  | Neurotoxicity Syndromes | Nervous system disease |
