# Supplementary material for: A novel FCTF evaluation and prediction model for food efficacy based on association rule mining
Source: Front Nutr. 2023 Aug 28;10:1170084. doi: 10.3389/fnut.2023.1170084 (PMC10493461; doi:10.3389/fnut.2023.1170084)
Supplement: Supplementary file 4 [file Table_4.DOCX]

**Supplemental Table S4 Genes associated with digestive system cancers and neurological diseases**

| **Digestive system cancers-related genes** | | **neurological diseases-related genes** | |
| --- | --- | --- | --- |
| Symbol | Description | SLC6A4 | Solute Carrier Family 6 Member 4 |
| MIR146A | MicroRNA 146a | MECP2 | Methyl-CpG Binding Protein 2 |
| ACE | Angiotensin I Converting Enzyme | DRD4 | Dopamine Receptor D4 |
| VEGFA | Vascular Endothelial Growth Factor A | BDNF | Brain Derived Neurotrophic Factor |
| GHSR | Growth Hormone Secretagogue Receptor | PSEN1 | Presenilin 1 |
| TNF | Tumor Necrosis Factor | CACNA1A | Calcium Voltage-Gated Channel Subunit Alpha1 A |
| PTGS2 | Prostaglandin-Endoperoxide Synthase 2 | POLG | DNA Polymerase Gamma, Catalytic Subunit |
| MTR | 5-Methyltetrahydrofolate-Homocysteine Methyltransferase | ARSA | Arylsulfatase A |
| CYP2C19 | Cytochrome P450 Family 2 Subfamily C Member 19 | DRD2 | Dopamine Receptor D2 |
| MMP7 | Matrix Metallopeptidase 7 | COMT | Catechol-O-Methyltransferase |
| HIF1A | Hypoxia Inducible Factor 1 Subunit Alpha | TSC2 | TSC Complex Subunit 2 |
| NME1 | NME/NM23 Nucleoside Diphosphate Kinase 1 | TNF | Tumor Necrosis Factor |
| OGG1 | 8-Oxoguanine DNA Glycosylase | SLC6A3 | Solute Carrier Family 6 Member 3 |
| HOTAIR | HOX Transcript Antisense RNA | VPS13B | Vacuolar Protein Sorting 13 Homolog B |
| MALAT1 | Metastasis Associated Lung Adenocarcinoma Transcript 1 | HTR2A | 5-Hydroxytryptamine Receptor 2A |
| MIR486-1 | MicroRNA 486-1 | MAOA | Monoamine Oxidase A |
| CYP2A6 | Cytochrome P450 Family 2 Subfamily A Member 6 | HFE | Homeostatic Iron Regulator |
| MIR196A2 | MicroRNA 196a-2 | TTR | Transthyretin |
| APC | APC Regulator Of WNT Signaling Pathway | PTEN | Phosphatase And Tensin Homolog |
| CCR6 | C-C Motif Chemokine Receptor 6 | GALC | Galactosylceramidase |
| SERPINA3 | Serpin Family A Member 3 | PLA2G6 | Phospholipase A2 Group VI |
| ICOSLG | Inducible T Cell Costimulator Ligand | APOE | Apolipoprotein E |
| KDM4C | Lysine Demethylase 4C | CREBBP | CREB Binding Protein |
| H2AC18 | H2A Clustered Histone 18 | IL6 | Interleukin 6 |
| H19 | H19 Imprinted Maternally Expressed Transcript | CAMK2B | Calcium/Calmodulin Dependent Protein Kinase II Beta |
| MIR140 | MicroRNA 140 | KMT2D | Lysine Methyltransferase 2D |
| MIR143 | MicroRNA 143 | TH | Tyrosine Hydroxylase |
| MIR145 | MicroRNA 145 | VLDLR | Very Low Density Lipoprotein Receptor |
| MIR149 | MicroRNA 149 | TRAPPC9 | Trafficking Protein Particle Complex Subunit 9 |
| MIR204 | MicroRNA 204 | CYP2D6 | Cytochrome P450 Family 2 Subfamily D Member 6 |
| MIR21 | MicroRNA 21 | HTR2C | 5-Hydroxytryptamine Receptor 2C |
| MIR27A | MicroRNA 27a | INS | Insulin |
| MIR122 | MicroRNA 122 | ALB | Albumin |
| MIR126 | MicroRNA 126 | DISC1 | DISC1 Scaffold Protein |
| MIR185 | MicroRNA 185 | NGF | Nerve Growth Factor |
| MIR200C | MicroRNA 200c | ACHE | Acetylcholinesterase (Cartwright Blood Group) |
| MIR214 | MicroRNA 214 | DBH | Dopamine Beta-Hydroxylase |
| MIR223 | MicroRNA 223 | HTR1A | 5-Hydroxytryptamine Receptor 1A |
| MIR30E | MicroRNA 30e | IL1B | Interleukin 1 Beta |
| MIR34A | MicroRNA 34a | MBP | Myelin Basic Protein |
| MIR499A | MicroRNA 499a | LEP | Leptin |
| MIR100 | MicroRNA 100 | MAOB | Monoamine Oxidase B |
| MIR106B | MicroRNA 106b | NPY | Neuropeptide Y |
| MIR10B | MicroRNA 10b | APP | Amyloid Beta Precursor Protein |
| MIR125B1 | MicroRNA 125b-1 | MAPT | Microtubule Associated Protein Tau |
| MIR130A | MicroRNA 130a | MAG | Myelin Associated Glycoprotein |
| MIR135B | MicroRNA 135b | NTRK2 | Neurotrophic Receptor Tyrosine Kinase 2 |
| MIR141 | MicroRNA 141 | PRL | Prolactin |
| MIR142 | MicroRNA 142 | CNR1 | Cannabinoid Receptor 1 |
| MIR146B | MicroRNA 146b | CRH | Corticotropin Releasing Hormone |
| MIR150 | MicroRNA 150 | ABCB1 | ATP Binding Cassette Subfamily B Member 1 |
| MIR182 | MicroRNA 182 | GFAP | Glial Fibrillary Acidic Protein |
| MIR192 | MicroRNA 192 | DRD3 | Dopamine Receptor D3 |
| MIR193B | MicroRNA 193b | GRIN2B | Glutamate Ionotropic Receptor NMDA Type Subunit 2B |
| MIR196A1 | MicroRNA 196a-1 | CRP | C-Reactive Protein |
| MIR200B | MicroRNA 200b | SHANK3 | SH3 And Multiple Ankyrin Repeat Domains 3 |
| MIR211 | MicroRNA 211 | MTHFR | Methylenetetrahydrofolate Reductase |
| MIR29A | MicroRNA 29a | GRIN2A | Glutamate Ionotropic Receptor NMDA Type Subunit 2A |
| MIR30B | MicroRNA 30b | PMP22 | Peripheral Myelin Protein 22 |
| MIR34C | MicroRNA 34c | POMC | Proopiomelanocortin |
| MIR93 | MicroRNA 93 | S100B | S100 Calcium Binding Protein B |
| MIR99A | MicroRNA 99a | PDYN | Prodynorphin |
| MIR125A | MicroRNA 125a | PLP1 | Proteolipid Protein 1 |
| MIR127 | MicroRNA 127 | SNCA | Synuclein Alpha |
| MIR138-1 | MicroRNA 138-1 | CNTF | Ciliary Neurotrophic Factor |
| MIR139 | MicroRNA 139 | CREB1 | CAMP Responsive Element Binding Protein 1 |
| MIR155 | MicroRNA 155 | GAD1 | Glutamate Decarboxylase 1 |
| MIR200A | MicroRNA 200a | DRD1 | Dopamine Receptor D1 |
| MIR210 | MicroRNA 210 | MOG | Myelin Oligodendrocyte Glycoprotein |
| MIR212 | MicroRNA 212 | SLC6A2 | Solute Carrier Family 6 Member 2 |
| MIR216A | MicroRNA 216a | OXT | Oxytocin/Neurophysin I Prepropeptide |
| MIR22 | MicroRNA 22 | PRODH | Proline Dehydrogenase 1 |
| MIR221 | MicroRNA 221 | FMR1 | Fragile X Messenger Ribonucleoprotein 1 |
| MIR222 | MicroRNA 222 | ACE | Angiotensin I Converting Enzyme |
| MIR23A | MicroRNA 23a | BCHE | Butyrylcholinesterase |
| MIR27B | MicroRNA 27b | NDP | Norrin Cystine Knot Growth Factor NDP |
| MIR29B1 | MicroRNA 29b-1 | GRM5 | Glutamate Metabotropic Receptor 5 |
| MIR301A | MicroRNA 301a | GDNF | Glial Cell Derived Neurotrophic Factor |
| MIR324 | MicroRNA 324 | DRD5 | Dopamine Receptor D5 |
| MIR378A | MicroRNA 378a | HCRT | Hypocretin Neuropeptide Precursor |
| MIR483 | MicroRNA 483 | SLC1A2 | Solute Carrier Family 1 Member 2 |
| MIR574 | MicroRNA 574 | NRG1 | Neuregulin 1 |
| MIR9-1 | MicroRNA 9-1 | ENO2 | Enolase 2 |
| MIRLET7A1 | MicroRNA Let-7a-1 | AKT1 | AKT Serine/Threonine Kinase 1 |
| MIR101-1 | MicroRNA 101-1 | NTF3 | Neurotrophin 3 |
| MIR124-1 | MicroRNA 124-1 | GRIA1 | Glutamate Ionotropic Receptor AMPA Type Subunit 1 |
| MIR130B | MicroRNA 130b | TAC1 | Tachykinin Precursor 1 |
| MIR148A | MicroRNA 148a | HTR1B | 5-Hydroxytryptamine Receptor 1B |
| MIR151A | MicroRNA 151a | NOS1 | Nitric Oxide Synthase 1 |
| MIR15B | MicroRNA 15b | C9orf72 | C9orf72-SMCR8 Complex Subunit |
| MIR17 | MicroRNA 17 | SLC18A2 | Solute Carrier Family 18 Member A2 |
| MIR181B1 | MicroRNA 181b-1 | MIR146A | MicroRNA 146a |
| MIR183 | MicroRNA 183 | GRM2 | Glutamate Metabotropic Receptor 2 |
| MIR186 | MicroRNA 186 | DDC | Dopa Decarboxylase |
| MIR193A | MicroRNA 193a | GRIA2 | Glutamate Ionotropic Receptor AMPA Type Subunit 2 |
| MIR195 | MicroRNA 195 | NR3C1 | Nuclear Receptor Subfamily 3 Group C Member 1 |
| MIR199A1 | MicroRNA 199a-1 | VEGFA | Vascular Endothelial Growth Factor A |
| MIR19A | MicroRNA 19a | TPH1 | Tryptophan Hydroxylase 1 |
| MIR205 | MicroRNA 205 | OPRM1 | Opioid Receptor Mu 1 |
| MIR20A | MicroRNA 20a | FOS | Fos Proto-Oncogene, AP-1 Transcription Factor Subunit |
| MIR215 | MicroRNA 215 | IL10 | Interleukin 10 |
| MIR23B | MicroRNA 23b | GSK3B | Glycogen Synthase Kinase 3 Beta |
| MIR25 | MicroRNA 25 | MIR21 | MicroRNA 21 |
| MIR30A | MicroRNA 30a | DTNBP1 | Dystrobrevin Binding Protein 1 |
| MIR31 | MicroRNA 31 | GRIN1 | Glutamate Ionotropic Receptor NMDA Type Subunit 1 |
| MIR328 | MicroRNA 328 | GRN | Granulin Precursor |
| MIR330 | MicroRNA 330 | TSPO | Translocator Protein |
| MIR335 | MicroRNA 335 | CNTNAP2 | Contactin Associated Protein 2 |
| MIR33A | MicroRNA 33a | TARDBP | TAR DNA Binding Protein |
| MIR342 | MicroRNA 342 | ERCC6 | ERCC Excision Repair 6, Chromatin Remodeling Factor |
| MIR365A | MicroRNA 365a | IL18 | Interleukin 18 |
| MIR455 | MicroRNA 455 | HTR3A | 5-Hydroxytryptamine Receptor 3A |
| MIR582 | MicroRNA 582 | BACE1 | Beta-Secretase 1 |
| MIR590 | MicroRNA 590 | CRHR1 | Corticotropin Releasing Hormone Receptor 1 |
| MIR675 | MicroRNA 675 | CRYAB | Crystallin Alpha B |
| MIR92A1 | MicroRNA 92a-1 | IGF1 | Insulin Like Growth Factor 1 |
| MIRLET7B | MicroRNA Let-7b | SCN9A | Sodium Voltage-Gated Channel Alpha Subunit 9 |
| MIR106A | MicroRNA 106a | EPRS1 | Glutamyl-Prolyl-TRNA Synthetase 1 |
| MIR144 | MicroRNA 144 | SERPINA3 | Serpin Family A Member 3 |
| MIR181A1 | MicroRNA 181a-1 | MIR9-1 | MicroRNA 9-1 |
| MIR18A | MicroRNA 18a | MIR185 | MicroRNA 185 |
| MIR24-1 | MicroRNA 24-1 | MIR132 | MicroRNA 132 |
| MIR28 | MicroRNA 28 | SCN8A | Sodium Voltage-Gated Channel Alpha Subunit 8 |
| MIR296 | MicroRNA 296 | NRXN1 | Neurexin 1 |
| MIR29C | MicroRNA 29c | ANK3 | Ankyrin 3 |
| MIR338 | MicroRNA 338 | CCL2 | C-C Motif Chemokine Ligand 2 |
| MIR423 | MicroRNA 423 | MMP9 | Matrix Metallopeptidase 9 |
| MIR424 | MicroRNA 424 | MIR212 | MicroRNA 212 |
| MIR103A1 | MicroRNA 103a-1 | GAD2 | Glutamate Decarboxylase 2 |
| MIR1247 | MicroRNA 1247 | SNAP25 | Synaptosome Associated Protein 25 |
| MIR485 | MicroRNA 485 | HTT | Huntingtin |
| MIR129-1 | MicroRNA 129-1 | CHAT | Choline O-Acetyltransferase |
| MIR133A1 | MicroRNA 133a-1 | FKBP5 | FKBP Prolyl Isomerase 5 |
| MIR224 | MicroRNA 224 | MIR155 | MicroRNA 155 |
| MIR361 | MicroRNA 361 | VIP | Vasoactive Intestinal Peptide |
| MIR490 | MicroRNA 490 | CALCA | Calcitonin Related Polypeptide Alpha |
| MIR518A1 | MicroRNA 518a-1 | PRNP | Prion Protein |
| MIR532 | MicroRNA 532 | SST | Somatostatin |
| MIR15A | MicroRNA 15a | ESR1 | Estrogen Receptor 1 |
| BANCR | BRAF-Activated Non-Protein Coding RNA | PLAT | Plasminogen Activator, Tissue Type |
| MIR625 | MicroRNA 625 | SLC1A3 | Solute Carrier Family 1 Member 3 |
| MIR518D | MicroRNA 518d | IFNG | Interferon Gamma |
| CDR1-AS | CDR1 Antisense RNA | EPO | Erythropoietin |
| MTOR | Mechanistic Target Of Rapamycin Kinase | MAP1B | Microtubule Associated Protein 1B |
| MPO | Myeloperoxidase | ADIPOQ | Adiponectin, C1Q And Collagen Domain Containing |
| UGT1A7 | UDP Glucuronosyltransferase Family 1 Member A7 | LOC109461484 | Atrophin 1 Repeat Instability Region |
| PTEN | Phosphatase And Tensin Homolog | MIR17 | MicroRNA 17 |
| CYP1A1 | Cytochrome P450 Family 1 Subfamily A Member 1 | MIR30A | MicroRNA 30a |
| HAVCR2 | Hepatitis A Virus Cellular Receptor 2 | SYP | Synaptophysin |
| GSTM1 | Glutathione S-Transferase Mu 1 | CYP2C19 | Cytochrome P450 Family 2 Subfamily C Member 19 |
| TP53 | Tumor Protein P53 | NOS2 | Nitric Oxide Synthase 2 |
| KRAS | KRAS Proto-Oncogene, GTPase | IL2 | Interleukin 2 |
| BCL2 | BCL2 Apoptosis Regulator | SLC1A1 | Solute Carrier Family 1 Member 1 |
| CCL2 | C-C Motif Chemokine Ligand 2 | H2AC18 | H2A Clustered Histone 18 |
| NBN | Nibrin | MIR24-1 | MicroRNA 24-1 |
| FEN1 | Flap Structure-Specific Endonuclease 1 | PPARG | Peroxisome Proliferator Activated Receptor Gamma |
| EGLN2 | Egl-9 Family Hypoxia Inducible Factor 2 | CYP3A4 | Cytochrome P450 Family 3 Subfamily A Member 4 |
| GAP43 | Growth Associated Protein 43 | MIR144 | MicroRNA 144 |
| PDCD4 | Programmed Cell Death 4 | GRM1 | Glutamate Metabotropic Receptor 1 |
| RSPO2 | R-Spondin 2 | CCK | Cholecystokinin |
| MCAM | Melanoma Cell Adhesion Molecule | KDM4C | Lysine Demethylase 4C |
| RAP2A | RAP2A, Member Of RAS Oncogene Family | MIRLET7D | MicroRNA Let-7d |
| IBSP | Integrin Binding Sialoprotein | MIR125B1 | MicroRNA 125b-1 |
| EMC2 | ER Membrane Protein Complex Subunit 2 | MIR22 | MicroRNA 22 |
| HULC | Hepatocellular Carcinoma Up-Regulated Long Non-Coding RNA | SOD1 | Superoxide Dismutase 1 |
| LINC00261 | Long Intergenic Non-Protein Coding RNA 261 | NTRK1 | Neurotrophic Receptor Tyrosine Kinase 1 |
| DGCR5 | DiGeorge Syndrome Critical Region Gene 5 | PVALB | Parvalbumin |
| AFAP1-AS1 | AFAP1 Antisense RNA 1 | USH2A | Usherin |
| LINC00365 | Long Intergenic Non-Protein Coding RNA 365 | AQP4 | Aquaporin 4 |
| MIR486-2 | MicroRNA 486-2 | SCN1A | Sodium Voltage-Gated Channel Alpha Subunit 1 |
| LNCRNA-ATB | LncRNA Activated By TGF-Beta | CTNNB1 | Catenin Beta 1 |
|  |  | MIR338 | MicroRNA 338 |
|  |  | NGFR | Nerve Growth Factor Receptor |
|  |  | DLG4 | Discs Large MAGUK Scaffold Protein 4 |
|  |  | RELN | Reelin |
|  |  | IL17A | Interleukin 17A |
|  |  | NTF4 | Neurotrophin 4 |
|  |  | IL4 | Interleukin 4 |
|  |  | TRPV1 | Transient Receptor Potential Cation Channel Subfamily V Member 1 |
|  |  | CD4 | CD4 Molecule |
|  |  | AIF1 | Allograft Inflammatory Factor 1 |
|  |  | PPP1R1B | Protein Phosphatase 1 Regulatory Inhibitor Subunit 1B |
|  |  | MIR140 | MicroRNA 140 |
|  |  | MIR34C | MicroRNA 34c |
|  |  | MIR485 | MicroRNA 485 |
|  |  | ATN1 | Atrophin 1 |
|  |  | SYN1 | Synapsin I |
|  |  | CXCL8 | C-X-C Motif Chemokine Ligand 8 |
|  |  | GBA1 | Glucosylceramidase Beta 1 |
|  |  | NLRP3 | NLR Family Pyrin Domain Containing 3 |
|  |  | PRKN | Parkin RBR E3 Ubiquitin Protein Ligase |
|  |  | REST | RE1 Silencing Transcription Factor |
|  |  | CCR6 | C-C Motif Chemokine Receptor 6 |
|  |  | U2AF1 | U2 Small Nuclear RNA Auxiliary Factor 1 |
|  |  | MIR27A | MicroRNA 27a |
|  |  | MIR122 | MicroRNA 122 |
|  |  | MIR126 | MicroRNA 126 |
|  |  | MIR223 | MicroRNA 223 |
|  |  | MIR26B | MicroRNA 26b |
|  |  | MIR30E | MicroRNA 30e |
|  |  | MIR106B | MicroRNA 106b |
|  |  | MIR142 | MicroRNA 142 |
|  |  | MIR29A | MicroRNA 29a |
|  |  | MIR125A | MicroRNA 125a |
|  |  | MIR221 | MicroRNA 221 |
|  |  | MIR23A | MicroRNA 23a |
|  |  | MIR124-1 | MicroRNA 124-1 |
|  |  | MIR151A | MicroRNA 151a |
|  |  | MIR15B | MicroRNA 15b |
|  |  | MIR181C | MicroRNA 181c |
|  |  | MIR193A | MicroRNA 193a |
|  |  | MIR199A1 | MicroRNA 199a-1 |
|  |  | MIR335 | MicroRNA 335 |
|  |  | MIR342 | MicroRNA 342 |
|  |  | MIRLET7B | MicroRNA Let-7b |
|  |  | MIRLET7G | MicroRNA Let-7g |
|  |  | MIR128-1 | MicroRNA 128-1 |
|  |  | MIR181A1 | MicroRNA 181a-1 |
|  |  | MIR486-1 | MicroRNA 486-1 |
|  |  | GAL | Galanin And GMAP Prepropeptide |
|  |  | RBFOX3 | RNA Binding Fox-1 Homolog 3 |
|  |  | RTN4 | Reticulon 4 |
|  |  | IFNA1 | Interferon Alpha 1 |
|  |  | PSEN2 | Presenilin 2 |
|  |  | TFRC | Transferrin Receptor |
|  |  | ICAM1 | Intercellular Adhesion Molecule 1 |
|  |  | NTS | Neurotensin |
|  |  | GNB3 | G Protein Subunit Beta 3 |
|  |  | NCAM1 | Neural Cell Adhesion Molecule 1 |
|  |  | SPAST | Spastin |
|  |  | CYP1A2 | Cytochrome P450 Family 1 Subfamily A Member 2 |
|  |  | GRM7 | Glutamate Metabotropic Receptor 7 |
|  |  | MAPK1 | Mitogen-Activated Protein Kinase 1 |
|  |  | SERPINE1 | Serpin Family E Member 1 |
|  |  | SLC17A7 | Solute Carrier Family 17 Member 7 |
|  |  | HMOX1 | Heme Oxygenase 1 |
|  |  | ANKK1 | Ankyrin Repeat And Kinase Domain Containing 1 |
|  |  | BLOC1S1 | Biogenesis Of Lysosomal Organelles Complex 1 Subunit 1 |
|  |  | MIR195 | MicroRNA 195 |
|  |  | CTLA4 | Cytotoxic T-Lymphocyte Associated Protein 4 |
|  |  | CASP1 | Caspase 1 |
|  |  | ATF4 | Activating Transcription Factor 4 |
|  |  | HRH3 | Histamine Receptor H3 |
|  |  | GLRA1 | Glycine Receptor Alpha 1 |
|  |  | ATXN2 | Ataxin 2 |
|  |  | KNG1 | Kininogen 1 |
|  |  | CHRNA7 | Cholinergic Receptor Nicotinic Alpha 7 Subunit |
|  |  | NDP-AS1 | NDP Antisense RNA 1 |
|  |  | SCN2A | Sodium Voltage-Gated Channel Alpha Subunit 2 |
|  |  | HPRT1 | Hypoxanthine Phosphoribosyltransferase 1 |
|  |  | GSTP1 | Glutathione S-Transferase Pi 1 |
|  |  | HLA-A | Major Histocompatibility Complex, Class I, A |
|  |  | CHI3L1 | Chitinase 3 Like 1 |
|  |  | GPT | Glutamic--Pyruvic Transaminase |
|  |  | CACNA1C | Calcium Voltage-Gated Channel Subunit Alpha1 C |
|  |  | IL1R1 | Interleukin 1 Receptor Type 1 |
|  |  | CST3 | Cystatin C |
|  |  | AVP | Arginine Vasopressin |
|  |  | TACR1 | Tachykinin Receptor 1 |
|  |  | F2 | Coagulation Factor II, Thrombin |
|  |  | ADORA2A | Adenosine A2a Receptor |
|  |  | GSTM1 | Glutathione S-Transferase Mu 1 |
|  |  | ATP7B | ATPase Copper Transporting Beta |
|  |  | RTN4R | Reticulon 4 Receptor |
|  |  | PPARGC1A | PPARG Coactivator 1 Alpha |
|  |  | DNMT1 | DNA Methyltransferase 1 |
|  |  | IL1A | Interleukin 1 Alpha |
|  |  | MIR339 | MicroRNA 339 |
|  |  | RNU4ATAC | RNA, U4atac Small Nuclear |
|  |  | MIR659 | MicroRNA 659 |
|  |  | SLC12A6 | Solute Carrier Family 12 Member 6 |
|  |  | NR4A2 | Nuclear Receptor Subfamily 4 Group A Member 2 |
|  |  | SELE | Selectin E |
|  |  | WNK3 | WNK Lysine Deficient Protein Kinase 3 |
|  |  | SOD2 | Superoxide Dismutase 2 |
|  |  | GHRL | Ghrelin And Obestatin Prepropeptide |
|  |  | EGF | Epidermal Growth Factor |
|  |  | DNAJB1 | DnaJ Heat Shock Protein Family (Hsp40) Member B1 |
|  |  | GSTT1 | Glutathione S-Transferase Theta 1 |
|  |  | OLIG2 | Oligodendrocyte Transcription Factor 2 |
|  |  | ADRA2A | Adrenoceptor Alpha 2A |
|  |  | IDO1 | Indoleamine 2,3-Dioxygenase 1 |
|  |  | MAPK8 | Mitogen-Activated Protein Kinase 8 |
|  |  | ATM | ATM Serine/Threonine Kinase |
|  |  | PTGS2 | Prostaglandin-Endoperoxide Synthase 2 |
|  |  | SIRT1 | Sirtuin 1 |
|  |  | TGFB1 | Transforming Growth Factor Beta 1 |
|  |  | SHBG | Sex Hormone Binding Globulin |
|  |  | MMP2 | Matrix Metallopeptidase 2 |
|  |  | EGFR | Epidermal Growth Factor Receptor |
|  |  | ATXN1 | Ataxin 1 |
|  |  | LRRK2 | Leucine Rich Repeat Kinase 2 |
|  |  | SCN11A | Sodium Voltage-Gated Channel Alpha Subunit 11 |
|  |  | FRAXA | Fragile Site, Folic Acid Type, Rare, Fra(X)(Q27.3) A |
|  |  | PALS1 | Protein Associated With LIN7 1, MAGUK P55 Family Member |
|  |  | GAPDH | Glyceraldehyde-3-Phosphate Dehydrogenase |
|  |  | ADORA1 | Adenosine A1 Receptor |
|  |  | PICK1 | Protein Interacting With PRKCA 1 |
|  |  | ZNF804A | Zinc Finger Protein 804A |
|  |  | TERT | Telomerase Reverse Transcriptase |
|  |  | TNFRSF1A | TNF Receptor Superfamily Member 1A |
|  |  | CD36 | CD36 Molecule |
|  |  | SELL | Selectin L |
|  |  | PPIG | Peptidylprolyl Isomerase G |
|  |  | AR | Androgen Receptor |
|  |  | CHRNA3 | Cholinergic Receptor Nicotinic Alpha 3 Subunit |
|  |  | GABBR1 | Gamma-Aminobutyric Acid Type B Receptor Subunit 1 |
|  |  | ESR2 | Estrogen Receptor 2 |
|  |  | TUBA1A | Tubulin Alpha 1a |
|  |  | VCAM1 | Vascular Cell Adhesion Molecule 1 |
|  |  | NEUROD1 | Neuronal Differentiation 1 |
|  |  | SV2A | Synaptic Vesicle Glycoprotein 2A |
|  |  | FAAH | Fatty Acid Amide Hydrolase |
|  |  | PAH | Phenylalanine Hydroxylase |
|  |  | NF1 | Neurofibromin 1 |
|  |  | VWF | Von Willebrand Factor |
|  |  | ITIH4 | Inter-Alpha-Trypsin Inhibitor Heavy Chain 4 |
|  |  | L1CAM | L1 Cell Adhesion Molecule |
|  |  | FZD4 | Frizzled Class Receptor 4 |
|  |  | CDK20 | Cyclin Dependent Kinase 20 |
|  |  | TSPAN12 | Tetraspanin 12 |
|  |  | SCAPER | S-Phase Cyclin A Associated Protein In The ER |
|  |  | MIR34A | MicroRNA 34a |
|  |  | HCRTR1 | Hypocretin Receptor 1 |
|  |  | ATXN3 | Ataxin 3 |
|  |  | BDNF-AS | BDNF Antisense RNA |
|  |  | PGK1 | Phosphoglycerate Kinase 1 |
|  |  | CRYAA | Crystallin Alpha A |
|  |  | HNF1B | HNF1 Homeobox B |
|  |  | CRHR2 | Corticotropin Releasing Hormone Receptor 2 |
|  |  | MT-TL1 | Mitochondrially Encoded TRNA-Leu (UUA/G) 1 |
|  |  | SLC12A2 | Solute Carrier Family 12 Member 2 |
|  |  | SCARB1 | Scavenger Receptor Class B Member 1 |
|  |  | HIF1A | Hypoxia Inducible Factor 1 Subunit Alpha |
|  |  | PAX6 | Paired Box 6 |
|  |  | FOXO3 | Forkhead Box O3 |
|  |  | WWOX | WW Domain Containing Oxidoreductase |
|  |  | CALB1 | Calbindin 1 |
|  |  | MIR598 | MicroRNA 598 |
|  |  | MIR219A2 | MicroRNA 219a-2 |
|  |  | JUN | Jun Proto-Oncogene, AP-1 Transcription Factor Subunit |
|  |  | MIAT | Myocardial Infarction Associated Transcript |
|  |  | HAR1A | Highly Accelerated Region 1A |
|  |  | SLC6A1 | Solute Carrier Family 6 Member 1 |
|  |  | SACS | Sacsin Molecular Chaperone |
|  |  | PRKCA | Protein Kinase C Alpha |
|  |  | SLC6A9 | Solute Carrier Family 6 Member 9 |
|  |  | ERBB4 | Erb-B2 Receptor Tyrosine Kinase 4 |
|  |  | ITPR1 | Inositol 1,4,5-Trisphosphate Receptor Type 1 |
|  |  | FXN | Frataxin |
|  |  | MAF | MAF BZIP Transcription Factor |
|  |  | SRD5A1 | Steroid 5 Alpha-Reductase 1 |
|  |  | TF | Transferrin |
|  |  | ADCYAP1 | Adenylate Cyclase Activating Polypeptide 1 |
|  |  | EGR1 | Early Growth Response 1 |
|  |  | TOR1A | Torsin Family 1 Member A |
|  |  | GRIN2C | Glutamate Ionotropic Receptor NMDA Type Subunit 2C |
|  |  | NOS3 | Nitric Oxide Synthase 3 |
|  |  | SORL1 | Sortilin Related Receptor 1 |
|  |  | CYP1A1 | Cytochrome P450 Family 1 Subfamily A Member 1 |
|  |  | AUTS2 | Activator Of Transcription And Developmental Regulator AUTS2 |
|  |  | PRX | Periaxin |
|  |  | SLC32A1 | Solute Carrier Family 32 Member 1 |
|  |  | HMBS | Hydroxymethylbilane Synthase |
|  |  | AGTR1 | Angiotensin II Receptor Type 1 |
|  |  | ATP13A2 | ATPase Cation Transporting 13A2 |
|  |  | WFS1 | Wolframin ER Transmembrane Glycoprotein |
|  |  | CHRNB4 | Cholinergic Receptor Nicotinic Beta 4 Subunit |
|  |  | SMPD1 | Sphingomyelin Phosphodiesterase 1 |
|  |  | TPI1 | Triosephosphate Isomerase 1 |
|  |  | PINK1 | PTEN Induced Kinase 1 |
|  |  | CALB2 | Calbindin 2 |
|  |  | FOXP2 | Forkhead Box P2 |
|  |  | ADRA2C | Adrenoceptor Alpha 2C |
|  |  | CDK5 | Cyclin Dependent Kinase 5 |
|  |  | CDC42 | Cell Division Cycle 42 |
|  |  | LGI1 | Leucine Rich Glioma Inactivated 1 |
|  |  | LTA | Lymphotoxin Alpha |
|  |  | GRP | Gastrin Releasing Peptide |
|  |  | NPC1 | NPC Intracellular Cholesterol Transporter 1 |
|  |  | MET | MET Proto-Oncogene, Receptor Tyrosine Kinase |
|  |  | CSF1R | Colony Stimulating Factor 1 Receptor |
|  |  | NOTCH3 | Notch Receptor 3 |
|  |  | ADORA2B | Adenosine A2b Receptor |
|  |  | ADORA3 | Adenosine A3 Receptor |
|  |  | AVPR1A | Arginine Vasopressin Receptor 1A |
|  |  | GLRB | Glycine Receptor Beta |
|  |  | LOC109504725 | Androgen Receptor Repeat Instability Region |
|  |  | CRHBP | Corticotropin Releasing Hormone Binding Protein |
|  |  | ADA | Adenosine Deaminase |
|  |  | CNR2 | Cannabinoid Receptor 2 |
|  |  | RGS2 | Regulator Of G Protein Signaling 2 |
|  |  | IL33 | Interleukin 33 |
|  |  | HTR2B | 5-Hydroxytryptamine Receptor 2B |
|  |  | SIGMAR1 | Sigma Non-Opioid Intracellular Receptor 1 |
|  |  | HTR1D | 5-Hydroxytryptamine Receptor 1D |
|  |  | GCH1 | GTP Cyclohydrolase 1 |
|  |  | TRH | Thyrotropin Releasing Hormone |
|  |  | ABAT | 4-Aminobutyrate Aminotransferase |
|  |  | NTRK3 | Neurotrophic Receptor Tyrosine Kinase 3 |
|  |  | GYPE | Glycophorin E (MNS Blood Group) |
|  |  | PTGDS | Prostaglandin D2 Synthase |
|  |  | NPPC | Natriuretic Peptide C |
|  |  | NR1H2 | Nuclear Receptor Subfamily 1 Group H Member 2 |
|  |  | P2RX7 | Purinergic Receptor P2X 7 |
|  |  | CYP3A5 | Cytochrome P450 Family 3 Subfamily A Member 5 |
|  |  | IGF1R | Insulin Like Growth Factor 1 Receptor |
|  |  | ADAM10 | ADAM Metallopeptidase Domain 10 |
|  |  | MAP2K1 | Mitogen-Activated Protein Kinase Kinase 1 |
|  |  | HSP90AA1 | Heat Shock Protein 90 Alpha Family Class A Member 1 |
|  |  | SHH | Sonic Hedgehog Signaling Molecule |
|  |  | CASK | Calcium/Calmodulin Dependent Serine Protein Kinase |
|  |  | IGF2 | Insulin Like Growth Factor 2 |
|  |  | IRS1 | Insulin Receptor Substrate 1 |
|  |  | PPT1 | Palmitoyl-Protein Thioesterase 1 |
|  |  | SEMA3A | Semaphorin 3A |
|  |  | IAPP | Islet Amyloid Polypeptide |
|  |  | TCHP | Trichoplein Keratin Filament Binding |
|  |  | MIR7-1 | MicroRNA 7-1 |
|  |  | MIR383 | MicroRNA 383 |
|  |  | ADRB2 | Adrenoceptor Beta 2 |
|  |  | GABRG2 | Gamma-Aminobutyric Acid Type A Receptor Subunit Gamma2 |
|  |  | LIF | LIF Interleukin 6 Family Cytokine |
|  |  | IL1RN | Interleukin 1 Receptor Antagonist |
|  |  | SCN3A | Sodium Voltage-Gated Channel Alpha Subunit 3 |
|  |  | SCN4A | Sodium Voltage-Gated Channel Alpha Subunit 4 |
|  |  | LEPR | Leptin Receptor |
|  |  | ASTN2 | Astrotactin 2 |
|  |  | TBP | TATA-Box Binding Protein |
|  |  | OTX2 | Orthodenticle Homeobox 2 |
|  |  | GNB5 | G Protein Subunit Beta 5 |
|  |  | ATXN7 | Ataxin 7 |
|  |  | MIR509-1 | MicroRNA 509-1 |
|  |  | GRIN2D | Glutamate Ionotropic Receptor NMDA Type Subunit 2D |
|  |  | RYR1 | Ryanodine Receptor 1 |
|  |  | KARS1 | Lysyl-TRNA Synthetase 1 |
|  |  | TSC1 | TSC Complex Subunit 1 |
|  |  | NSD1 | Nuclear Receptor Binding SET Domain Protein 1 |
|  |  | MMACHC | Metabolism Of Cobalamin Associated C |
|  |  | TWNK | Twinkle MtDNA Helicase |
|  |  | MTR | 5-Methyltetrahydrofolate-Homocysteine Methyltransferase |
|  |  | TTF2 | Transcription Termination Factor 2 |
|  |  | SLC6A5 | Solute Carrier Family 6 Member 5 |
|  |  | OPN4 | Opsin 4 |
|  |  | SPG11 | SPG11 Vesicle Trafficking Associated, Spatacsin |
|  |  | UBE3A | Ubiquitin Protein Ligase E3A |
|  |  | OPRK1 | Opioid Receptor Kappa 1 |
|  |  | CLN3 | CLN3 Lysosomal/Endosomal Transmembrane Protein, Battenin |
|  |  | ACTB | Actin Beta |
|  |  | SIRT5 | Sirtuin 5 |
|  |  | XBP1 | X-Box Binding Protein 1 |
|  |  | CHL1 | Cell Adhesion Molecule L1 Like |
|  |  | MCPH1 | Microcephalin 1 |
|  |  | CAT | Catalase |
|  |  | TCF4 | Transcription Factor 4 |
|  |  | AARS1 | Alanyl-TRNA Synthetase 1 |
|  |  | LOC106627981 | GBA Recombination Region |
|  |  | VDR | Vitamin D Receptor |
|  |  | IL4R | Interleukin 4 Receptor |
|  |  | NR3C2 | Nuclear Receptor Subfamily 3 Group C Member 2 |
|  |  | GLO1 | Glyoxalase I |
|  |  | ALAD | Aminolevulinate Dehydratase |
|  |  | HOMER1 | Homer Scaffold Protein 1 |
|  |  | HRH1 | Histamine Receptor H1 |
|  |  | NPPA | Natriuretic Peptide A |
|  |  | APOD | Apolipoprotein D |
|  |  | ARX | Aristaless Related Homeobox |
|  |  | XK | X-Linked Kx Blood Group Antigen, Kell And VPS13A Binding Protein |
|  |  | IL2RA | Interleukin 2 Receptor Subunit Alpha |
|  |  | CYP27A1 | Cytochrome P450 Family 27 Subfamily A Member 1 |
|  |  | NAGLU | N-Acetyl-Alpha-Glucosaminidase |
|  |  | VGF | VGF Nerve Growth Factor Inducible |
|  |  | LOC109504728 | Chromosome 9 Open Reading Frame 72 Repeat Instability Region |
|  |  | GNAS | GNAS Complex Locus |
|  |  | HRAS | HRas Proto-Oncogene, GTPase |
|  |  | DPP4 | Dipeptidyl Peptidase 4 |
|  |  | HDAC1 | Histone Deacetylase 1 |
|  |  | RHOA | Ras Homolog Family Member A |
|  |  | CYBB | Cytochrome B-245 Beta Chain |
|  |  | GLI1 | GLI Family Zinc Finger 1 |
|  |  | CFH | Complement Factor H |
|  |  | CXCL1 | C-X-C Motif Chemokine Ligand 1 |
|  |  | CD68 | CD68 Molecule |
|  |  | MIR205 | MicroRNA 205 |
|  |  | HSD17B10 | Hydroxysteroid 17-Beta Dehydrogenase 10 |
|  |  | GALR3 | Galanin Receptor 3 |
|  |  | GALR1 | Galanin Receptor 1 |
|  |  | SLC6A13 | Solute Carrier Family 6 Member 13 |
|  |  | KCNN3 | Potassium Calcium-Activated Channel Subfamily N Member 3 |
|  |  | ATP1A3 | ATPase Na+/K+ Transporting Subunit Alpha 3 |
|  |  | HMGCR | 3-Hydroxy-3-Methylglutaryl-CoA Reductase |
|  |  | PDE5A | Phosphodiesterase 5A |
|  |  | BSCL2 | BSCL2 Lipid Droplet Biogenesis Associated, Seipin |
|  |  | SYT2 | Synaptotagmin 2 |
|  |  | SLC16A2 | Solute Carrier Family 16 Member 2 |
|  |  | ADCYAP1R1 | ADCYAP Receptor Type I |
|  |  | WNT1 | Wnt Family Member 1 |
|  |  | CYP2E1 | Cytochrome P450 Family 2 Subfamily E Member 1 |
|  |  | ADRA1D | Adrenoceptor Alpha 1D |
|  |  | UCN | Urocortin |
|  |  | BRAF | B-Raf Proto-Oncogene, Serine/Threonine Kinase |
|  |  | PIK3C2A | Phosphatidylinositol-4-Phosphate 3-Kinase Catalytic Subunit Type 2 Alpha |
|  |  | HLA-B | Major Histocompatibility Complex, Class I, B |
|  |  | CNTNAP1 | Contactin Associated Protein 1 |
|  |  | TCN2 | Transcobalamin 2 |
|  |  | SDHB | Succinate Dehydrogenase Complex Iron Sulfur Subunit B |
|  |  | NPPB | Natriuretic Peptide B |
|  |  | NKX2-1 | NK2 Homeobox 1 |
|  |  | GAST | Gastrin |
|  |  | HP | Haptoglobin |
|  |  | GRPR | Gastrin Releasing Peptide Receptor |
|  |  | HSPG2 | Heparan Sulfate Proteoglycan 2 |
|  |  | MT-ND3 | Mitochondrially Encoded NADH:Ubiquinone Oxidoreductase Core Subunit 3 |
|  |  | GABRB2 | Gamma-Aminobutyric Acid Type A Receptor Subunit Beta2 |
|  |  | CHRM2 | Cholinergic Receptor Muscarinic 2 |
|  |  | RAI1 | Retinoic Acid Induced 1 |
|  |  | SCT | Secretin |
|  |  | MSH2 | MutS Homolog 2 |
|  |  | MLH1 | MutL Homolog 1 |
|  |  | HCRTR2 | Hypocretin Receptor 2 |
|  |  | ADARB1 | Adenosine Deaminase RNA Specific B1 |
|  |  | NRGN | Neurogranin |
|  |  | C12orf4 | Chromosome 12 Open Reading Frame 4 |
|  |  | TUBB3 | Tubulin Beta 3 Class III |
|  |  | TPPP | Tubulin Polymerization Promoting Protein |
|  |  | CHRM3 | Cholinergic Receptor Muscarinic 3 |
|  |  | ADRA1B | Adrenoceptor Alpha 1B |
|  |  | SLC1A4 | Solute Carrier Family 1 Member 4 |
|  |  | PHF8 | PHD Finger Protein 8 |
|  |  | CNTFR | Ciliary Neurotrophic Factor Receptor |
|  |  | IFNA2 | Interferon Alpha 2 |
|  |  | RPLP0 | Ribosomal Protein Lateral Stalk Subunit P0 |
|  |  | GRIN3A | Glutamate Ionotropic Receptor NMDA Type Subunit 3A |
|  |  | SEPTIN2 | Septin 2 |
|  |  | SLC9A6 | Solute Carrier Family 9 Member A6 |
|  |  | CACNA1H | Calcium Voltage-Gated Channel Subunit Alpha1 H |
|  |  | SCN2B | Sodium Voltage-Gated Channel Beta Subunit 2 |
|  |  | ADAM12 | ADAM Metallopeptidase Domain 12 |
|  |  | C19orf12 | Chromosome 19 Open Reading Frame 12 |
|  |  | SOS1 | SOS Ras/Rac Guanine Nucleotide Exchange Factor 1 |
|  |  | PDE4D | Phosphodiesterase 4D |
|  |  | NEGR1 | Neuronal Growth Regulator 1 |
|  |  | AGT | Angiotensinogen |
|  |  | HTR7 | 5-Hydroxytryptamine Receptor 7 |
|  |  | NRCAM | Neuronal Cell Adhesion Molecule |
|  |  | SDHD | Succinate Dehydrogenase Complex Subunit D |
|  |  | TIMM8A | Translocase Of Inner Mitochondrial Membrane 8A |
|  |  | CA10 | Carbonic Anhydrase 10 |
|  |  | CLTC | Clathrin Heavy Chain |
|  |  | NSUN2 | NOP2/Sun RNA Methyltransferase 2 |
|  |  | KMT5B | Lysine Methyltransferase 5B |
|  |  | DLGAP1 | DLG Associated Protein 1 |
|  |  | HTR6 | 5-Hydroxytryptamine Receptor 6 |
|  |  | CP | Ceruloplasmin |
|  |  | P2RX4 | Purinergic Receptor P2X 4 |
|  |  | CNOT1 | CCR4-NOT Transcription Complex Subunit 1 |
|  |  | NEMF | Nuclear Export Mediator Factor |
|  |  | SGCG | Sarcoglycan Gamma |
|  |  | HDAC9 | Histone Deacetylase 9 |
|  |  | LPL | Lipoprotein Lipase |
|  |  | CNP | 2',3'-Cyclic Nucleotide 3' Phosphodiesterase |
|  |  | DNM1 | Dynamin 1 |
|  |  | SCN5A | Sodium Voltage-Gated Channel Alpha Subunit 5 |
|  |  | ADSL | Adenylosuccinate Lyase |
|  |  | HEXA | Hexosaminidase Subunit Alpha |
|  |  | KLK3 | Kallikrein Related Peptidase 3 |
|  |  | TPK1 | Thiamin Pyrophosphokinase 1 |
|  |  | AMACR | Alpha-Methylacyl-CoA Racemase |
|  |  | ATF3 | Activating Transcription Factor 3 |
|  |  | SLC17A5 | Solute Carrier Family 17 Member 5 |
|  |  | SORT1 | Sortilin 1 |
|  |  | NPC2 | NPC Intracellular Cholesterol Transporter 2 |
|  |  | NAB2 | NGFI-A Binding Protein 2 |
|  |  | PREP | Prolyl Endopeptidase |
|  |  | PTGES | Prostaglandin E Synthase |
|  |  | MT-TK | Mitochondrially Encoded TRNA-Lys (AAA/G) |
|  |  | GABBR2 | Gamma-Aminobutyric Acid Type B Receptor Subunit 2 |
|  |  | GGT1 | Gamma-Glutamyltransferase 1 |
|  |  | PIK3CG | Phosphatidylinositol-4,5-Bisphosphate 3-Kinase Catalytic Subunit Gamma |
|  |  | ADRB1 | Adrenoceptor Beta 1 |
|  |  | ALDH5A1 | Aldehyde Dehydrogenase 5 Family Member A1 |
|  |  | GRIK1 | Glutamate Ionotropic Receptor Kainate Type Subunit 1 |
|  |  | PLD2 | Phospholipase D2 |
|  |  | CCR3 | C-C Motif Chemokine Receptor 3 |
|  |  | CNTN2 | Contactin 2 |
|  |  | GATM | Glycine Amidinotransferase |
|  |  | UCP2 | Uncoupling Protein 2 |
|  |  | FADS2 | Fatty Acid Desaturase 2 |
|  |  | UBB | Ubiquitin B |
|  |  | FADS1 | Fatty Acid Desaturase 1 |
|  |  | SLC6A12 | Solute Carrier Family 6 Member 12 |
|  |  | GALR2 | Galanin Receptor 2 |
|  |  | PCDH15 | Protocadherin Related 15 |
|  |  | SNAP29 | Synaptosome Associated Protein 29 |
|  |  | SLC6A11 | Solute Carrier Family 6 Member 11 |
|  |  | GHRH | Growth Hormone Releasing Hormone |
|  |  | PENK | Proenkephalin |
|  |  | HLA-S | Major Histocompatibility Complex, Class I, S (Pseudogene) |
|  |  | GHSR | Growth Hormone Secretagogue Receptor |
|  |  | PEPD | Peptidase D |
|  |  | DIO2 | Iodothyronine Deiodinase 2 |
|  |  | SLC9A7 | Solute Carrier Family 9 Member A7 |
|  |  | IGFBP2 | Insulin Like Growth Factor Binding Protein 2 |
|  |  | FGF14 | Fibroblast Growth Factor 14 |
|  |  | ATXN8OS | ATXN8 Opposite Strand LncRNA |
|  |  | OPA3 | Outer Mitochondrial Membrane Lipid Metabolism Regulator OPA3 |
|  |  | TNFRSF1B | TNF Receptor Superfamily Member 1B |
|  |  | FKBP4 | FKBP Prolyl Isomerase 4 |
|  |  | ADRA2B | Adrenoceptor Alpha 2B |
|  |  | NDUFS4 | NADH:Ubiquinone Oxidoreductase Subunit S4 |
|  |  | CPLX1 | Complexin 1 |
|  |  | IL12B | Interleukin 12B |
|  |  | MT-ND2 | Mitochondrially Encoded NADH:Ubiquinone Oxidoreductase Core Subunit 2 |
|  |  | GH1 | Growth Hormone 1 |
|  |  | PCDH19 | Protocadherin 19 |
|  |  | COL4A1 | Collagen Type IV Alpha 1 Chain |
|  |  | ACADS | Acyl-CoA Dehydrogenase Short Chain |
|  |  | EMX2 | Empty Spiracles Homeobox 2 |
|  |  | MT-ND5 | Mitochondrially Encoded NADH:Ubiquinone Oxidoreductase Core Subunit 5 |
|  |  | PTPN11 | Protein Tyrosine Phosphatase Non-Receptor Type 11 |
|  |  | INSR | Insulin Receptor |
|  |  | CFTR | CF Transmembrane Conductance Regulator |
|  |  | CACNA1G | Calcium Voltage-Gated Channel Subunit Alpha1 G |
|  |  | GLS | Glutaminase |
|  |  | ATP2A1 | ATPase Sarcoplasmic/Endoplasmic Reticulum Ca2+ Transporting 1 |
|  |  | CYP19A1 | Cytochrome P450 Family 19 Subfamily A Member 1 |
|  |  | GALK1 | Galactokinase 1 |
|  |  | PTGER2 | Prostaglandin E Receptor 2 |
|  |  | CUL3 | Cullin 3 |
|  |  | FSHR | Follicle Stimulating Hormone Receptor |
|  |  | OAT | Ornithine Aminotransferase |
|  |  | SLC12A1 | Solute Carrier Family 12 Member 1 |
|  |  | CACNA1I | Calcium Voltage-Gated Channel Subunit Alpha1 I |
|  |  | CTCF | CCCTC-Binding Factor |
|  |  | FGR | FGR Proto-Oncogene, Src Family Tyrosine Kinase |
|  |  | NT5C2 | 5'-Nucleotidase, Cytosolic II |
|  |  | SCN3B | Sodium Voltage-Gated Channel Beta Subunit 3 |
|  |  | ADAR | Adenosine Deaminase RNA Specific |
|  |  | ALDH4A1 | Aldehyde Dehydrogenase 4 Family Member A1 |
|  |  | ATP2B1 | ATPase Plasma Membrane Ca2+ Transporting 1 |
|  |  | FOXA2 | Forkhead Box A2 |
|  |  | SCN1B | Sodium Voltage-Gated Channel Beta Subunit 1 |
|  |  | SLC22A4 | Solute Carrier Family 22 Member 4 |
|  |  | TRPM8 | Transient Receptor Potential Cation Channel Subfamily M Member 8 |
|  |  | ATP2C1 | ATPase Secretory Pathway Ca2+ Transporting 1 |
|  |  | CFD | Complement Factor D |
|  |  | PDE4A | Phosphodiesterase 4A |
|  |  | SCN4B | Sodium Voltage-Gated Channel Beta Subunit 4 |
|  |  | SLC22A2 | Solute Carrier Family 22 Member 2 |
|  |  | IL18R1 | Interleukin 18 Receptor 1 |
|  |  | NISCH | Nischarin |
|  |  | ITSN1 | Intersectin 1 |
|  |  | ROBO3 | Roundabout Guidance Receptor 3 |
|  |  | RAB10 | RAB10, Member RAS Oncogene Family |
|  |  | SLC38A1 | Solute Carrier Family 38 Member 1 |
|  |  | DHRS9 | Dehydrogenase/Reductase 9 |
|  |  | HIP1 | Huntingtin Interacting Protein 1 |
|  |  | MTDH | Metadherin |
|  |  | RABEP1 | Rabaptin, RAB GTPase Binding Effector Protein 1 |
|  |  | SCGB1A1 | Secretoglobin Family 1A Member 1 |
|  |  | IL1RAPL2 | Interleukin 1 Receptor Accessory Protein Like 2 |
|  |  | PPL | Periplakin |
|  |  | SEPTIN9 | Septin 9 |
|  |  | MT3 | Metallothionein 3 |
|  |  | CBLN2 | Cerebellin 2 Precursor |
|  |  | SERINC2 | Serine Incorporator 2 |
|  |  | CHRFAM7A | CHRNA7 (Exons 5-10) And FAM7A (Exons A-E) Fusion |
|  |  | HULC | Hepatocellular Carcinoma Up-Regulated Long Non-Coding RNA |
|  |  | LOC109461479 | Huntingtin Repeat Instability Region |
|  |  | LOC109610631 | Aristaless Related Homeobox Polyalanine Expansion Region |
|  |  | LOC109580097 | Mab-21 Like 1 Repeat Instability Region |
|  |  | FOLH1 | Folate Hydrolase 1 |
|  |  | MMP7 | Matrix Metallopeptidase 7 |
|  |  | CDK5R1 | Cyclin Dependent Kinase 5 Regulatory Subunit 1 |
|  |  | BCYRN1 | Brain Cytoplasmic RNA 1 |
|  |  | KIF11 | Kinesin Family Member 11 |
|  |  | PRKCE | Protein Kinase C Epsilon |
|  |  | PC | Pyruvate Carboxylase |
|  |  | UROD | Uroporphyrinogen Decarboxylase |
|  |  | MYT1L | Myelin Transcription Factor 1 Like |
|  |  | RETN | Resistin |
|  |  | EFHC2 | EF-Hand Domain Containing 2 |
|  |  | LOC108228197 | LCR2q21.1 Proximal Recombination Region |
|  |  | LOC108228198 | LCR2q21.1 Distal Recombination Region |
|  |  | SLC1A7 | Solute Carrier Family 1 Member 7 |
|  |  | PRDX1 | Peroxiredoxin 1 |
|  |  | SPTLC3 | Serine Palmitoyltransferase Long Chain Base Subunit 3 |
|  |  | ATP1A1 | ATPase Na+/K+ Transporting Subunit Alpha 1 |
|  |  | KL | Klotho |
|  |  | TPO | Thyroid Peroxidase |
|  |  | NTN1 | Netrin 1 |
|  |  | TNFSF10 | TNF Superfamily Member 10 |
|  |  | PREX1 | Phosphatidylinositol-3,4,5-Trisphosphate Dependent Rac Exchange Factor 1 |
|  |  | ACTL6B | Actin Like 6B |
|  |  | MT-ND4 | Mitochondrially Encoded NADH:Ubiquinone Oxidoreductase Core Subunit 4 |
|  |  | MT-ATP8 | Mitochondrially Encoded ATP Synthase Membrane Subunit 8 |
|  |  | MT-ND1 | Mitochondrially Encoded NADH:Ubiquinone Oxidoreductase Core Subunit 1 |
|  |  | SYK | Spleen Associated Tyrosine Kinase |
