# Supplementary material for: A novel FCTF evaluation and prediction model for food efficacy based on association rule mining
Source: Front Nutr. 2023 Aug 28;10:1170084. doi: 10.3389/fnut.2023.1170084 (PMC10493461; doi:10.3389/fnut.2023.1170084)
Supplement: Supplementary file 5 [file Table_5.docx]

**Supplemental Table S5 Vina scores and cavity information of the docking simulation pose for each targeted protein and components**

| **Component-Target** | **Target (PDB ID)** | **Vina score** | **Cavity size** | **Center (x, y, z)** | **Docking size (x, y, z)** |
| --- | --- | --- | --- | --- | --- |
| Citronellal-ACE | 1O86 | -5.2 | 9438 | 48, 30, 42 | 35, 33, 35 |
| Trans-Nerolidol-PTGS2 | 5IKT | -7.3 | 3988 | 171, 223, 221 | 29, 29, 30 |
| Linalool-PTGS2 | 5IKT | -6 | 1973 | 170, 191, 190 | 24, 18, 18 |
| Geraniol-PTGS2 | 5IKT | -6 | 3988 | 171, 223, 221 | 29, 29, 30 |
| α-Terpineol-CYP2C19 | 4GQS | -6 | 9071 | -61, 40, -4 | 34, 35, 35 |
| Cadinene-CYP2C19 | 4GQS | -8.1 | 2127 | -95, 0, -56 | 28, 30, 35 |
| α-Pinene-CYP2C19 | 4GQS | -6.2 | 7810 | -80, 21, -38 | 28, 35, 35 |
